# Total Synthesis of GE81112A: An Orthoester-Based Approach

**DOI:** 10.1021/acs.joc.3c00094

**Published:** 2023-04-06

**Authors:** Scherin Fayad, Ardalan Jafari, Sören M. M. Schuler, Michael Kurz, Oliver Plettenburg, Peter E. Hammann, Armin Bauer, Gerrit Jürjens, Christoph Pöverlein

**Affiliations:** † Sanofi-Aventis Deutschland GmbH, R&D, Integrated Drug Discovery, Industriepark Hoechst, 65926 Frankfurt am Main, Germany; ‡ Helmholtz Zentrum München, Deutsches Forschungszentrum für Gesundheit und Umwelt (GmbH), Institut für Medizinalchemie, 30167 Hannover, Germany; § Fraunhofer Institute for Molecular Biology and Applied Ecology IME, Branch for Bioresources, 35392 Giessen, Germany; ∥ Evotec International GmbH, 37079 Göttingen, Germany; ⊥ Sanofi-Aventis Deutschland GmbH, R&D, Infectious Diseases, Industriepark Hoechst, 65926 Frankfurt am Main, Germany

## Abstract

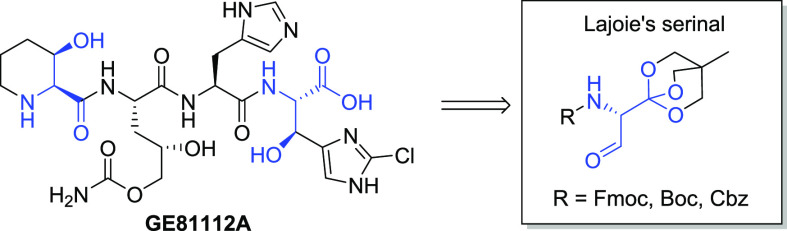

The GE81112 series, consisting of three naturally occurring tetrapeptides and synthetic derivatives, is evaluated as a potential lead structure for the development of a new antibacterial drug. Although the first total synthesis of GE81112A reported by our group provided sufficient amounts of material for an initial in depth biological profiling of the compound, improvements of the routes toward the key building blocks were needed for further upscaling and structure–activity relationship studies. The major challenges identified were poor stereoselectivity in the synthesis of the *C*-terminal β-hydroxy histidine intermediate and a concise access to all four isomers of the 3-hydroxy pipecolic acid. Herein, we report a second-generation synthesis of GE81112A, which is also applicable to access further representatives of this series. Based on Lajoie’s *ortho*-ester-protected serine aldehydes as key building blocks, the described route provides both a satisfactory improvement in stereoselectivity of the β-hydroxy histidine intermediate synthesis and a stereoselective approach toward both orthogonally protected *cis* and *trans*-3-hydroxy pipecolic acid.

## Introduction

**Figure 1:**
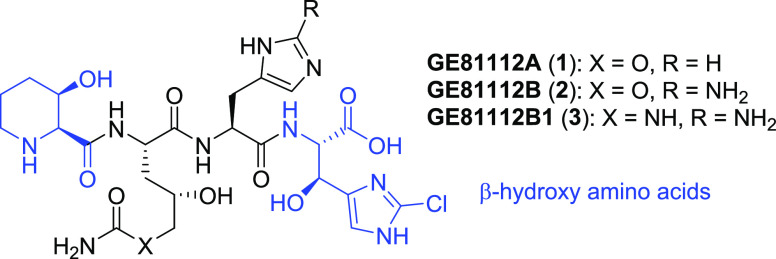
Structure of the GE81112 series.

The worldwide problem of increasing multidrug resistance is compounded by the decline in new classes of antimicrobials entering into clinical practice. This fact led to renewed interest in novel scaffolds as starting points for new antimicrobial agents. GE81112A (**1**),^1,2^ a nonribosomally synthesized tetrapeptide, and its congeners GE81112B (**2**) and GE81112B1 (**3**) (Figure 1) represent such a novel chemical structure. Since the anti-Gram-negative activity is based on ribosome targeting,^2,3^ the GE81112 series gained high interest as a potential lead.

The first total synthesis of GE81112A (**1**) accomplished by our group led to the revision of the originally published structure^4^ and provided first batches of **1** on a 100 mg scale for in depth biological profiling.^5^ Structure–activity relationships were expanded by the Renata group which developed a concise total synthesis of GE81112B1 (**3**) and 10 non-natural analogues thereof.^6,7^ After careful analysis of our (first-generation) route toward **1** and Renata’s synthesis of **3**, we identified the syntheses of both β-hydroxy amino acid moieties as major areas to be improved.

**Scheme 1:**
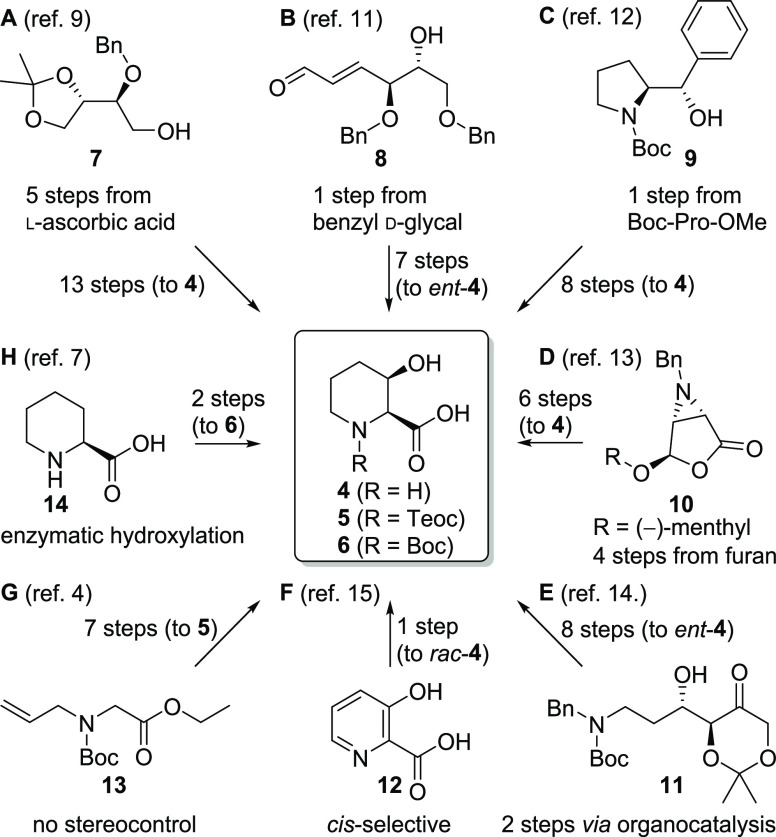
Selected Examples of Reported Syntheses of *cis*-3-Hydroxy Pipecolic Acid Derivatives

**Scheme 2:**
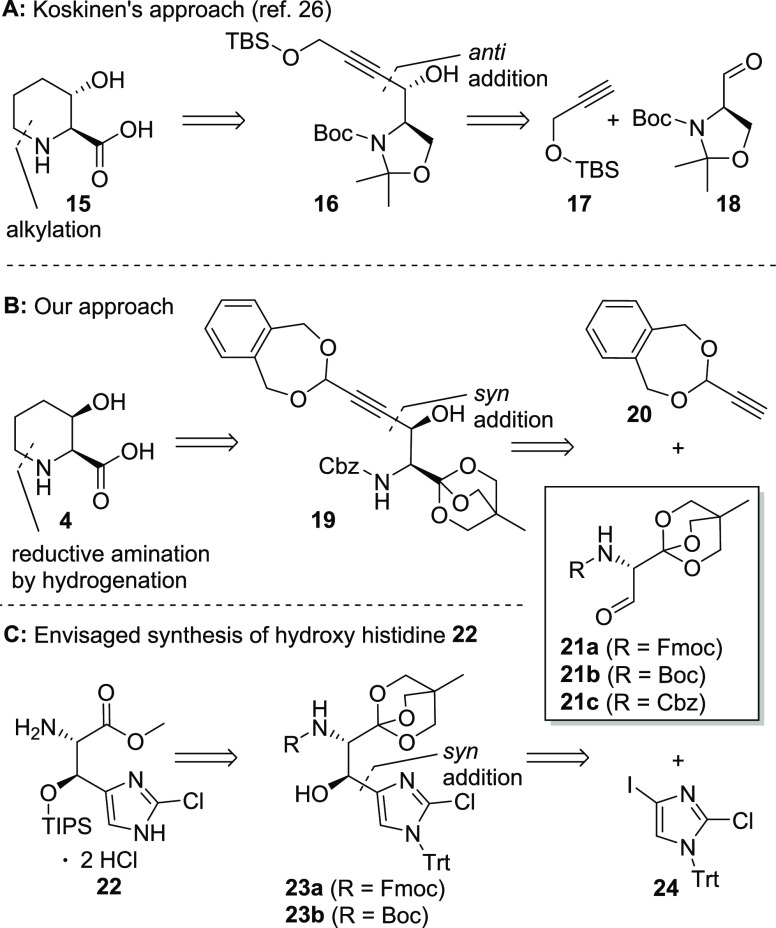
Retrosynthetic Analysis of *trans*- and *cis*-3-Hydroxy Pipecolic Acids 15 (A) and 4 (B) and β-Hydroxy Histidine Building Block 22 (C)

While a nonstereoselective approach toward all four stereoisomers of the 3-hydroxy pipecolic acid moiety was considered acceptable in the first total synthesis of GE81112A with regard to ambiguity concerning the absolute configuration and planned SAR studies (Scheme 1, entry G), an adapted route featuring Teoc-protected (2*S*,3*R*)-hydroxy pipecolic acid **5** in a stereocontrolled manner was required for scale-up and synthesis of derivatives. In the same line, poor stereoselectivity experienced in the synthesis of the *C*-terminal β-hydroxy histidine building block **22** using Garner’s aldehyde (*dr* 2:1) was identified as an additional issue (Scheme 2). In the total synthesis of GE81112B1 (**3**) by the Renata group,^6,7^ (2*S*,3*R*)-hydroxy pipecolic acid derivative **6** was prepared from (2*S*)-pipecolic acid (**14**) by chemoenzymatic oxidation^8^ in two steps (Scheme 1, entry H).

Our strategy for a concise stereoselective synthesis of GE81112A (**1**) is based on a detailed analysis of its revised structure revealing an intriguing feature: both the β-hydroxy pipecolic acid and the β-hydroxy histidine display the same *threo* configuration. Therefore, we reasoned that it should be feasible to deliver intermediates for both moieties from one common building block defining the required *threo* configuration in a highly selective manner. We first focused on the β-hydroxy pipecolic acid targeting the Teoc-protected *cis*-(2*S*,3*R*)-configured product **5** as key intermediate. Due to the total synthesis of GE81112B1 (**3**) by the Renata group, Boc-protected compound **6** became a target as well. Besides high yields, technical feasibility, and low number of synthetic steps, the high degree of stereoselectivity and enantiomeric excess (*ee*) represent important criteria for such an alternative route. While numerous synthetic protocols for 3-hydroxy pipecolic acid derivatives had been reported, our search for *cis*-selective methods resulted in surprisingly few convincing examples (Scheme 1): In a synthesis from Chavan et al. starting from readily available chiral pool material l-ascorbic acid (Scheme 1, entry A^9^), the advantage of cheap starting materials is completely outweighed by in total 18 synthetic steps and one racemization event. Syntheses from d-sugars such as d-glucose (not shown)^10^ or d-glycals (Scheme 1, entry B^11^) are attractive for the enantiomeric *cis*-(2*R*,3*S*)-configured product *ent*-**4**, but unsuitable for our target molecules. The reported syntheses from Cochi et al. utilizing enantioselective ring expansion of proline-derived intermediate **9** (Scheme 1, entry C^12^), from Chavan et al. with a key aziridine intermediate **10** (Scheme 1, entry D^13^), and from Marjanovic et al. using dioxone intermediate **11** accessible via organocatalysis (Scheme 1, entry E^14^) gave access to **4** in up to 10 synthetic steps. The shortest route employs hydrogenation of 3-hydroxypicolic acid (**12**) affording *cis*-selectively *rac*-**4** (Scheme 1, entry F^15^), but separation of the enantiomers remains an issue.^16^

In comparison, synthetic strategies based on nucleophilic addition to serine derivatives such as Garner’s aldehyde,^17,18^ other serinals^19^ or a serine Weinreb amide^20^ seemed to be more attractive since serine is a cheap chiral starting material readily available in both d- and l-configurations. Since we^4^ and others^18,21^ experienced only low to moderate diastereoselectivities in addition reactions of various nucleophiles to Garner’s aldehyde, we were looking for an alternative approach. In this context, Lajoie’s orthoester-protected serine aldehydes **21a-c**(22) gained our attention (Scheme 2). Transformation of the carboxylic acid into its corresponding trioxabicyclo[2.2.2] ortho (OBO) ester installs a very bulky group enabling the attack of the nucleophile to the aldehyde moiety in the preferred orientation and at the same time keeping the oxidation state constant. The formed product possesses the *syn* (or *threo*) selectivity, which is fully in line with the nonchelation-controlled Felkin–Anh model. Consequently, Lajoie’s aldehyde has been successfully applied to the synthesis of several β-hydroxy,^23,24^ β-methoxy,^25^ and other unusual amino acids.^26^

In 2014, Karjalainen and Koskinen reported a scalable route based on Garner’s aldehyde **18** yielding *trans*-(2*R*,3*R*)-3-hydroxy pipecolic acid **15** in 11 steps with an overall yield of 31% and without the need for chromatographical purification of intermediates (A, Scheme 2).^27^ Our initial idea was to adapt the concept of a stereoselective addition of a metal alkyne species to Lajoie’s aldehyde **21c** combined with a stringent strategy of functionalization and ring closure to minimize the number of necessary steps. Since reductive amination is an established method of piperidine ring formation,^28^ we envisaged an addition reaction of an acetal-protected propiolaldehyde **20** to orthoester **21c** as the key step to deliver intermediate **19**, the precursor for the ring closure to **4**.^29^ After ring formation under reductive hydrogenation conditions, the free carboxylic acid **4** would be obtained directly after a hydrolysis-saponification sequence (B, Scheme 2) in contrast to the Garner’s aldehyde based route A which requires an additional oxidation step.

Furthermore, we planned to investigate serinals **21a** and **21b** as a potential substitute for Garner’s aldehyde **18** employed in the first total synthesis of GE81112A for the preparation of β-hydroxy histidine intermediate **22**. For this purpose, we chose the Fmoc- or Boc-protecting groups since hydrogenolytic Cbz removal would be incompatible with the chlorine present in the histidine moiety (C, Scheme 2).

## Results and Discussion

**Scheme 3:**
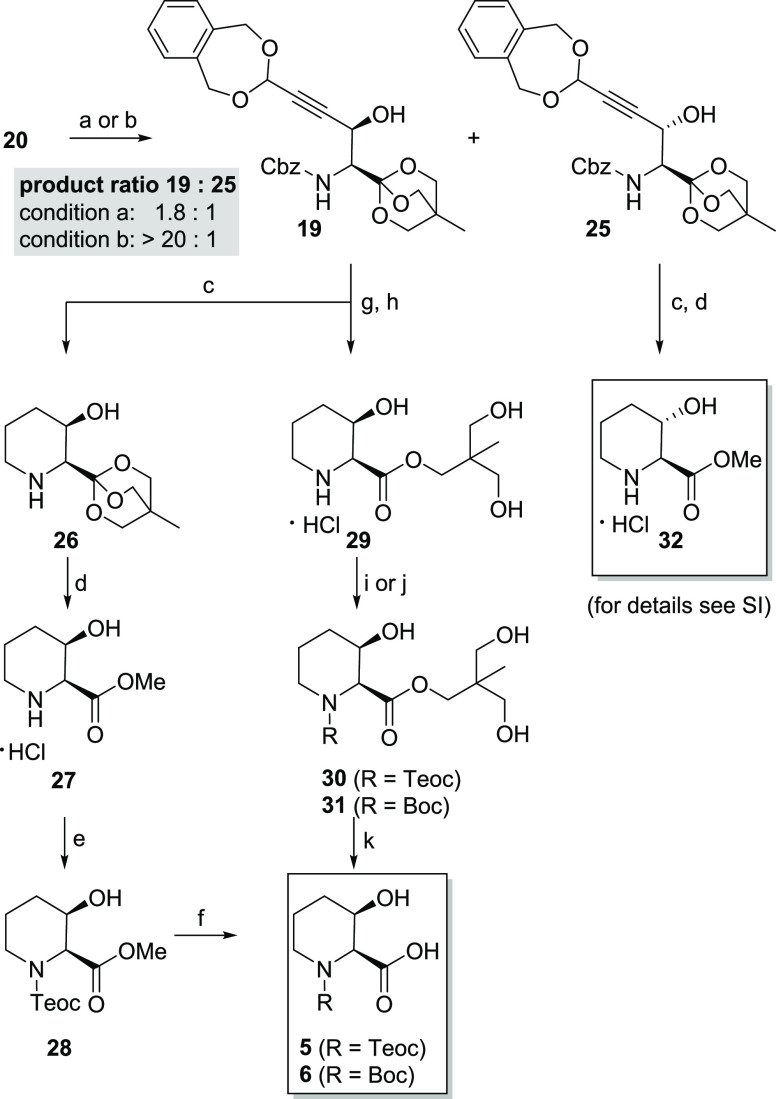
Stereoselective Alkynyl Addition and Synthesis of *cis*-3-Hydroxypipecolic Acid Derivatives **5** and **6** Conditions: (a) (i) **20** (4 equiv), EtMgBr (4 equiv), THF, rt, 13 h; (ii) **21c**, CH_2_Cl_2_/Et_2_O (1:1), −78 °C, 6.5 h, (**19**, 25% and **25**, 14%, *dr* 1.8:1); (b) (i) **20** (4 equiv), *n-*BuLi (4 equiv), THF, −78 °C, 10 min; rt, 10 min; (ii) **21c**, THF, −78 °C, 30 min, 81% (*dr* > 20:1); (c) H_2_, 85 bar), Pd(OH)_2_, THF, 70 °C; (d) AcCl, MeOH, 70 °C, 79% over two steps; (e) Teoc-OSu, NaHCO_3_, 1,4-dioxane/H_2_O (2:1), 0 °C to rt, 24 h, 42%; (f) 1 M LiOH_(aq.)_, THF, rt, 5 h, (**5**, 79%); (g) H_2_ (1 bar), 20 mol % Pd(OH)_2_, 10 mol % Pd/C, 20% AcOH_(aq.)_, 1,4-dioxane, rt, 24 h; (h) 1 M HCl_(aq.)_, 1,4-dioxane, rt, 2 h; (i) Teoc-OSu, NaHCO_3_, 1,4-dioxane/H_2_O (2:1), 0 °C to rt, 24 h, (**30**, 47% over three steps); (j) Boc_2_O, NaHCO_3_, 1,4-dioxane/H_2_O (2:1), 0 °C to rt, (**31**, 44% over three steps); (k) 1 M LiOH_(aq.)_, THF, rt, 6 h, (**5**, 95%), (**6**, 89%).

Starting from alkyne **20**,^30^ metalation with ethyl magnesium bromide followed by reaction with aldehyde **21c** at low temperature delivered products **19** and **25** with yields up to 39% and a diastereomeric ratio (*dr*) of merely 1.8:1 for the desired *syn* (*threo*) isomer **19** (Scheme 3).^31^ The low yield could be linked to an incomplete deprotonation of the terminal alkyne, which required extensive periods of time. In order to curb the long deprotonation periods, we substituted the Grignard species by a lithium organyl resulting in an increase of the yield to 81% and of the *dr* of >20:1 for the *syn*-product **19**. The reaction was upscaled to 2g resulting in a yield of 71% and a constant *ee* compared to utilizing aldehyde **21c** (*ee* = 97%). After recrystallization target, compound **19** was obtained in 60% yield and with an *ee* > 99%.

The conversion of intermediate **19** into literature-known *cis*-3-hydroxypipecolic acid methyl ester **27** was achieved by hydrogenation under high pressure (85 bar) in a Thales Nano H-Cube at 70 °C using Pearlman’s catalyst followed by OBO ester opening and transesterification under acidic conditions (Scheme 3). Reaction control (LC/MS) indicated that hydrogenation of the 1,5-dihydro-3*H*-2,4-benzodioxepin protecting group is the rate-limiting step which required a hydrogen pressure of 85 bar to obtain full conversion. Additionally, compound **27** was converted to target molecule **5** by Teoc-protection and saponification. Notably, the hydrogenation of **19** was possible under milder conditions (room temperature, atmospheric pressure) when diluted acetic acid was added. We hypothesized that protonation of the basic amine may prevent partial catalyst poisoning and facilitate hydrogenetic cleavage of the 1,5-dihydro-3*H*-2,4-benzodioxepin protecting group. The crude mixture was treated with aqueous hydrochloric acid to drive the orthoester hydrolysis to completion. The synthesis of the desired derivatives **5** and **6** was completed by carbamate protection of intermediate **29** and saponification (Scheme 3). Analogously, isomer **25** was successfully converted to the *trans*-3-hydroxypipecolic acid methyl ester **32**.^32^

**Scheme 4:**
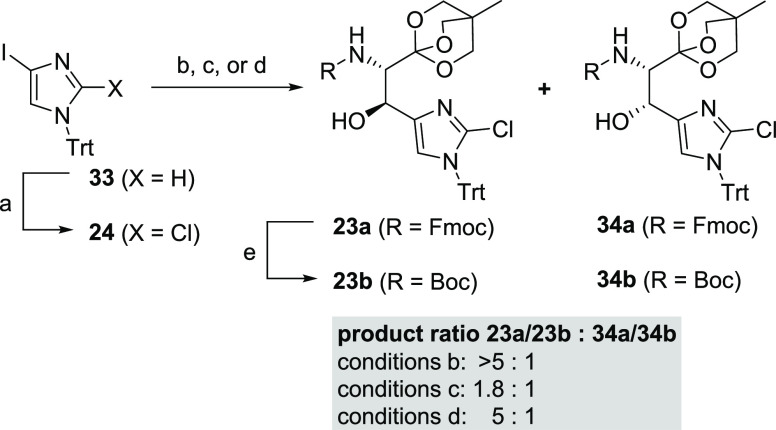
Addition of Metalated Imidazole **24** to Aldehydes **21a** and **21b** Conditions: (a) **33**, LDA (1.05 equiv), C_2_Cl_6_ (1.1 equiv), THF, −78 °C, 1 h, 86%; (b) (i) **24** (4.0 equiv), EtMgBr (4.0 equiv), CH_2_Cl_2_, 0 °C, 1 h; (ii) **21a**, CH_2_Cl_2_, −78 °C, 2 h, (67%, mixture **23a** and **34a**, ratio > 5:1); (c) (i) **24** (2.2 equiv), *i-*BuMgBr (2.2 equiv), CH_2_Cl_2_, 0 °C, 1 h; (ii) **21b**, CH_2_Cl_2_, −78 °C, 2 h, (83%, mixture **23b** and **34b**, ratio 1.8:1); (d) (i) **24** (3.0 equiv), *n-*BuLi (3.0 equiv), THF, −78 °C, 30 min; (ii) **21b**, THF, −78 °C, 5 h, (62% of pure **23b**) (e) (i) NHMe_2_, THF, rt; (ii) evaporation; (iii) Boc_2_O, THF, NaHCO_3(aq.)_, rt (65% of pure **23b**).

**Scheme 5:**
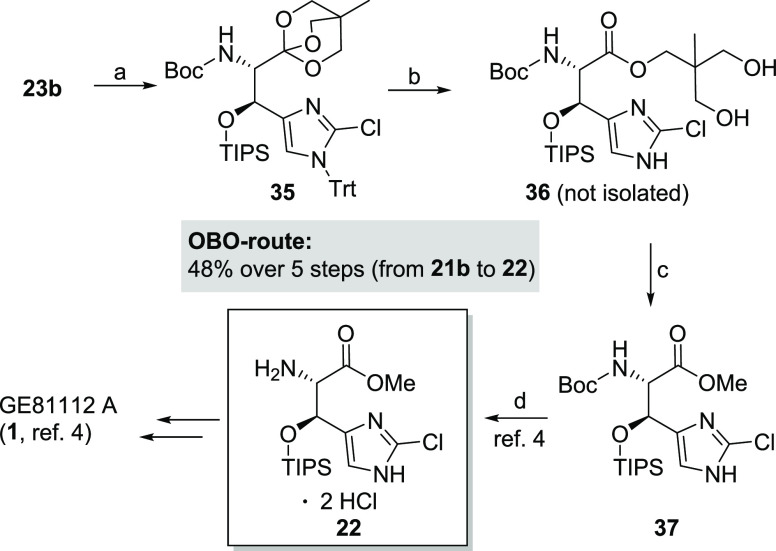
Synthesis of β-Hydroxy Histidine Building Block **22** and Formal Total Synthesis of GE81112A (1) Conditions: (a) TIPSOTf, 2,6-lutidine, CH_2_Cl_2_, −78 °C, 94%; (b) 80% AcOH_(aq.)_, TFE, 30 °C, 24 h; (c) MeOH, K_2_HPO_4_, 40 °C, 48 h, 83% over 2 steps; (d) 4 N HCl in 1,4-dioxane, THF, rt, 24 h, crude product **22** used without purification (ref (4)).

In comparison with the successfully established entry to the pipecolic acids **5** and **6**, an effective synthesis of β-hydroxy histidine intermediate **22** depended on the yield and stereoselectivity of the addition step of 2-chloro imidazole derivative **24** (prepared by lithiation of commercially available imidazole **33** and trapping of the lithium species with C_2_Cl_6_) to serinal **21a** (Scheme 4). Applying conditions similar to those of the previously described syntheses of alkyne addition products **19**/**25** gave access to desired stereoisomer **23a** and minor diastereomer **34a** as an inseparable mixture with a preparative yield of 67% and a synthetically useful selectivity (*dr* > 5:1). Since the separation of the diastereomers could not be achieved and test reactions demonstrated that the Fmoc-protecting group was not compatible to the basic transesterification conditions required for the synthesis of target compound **22** (Scheme 5), we switched to Boc as the protecting group. We were able to increase the yields while reducing the required equivalents of iodo-imidazole **24** which is irreversibly consumed by the iodine-magnesium exchange and cannot be recycled (in contrast to alkyne building block **20** that is metalated by deprotonation cf. Scheme 3). However, the selectivity dropped significantly (*dr* 1.8:1). Switching to the corresponding lithiated imidazole as a nucleophile retained the selectivity at a *dr* of 5:1. In contrast to the Fmoc-series, the isolation of pure diastereomer **23b** was possible by careful chromatography.^33^ To obtain **23b** in a preparative useful yield of 62% (after two chromatography runs), careful control of time, temperature, and equivalents of *n*-BuLi employed was required since the reaction turned out to be prone to decomposition when reaction conditions were modified. The remaining mixture of **23a**/**34a** was converted to **23b** and **34b** by a one-pot Fmoc-deprotection Boc-protection sequence, and the desired isomer **23b** was isolated as a pure compound after chromatography.

Intermediate **23b** was converted to TIPS-protected compound **35** followed by OBO ester opening and simultaneous trityl deprotection. The transesterification with K_2_HPO_4_ in methanol resulted in methyl ester **37** in good overall yield.^24^ Final Boc deprotection completed the synthesis of target compound **22** (Scheme 5). The synthesis of key intermediate **22** was thereby shortened, and the yields were increased from 15% over six steps to 48% over five steps. The sensitivity of OBO-protected intermediates **23b** and **35** toward *ortho*-ester hydrolysis was identified as the only disadvantage hampering chromatography, analytics, and storage of these intermediates.

**Scheme 6:**
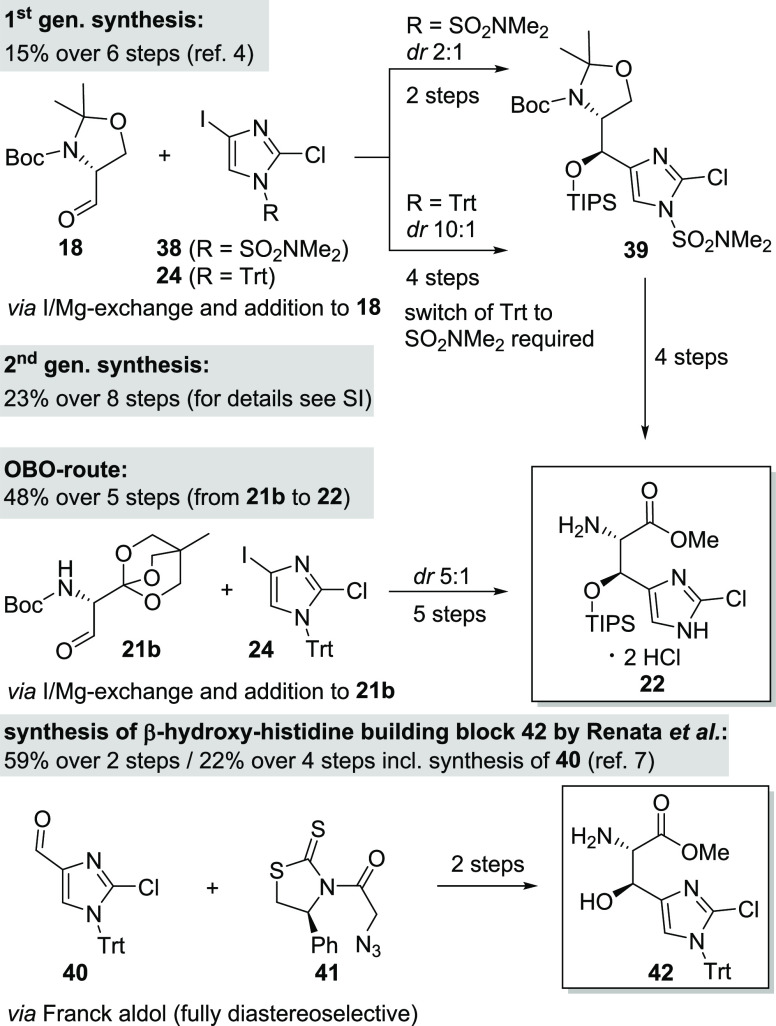
Comparison of Different Synthetic Routes toward β-Hydroxy Histidine Building Blocks **22** and **42**

Additionally, a second-generation synthesis based on the highly stereoselective addition (*dr* 10:1) of trityl-protected imidazole **24** to Garner’s aldehyde (**18**) was developed by us which gave a significant improvement in overall yield (cf. SI). The desired stereoisomer was obtained in high purity by crystallization on a 20 g scale. Unfortunately, even though the trityl group gave a higher selectivity, a change to the SO_2_NMe_2_ protection group was nonetheless required since it was more acid-tolerant. Thus, it was able to withstand the following acetonide cleavage and alcohol oxidation to the carboxylic acid **22** after initial TIPS protection. With these two improved synthetic routes toward intermediate **22** in hand, we not only achieved the formal total syntheses of GE81112A (**1**), but also secured a robust and scalable material supply of **22**, which is crucial for further SAR investigations. Compared to the remarkably short and fully diastereoselective synthetic access to β-hydroxy histidine intermediate **42** by Renata et al.^7^ (Scheme 6), our improved routes reached a similar level of convenience while relying solely on chemical means.

**Scheme 7:**
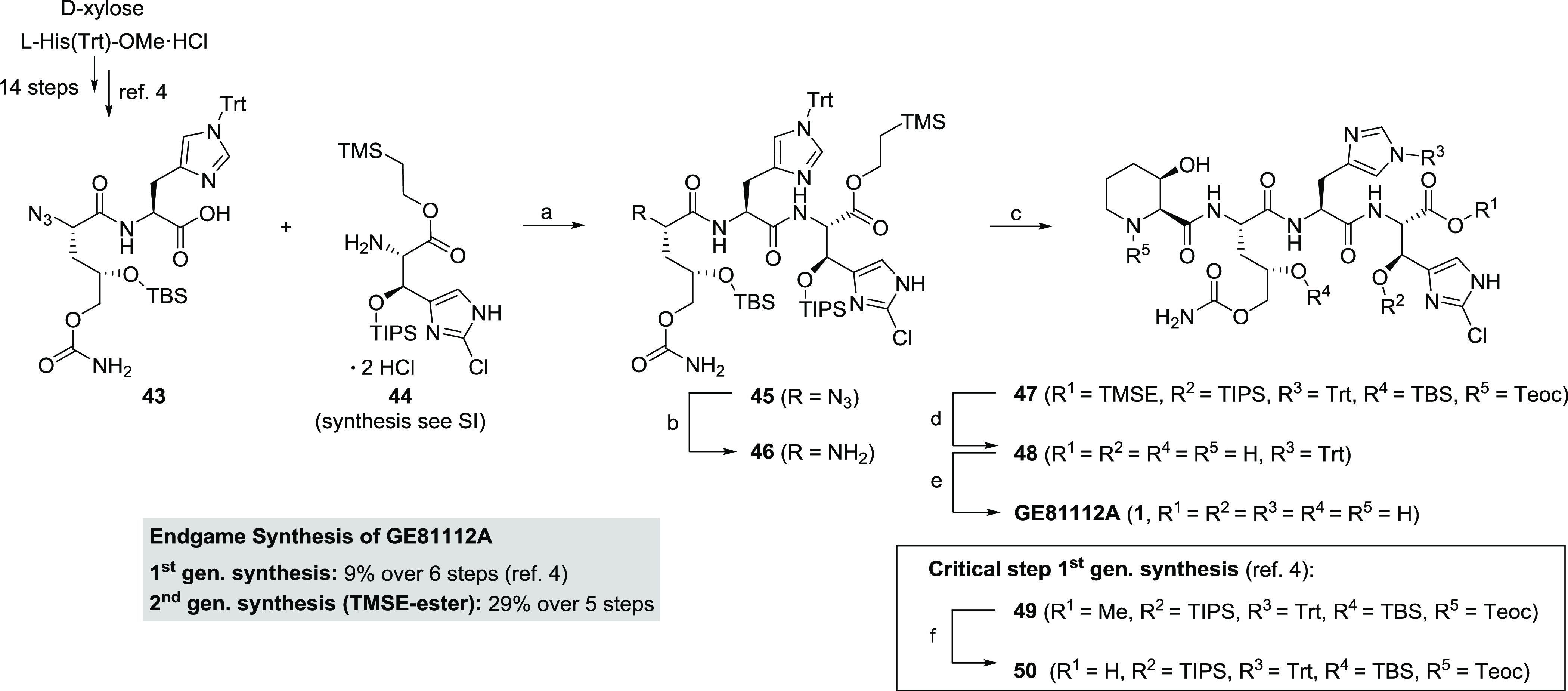
Synthesis of GE81112A (1) with a Modified Protecting Group Strategy Conditions: (a) EDC·HCl, Oxyma, NaHCO_3_, DMF/CH_2_Cl_2_ (1:1), −40 °C to rt, 48 h, 80%; (b) 1 M Me_3_P in THF, THF/H_2_O (5:1), rt, 12 h; (c) Acid **5**, EDC·HCl, Oxyma, NaHCO_3_, DMF/CH_2_Cl_2_ (1:1), 0 °C to rt, 12 h, 82% over 2 steps; (d) 1 M TAS-F in DMF, H_2_O, DMF, rt, 48 h, 67%; (e) 10% formic acid in TFE, rt, 12 h, 67%; (f) LiOH, THF/H_2_O, rt, 40% (ref (4)).

The most critical step within the endgame of our first-generation synthesis of GE81112A (**1**) was the saponification of methyl ester **49** (Scheme 7). Intermediates **49** and **50** were quite sensitive to basic conditions (resulting in partial decomposition). Only under well monitored conditions, a moderate yield of 42% for the saponification was achieved, and the reproducibility of that reaction—especially on scale—turned out to be difficult to control.^4^ These findings motivated us to modify the protection group strategy and to utilize TMSE-protected building block **44** (for details regarding the synthetic procedure cf. SI) for the endgame (Scheme 7). After coupling **44** with dipeptide **43** (derived in 14 steps from d-xylose and l-His(Trt)-OMe) to **45**, the reduced amine **46** could be coupled with building block **5**, derived now from the OBO route. With the TMSE ester in place on the C-terminus, the separate saponification step could be omitted, and all fluoride labile protecting groups (TMSE, TIPS, TBS, and Teoc) of protected tetrapeptide **47** were removed in one step by TAS-F under mild and neutral conditions. Thus, compound **48** could be isolated in an acceptable yield.^34^ After final trityl deprotection, GE81112A (**1**) was obtained in 67% yield. Overall, this modification shortened the endgame by one synthetic step, facilitated chromatographic purification due to less side product formation, and improved the yield significantly from 9 to 29% over five steps.

## Conclusions

We have demonstrated that Lajoie’s OBO-protected serinals **21a-c** are readily available and synthetically useful building blocks for the synthesis of β-hydroxy amino acids in *threo* configuration. Compared to the widely used Garner’s aldehyde **18**, the high *threo* selectivity caused by the steric bulk of the OBO-orthoester (in line with the nonchelation-controlled Felkin–Anh model) and no need for a final oxidation of the addition product to the carboxylic acid are prime advantages of the corresponding routes over the established ones. In the synthesis of *cis*-3-hydroxy pipecolic acids **5** and **6**, a concise and selective access was developed, which was in our hands more convenient than other literature-known strategies. Furthermore, Boc-protected serinal **21b** enabled us to improve our first-generation synthesis of the β-hydroxy histidine building block **22** significantly. As main drawbacks, the tendency of aldehydes **21a-c** to epimerize and the sensitivity of the OBO-orthoester protecting group toward hydrolysis were identified, which require increased care during experimentation and precautionary measures and may be a reason why the use of **21a-c** remained a niche in the synthesis of β-hydroxy amino acids compared to Garner’s aldehyde (**18**). Nevertheless, our synthetic studies enabled us to improve the sophisticated total synthesis of GE81112A (**1**) allowing us to provide the building blocks for synthetic analogues of the GE-series. Since key questions concerning the biological activity and the pharmacological and safety profile of GE81112 remain to be adressed,^5^ modification of the structural backbone and therefore synthetical access is of utmost importance to answer these. The reported results helped us to tackle a number of these issues by generating a number of new derivatives which will be reported in due course.

## Experimental Section

### General Information

All chemicals and solvents/anhydrous solvents were commercially supplied and used without further purification. For heating of reaction mixtures, aluminum flask carriers in different sizes from IKA were used. Reactions were monitored using thin layer chromatography (TLC) or using one of the following LCMS systems: 1100 HPLC (Agilent) with DAD equipped with an Esquire 3000plus MS detector (Bruker) or 1200 HPLC (Agilent) with DAD equipped with MSD (Agilent) ESI quadrupole MS. TLC was performed on precoated silica gel glass plates (Merck TLC Silica gel 60 F254), and compounds were detected under UV light (254 nm) and/or by staining with an aqueous solution of KMnO_4_ with K_2_CO_3_ and NaOH, or an aqueous solution of phosphomolybdic acid, cerium(IV) sulfate, and H_2_SO_4_, or a solution of ninhydrin in *n*-BuOH/AcOH (100:3) followed by heating with a heat gun. Products were purified by flash column chromatography using silica gel 60 M (Macherey-Nagel) or silica gel from Merck (particle size: 40–63 μm, 60 Å average diameter) or by using automated flash column chromatography systems (puriFlash XS 520Plus from Interchim or Reverlis PREP device from Büchi) equipped with ISOLUTE Flash SI II columns of different sizes from Biotage or PF-15SIHC flash columns of different sizes from Interchim (eluants are given in parentheses). For all OBO-orthoester-containing compounds, conditioning with 2% NEt_3_ in *n*-heptane prevented OBO-orthoester hydrolysis by the slightly acidic silica. Reverse-phase flash column chromatography was performed on a FlashPure EcoFlex C18 (Bruker) equipped with FlashPure EcoFlex C18 end capped columns (eluants are given in parentheses). Preparative HPLC was performed on a WATERS AutoPurification HPLC/MS system equipped with a WATERS Sunfire Prep C18 OBD 5 μm 50 × 100 mm column with water/TFA 0.1% as mobile phase A and acetonitrile as mobile phase B (eluents are given in parentheses). The product-containing fractions were collected and freeze-dried to yield the final product. NMR spectra were recorded on a Bruker AVANCE II spectrometer (400 MHz), a Bruker AVANCE III spectrometer (600 MHz), a Bruker AMX400 spectrometer (400 MHz), or a Bruker DPX400 spectrometer (400 MHz) with CDCl_3_, C_6_D_6_, DMDO-d_6,_ or D_2_O as the solvent with chemical shifts (δ) quoted in parts per million (ppm) and referenced to the solvent signal (δ^1^H/^13^C: CDCl_3_ 7.26/77.2, C_6_D_6_ 7.16/128.1, DMDO-d_6_ 2.50/39.5). For all OBO-orthoester-containing compounds, CDCl_3_ was filtered through a small plug of basic Al_2_O_3_ prior to sample preparation in order to prevent OBO-orthoester hydrolysis by traces of acid. Assignment was confirmed based on COSY, HSQC, HMBC, and NOESY correlations. High-resolution mass spectrometry was performed on a 1290 UPLC (Agilent) with DAD and ELSD equipped with maXis II (Bruker) ESI TOF MS or Alliance 2695 (Waters) with DAD equipped with a Micromass LCT device (Waters) or an Acquity UPLC (Waters) with DAD equipped with a Micromass Q TOF Premier mass spectrometer (Waters). Specific rotation was measured by a polarimeter (MCP 100 polarimeter) from Anton Paar or a polarimeter (P 3000 series) from Krüss. Some hydrogenations were performed with an H-Cube Mini Plus from Thales Nano with a flow rate of 0.3 mL/min.

#### Benzyl ((1*S*,2*R*)-4-(1,5-Dihydrobenzo[*e*][1,3]dioxepin-3-yl)-2-hydroxy-1-(4-methyl-2,6,7-trioxabicyclo[2.2.2]octan-1-yl)but-3-yn-1-yl)carbamate (**19**) and Benzyl ((1*S*,2*S*)-4-(1,5-Dihydrobenzo[*e*][1,3]dioxepin-3-yl)-2-hydroxy-1-(4-methyl-2,6,7-trioxabicyclo[2.2.2]octan-1-yl)but-3-yn-1-yl)carbamate (**25**)

##### Condition 1 (Deprotonation with EtMgBr; Scheme 3, Condition a)

Alkyne **20** (5.77 g, 33.1 mmol, 4.00 equiv) was dissolved in THF (40 mL). EtMgBr (0.9 M in *tert*-butylmethylether, 36.8 mL, 33.1 mmol, 4.00 equiv) was added dropwise at room temperature and stirred for 1 h. The reaction mixture was then cooled to −78 °C. Aldehyde **21c** (2.69 g, 8.21 mmol, 1.00 equiv) was dissolved in Et_2_O/CH_2_Cl_2_ (30 mL, 1:1) in a separate flask, cooled to −78 °C, and then added dropwise to the alkyne-solution. The reaction mixture was stirred at −78 °C for 6.5 h and was then diluted with saturated aqueous NH_4_Cl (40 mL) and CH_2_Cl_2_ (100 mL). The layers were separated, and the aqueous layer was re-extracted with CH_2_Cl_2_ (3 × 25 mL). The combined organic layers were washed with saturated aqueous NH_4_Cl (20 mL), H_2_O (20 mL), and saturated aqueous NaCl (20 mL) and were dried over MgSO_4_ and filtered, and the solvent was removed under reduced pressure. The crude product was purified via column chromatography (silica, conditioning with 2% NEt_3_ in *n*-heptane, 0–100% ethyl acetate in *n*-heptane) to obtain the separated diastereomers **19** (1.03 g, 2.07 mmol, 25%) and **25** (562 mg, 1.13 mmol, 14%) as colorless solids.

##### Condition 2 (Deprotonation with *n*-BuLi, Scheme 3, Condition b)

Alkyne **20** (0.79 g, 4.5 mmol, 4.0 equiv) was dissolved in THF (15 mL) and then cooled to −78 °C. Then *n*-BuLi (2.5 M in *n-*hexane, 1.8 mL, 4.5 mmol, 4.0 equiv) was added dropwise, and the reaction mixture was stirred for 10 min. The reaction mixture was then stirred without a cooling bath for 10 min and afterward cooled to −78 °C again. Aldehyde **21c** (0.36 g, 1.1 mmol, 1.0 equiv) was dissolved in THF (10 mL) in a separate flask, cooled to −78 °C, and then added dropwise to the alkyne-solution. The reaction mixture was stirred at −78 °C for 0.5 h and was then diluted with saturated aqueous NH_4_Cl (25 mL) and ethyl acetate (80 mL). The layers were separated, and the organic layer was washed with saturated aqueous NaHCO_3_ (20 mL) and saturated aqueous NaCl (30 mL) and was dried over MgSO_4_ and filtered, and the solvent was removed under reduced pressure. The crude product was purified via column chromatography (silica, conditioning with 2% NEt_3_ in *n*-heptane, 0–100% ethyl acetate in *n*-heptane) to obtain the diastereomeric mixture of **19** and **25** (0.45 g, 0.91 mmol, 81%, *dr* > 20:1; *dr* determined by ^1^H-NMR) as a colorless solid.

To gain access to the pure diastereomer **19**, the reaction was repeated in a bigger scale and under the same reaction conditions: Starting from alkyne **20** (4.75 g, 27.3 mmol, 4.00 equiv) and aldehyde **21c** (2.19 g, 6.82 mmol, 1.00 equiv), the diastereomeric mixture of **19** and **25** (2.40 g, 4.85 mmol, 71%, *dr* > 20:1; *dr* determined by ^1^H-NMR) was recrystallized from *n*-heptane/ethyl acetate (10:1) to obtain pure diastereomer **19** (1.81 g, 3.66 mmol, 54%, *ee* > 99%) as a colorless solid. Major diastereomer **19**: ^1^H-NMR (CDCl_3_, 600 MHz): 7.335 (bs, 2H, Cbz-aryl-*H*, 7.332 (bs, 2H, Cbz-aryl-*H*), 7.29 (bs, 1H, Cbz-aryl-*H*), 7.20–7.17 (m, 2H, Aryl-*H*), 7.09–7.07 (m, 2H, Aryl-*H*), 5.58 (s, 1H, C*H*-OCH_2_), 5.47 (d*, J* = 10.2 Hz, 1H, N*H*), 5.12 (d, *J* = 14.4 Hz, 1H, CH-OC*H_2_*), 5.10 (d, *J* = 14.4 Hz, 1H, C*H*-OCH_2_), 5.09 (bs, 2H, Cbz-C*H_2_*), 4.98 (bs, 1H, C*H*-OH), 4.73 (d, *J* = 14.4 Hz, 1H, CH-OC*H_2_*), 4.71 (d, *J* = 14.4 Hz, 1H, CH-OC*H_2_*), 4.17 (dd, *J* = 10.2 Hz, 1.5 Hz, 1H, C_α_*H*), 3.93 (s, 6H, OBO-C*H_2_*), 3.16 (d, *J* = 3.1 Hz, 1H, O*H*), 0.82 (s, 3H, C*H_3_*); ^13^C{1H}-NMR (CDCl_3_, 150 MHz): 156.6 (s, *C*=O), 138.42 (s, Ar-*C_q_*), 138.40 (s, Ar-*C_q_*), 136.6 (s, Cbz-Ar-*C_q_*), 128.6 (d, Cbz-Ar-*C*H), 128.16 (d, Cbz-Ar-*C*H), 128.13 (d, Cbz-Ar-*C*H), 127.16 (d, Ar-*C*H), 126.89 (d, Ar-*C*H), 126.9 (d, Ar-*C*H), 126.9 (d, Ar-*C*H), 108.4 (s, OBO-*C_q_*), 93.3 (d, *C*H-OCH_2_), 83.1 (s, HO-CH-*C*≡C), 80.5 (s, HO-CH-C≡*C*), 72.9 (t, OBO-*C*H_2_), 68.8 (t, *C*H_2_-OCH), 68.7 (t, *C*H_2_-OCH), 67.1 (t, Cbz-*C*H_2_), 62.0 (d, *C*H-OH), 57.6 (d, *C*_α_H), 30.8 (s, OBO-*C_q_*), 14.4 (q, *C*H_3_); HRMS (ESI) *m*/*z*: [M + H]^+^ calcd for C_27_H_30_NO_8_ 496.1966; found: 496.1971; *R*_f_ (*n*-heptane/ethyl acetate 3:1): 0.48; specific rotation [α]_D_^20^ = −7.5° (*c* = 0.03; CH_2_Cl_2_); HPLC (Chiralcel OJ-H, EtOH/MeOH = 50/50, flow rate = 1.0 mL/min, l = 210 nm) *t*_R_ = 5.9 min (**19**). Minor diastereomer **25**: ^1^H-NMR (CDCl_3_, 600 MHz): 7.33 (bs, 2H, Cbz-aryl-*H*), 7.33 (bs, 2H, Cbz-aryl-*H*), 7.31 (bs, 1H, Cbz-aryl-*H*), 7.20–7.17 (m, 2H, Aryl-*H*), 7.10–7.06 (m, 2H, Aryl-*H*), 5.60 (s, 1H, C*H*-OCH_2_), 5.30 (d, *J* = 9.2 Hz, 1H, N*H*), 5.14 (d, *J* = 14.4 Hz, 1H, C*H*-OCH_2_), 5.11 (d, *J* = 14.4 Hz, 1H, C*H*-OCH_2_), 5.09–5.06 (m, 2H, Cbz-C*H_2_*), 4.78 (dd, *J* = 6.7 Hz, 4.8 Hz, 1H, C*H*-OH), 4.75 (d, *J* = 14.4 Hz, 1H, CH-OC*H_2_*), 4.73 (d, *J* = 14.4 Hz, 1H, CH-OC*H_2_*), 4.13 (dd, *J* = 9.2 Hz, 4.8 Hz, 1H, C_α_*H*), 3.91 (s, 6H, OBO-C*H_2_*), 3.64 (d, *J* = 6.8 Hz, 1H, O*H*), 0.81 (s, 3H, C*H_3_*); ^13^C{1H}-NMR (CDCl_3_, 150 MHz): 157.1 (s, *C*=O), 138.5 (s, Ar-*C_q_*), 138.4 (s, Ar-*C_q_*), 136.3 (s, Cbz-Ar-*C_q_*), 128.6 (d, Cbz-Ar-*C*H), 128.3 (d, Cbz-Ar-*C*H), 128.3 (d, Cbz-Ar-*C*H), 127.2 (d, Ar-*C*H), 127.2 (d, Ar-*C*H), 126.9 (d, Ar-*C*H), 107.7 (s, OBO-*C_q_*), 93.5 (d, *C*H-OCH_2_), 83.5 (s, HO-CH-*C*≡C), 81.3 (s, HO-CH-C≡*C*), 72.9 (t, OBO-*C*H_2_), 68.7 (t, *C*H_2_-OCH), 68.7 (t, *C*H_2_-OCH), 67.4 (t, Cbz-*C*H_2_), 63.4 (d, *C*H-OH), 59.3 (d, *C*_α_H), 30.8 (s, OBO-*C_q_*), 14.4 (q, *C*H_3_); HRMS (ESI) *m*/*z*: [M + H]^+^ calcd for C_27_H_30_NO_8_ 496.1966; found: 496.1971; *R*_f_ (*n*-heptane/ethyl acetate 3:1): 0.6; HPLC (Chiralcel OJ-H, EtOH/MeOH = 50/50, flow rate = 1.0 mL/min, l = 210 nm) *t*_R_ = 11.1 min (**25**).^35^

#### (2*S*,3*R*)-2-(4-Methyl-2,6,7-trioxabicyclo[2.2.2]octan-1-yl)piperidin-3-ol (**26**)

Alkyne **19** (70 mg, 0.14 mmol, 1.0 equiv) was dissolved in THF (28 mL). The reaction mixture was hydrogenated by the H-Cube at 70 °C and 85 bar in the presence of Pd(OH)_2_ (20 mol %). After completion of the reaction, the solvent was removed under reduced pressure. Amine **26** (32 mg, 0.14 mmol, quant.) was obtained as a yellowish oil. The acid-labile and highly polar crude product was used without further purification and analytics. HRMS (ESI) *m*/*z*: [M + H]^+^ calcd for C_11_H_20_NO_4_: 230.1387; found: 230.1396.

#### (2*S*,3*R*)-3-Hydroxy-2-(methoxycarbonyl)piperidine Hydrochloride (**27**)

Dry MeOH (2.3 mL) was cooled to 0 °C. Acetyl chloride (1 mL) was added dropwise, and the reaction mixture was stirred at room temperature for 20 min. Afterward, OBO ester **26** (32 mg, 0.14 mmol, 1.0 equiv) was dissolved in MeOH (1 mL) and added dropwise. The reaction mixture was heated to 70 °C and was stirred for 48 h. The solvent was removed under reduced pressure, and the crude product was applied to cation exchange chromatography (Dowex 50WX8 200–400, MeOH) in order to obtain methylester hydrochloride **27** (39 mg; 0.20 mmol; 79% over 2 steps) as a colorless oil. ^1^H-NMR (D_2_O, 400 MHz): 4.57 (m, 1H, C*H*-OH), 4.18 (m, 1H, C_α_*H*), 3.85 (s, 3H, C*H_3_*), 3.47 (m, 1H, C*H_2_*-NH), 3.05 (m, 1H, C*H_2_*-NH), 1.98 (m, 2H, C*H_2_*-OH), 1.81 (m, 2H, C*H_2_*-CH_2_-NH); ^13^C{1H}-NMR (D_2_O, 100 MHz): 168.7 (s, *C*=O), 63.6 (d, *C*H-OH), 60.5 (d, *C*_α_), 53.6 (q, *C*H_3_), 43.7 (t, *C*H_2_-NH), 28.0 (t, *C*H_2_-CH-OH), 15.6 (t, *C*H_2_-CH_2_-NH); HRMS (ESI) *m*/*z*: [M + H]^+^ calcd for C_7_H_14_NO_3_ 160.0968; found: 160.0967; *R*_f_ (15% MeOH in CH_2_Cl_2_): 0.3; specific rotation [α]_D_^25^ = −15.88° (*c* = 0.6; H_2_O).

#### 2-Methyl 1-(2-(Trimethylsilyl)ethyl) (2*R*,3*S*)-3-Hydroxypiperidine-1,2-dicarboxylate (**28**)

Amine **27** (30 mg, 0.19 mmol, 1.0 equiv) was dissolved in 1,4-dioxane/H_2_O (2:1, 6 mL). The reaction mixture was cooled to 0 °C. Solid NaHCO_3_ (475 mg, 5.60 mmol, 30.0 equiv) and water (3 mL) were added to adjust a pH of 9. Afterward, Teoc-OSu (49 mg, 0.19 mmol, 1.0 equiv) was dissolved in 1,4-dioxane (1 mL) and added dropwise. The reaction mixture was stirred at room temperature overnight. The reaction mixture was extracted with ethyl acetate (3 × 15 mL). The combined organic layers were dried over Na_2_SO_4_ and filtered. The solvent was removed under reduced pressure, and the crude product was purified via column chromatography (silica, 1% MeOH in CH_2_Cl_2_) to obtain carbamate **28** (24 mg, 0.080 mmol, 42%) as a yellowish oil. ^1^H-NMR (CDCl_3_, 400 MHz): 5.17–5.06 (m, 1H, C_α_*H*), 4.26–4.15 (m, 2H, TMS-CH_2_-C*H_2_*), 4.10–3.86 (m, 1H, C*H_2_*-NH), 3.77 (s, 3H, C*H_3_*), 3.78–3.69 (m, 1H, C*H*-OH), 2.81–2.58 (m, 1H, C*H_2_*-NH), 2.07–1.93 (m, 1H, C*H_2_*-CH-OH), 1.76–1.64 (m, 1H, C*H_2_*-CH_2_-NH), 1.56–1.43 (m, 2H, C*H_2_*-CH_2_-NH, C*H_2_*-CH-OH), 1.05–0.92 (m, 2H, C*H_2_*-TMS), 0.03 (s, 9H, TMS); ^13^C{1H}-NMR (CDCl_3_, 100 MHz): 172.4 (s, CH_3_O-*C*=O), 156.4 (s, N-*C*=O), 68.9 (d, *C*H-OH), 64.5 (t, TMS-CH_2_-*C*H_2_), 57.7 (d, *C*_α_), 52.5 (q, *C*H_3_), 41.1 (t, *C*H_2_-N), 30.4 (t, *C*H_2_-CH-OH), 23.9 (t, *C*H_2_-CH_2_-N), 17.8 (t, TMS-*C*H_2_), −1.4 (q, TMS); HRMS (ESI) *m*/*z*: [M + Na]^+^ calcd for C_13_H_25_NO_5_SiNa 326.1394; found: 326.1399; *R*_f_ (5% MeOH in CH_2_Cl_2_): 0.56; specific rotation [α]_D_^25^ = −23.11° (*c* = 1.2; CHCl_3_).

#### 3-Hydroxy-2-(hydroxymethyl)-2-methylpropyl (2*S*,3*R*)-3-Hydroxypiperidine-2-carboxylate Hydrochloride (**29**)

Alkyne **19** (155 mg, 0.313 mmol, 1.00 equiv) was dissolved in 1,4-dioxane (10 mL). Pd(OH)_2_ (20 mol %, 220 mg, 0.313 mmol, 1.00 equiv) and Pd/C (10 mol %, 333 mg, 0.313 mmol, 1.00 equiv) were added. Aqueous AcOH (20%, 0,5 mL) was added, and the reaction mixture was hydrogenated at room temperature under a hydrogen atmosphere (1 bar). After completion of the reaction, the reaction mixture was filtered by Celite, and the solvent was removed under reduced pressure. The crude product was dissolved in 1,4-dioxane (2 mL), and aqueous HCl (1 M, 0.300 mL, 0.391 mmol, 1.25 equiv) was added. The reaction mixture was stirred for 2 h at room temperature and then neutralized by solid NaHCO_3_. The reaction mixture was extracted with ethyl acetate (3 × 30 mL). The combined organic layers were dried over Na_2_SO_4_ and filtered. The solvent was removed under reduced pressure to obtain amine **29** (32 mg, 0.14 mmol, quant.) as colorless oil. The highly polar crude product was utilized in the next step without further purification. HRMS (ESI) *m*/*z*: [M + H]^+^ for C_11_H_22_NO_5_ 248.1492; found: 248.1501.

#### 2-(3-Hydroxy-2-(hydroxymethyl)-2-methylpropyl) 1-(2-(Trimethylsilyl)ethyl) (2*S*,3*R*)-3-Hydroxypiperidine-1,2-dicarboxylate (**30**)

Amine **29** (19 mg, 0.077 mmol, 1.0 equiv) was carbamate protected analogously to **28**. The resulting crude product was purified via column chromatography (silica, 100% ethyl acetate) to obtain Teoc-protected 3-hydroxypipecolic ester **30** (14 mg, 0.033 mmol, 47%) as yellowish oil. ^1^H-NMR (CDCl_3_, 400 MHz): 5.03–4.96 (m, 1H, C_α_*H*), 4.31–4.24 (m, 1H, CO_2_-C*H_2_*), 4.24–4.14 (m, 2H, TMS-CH_2_-C*H_2_*), 4.14–4.08 (m, 1H, CO_2_-C*H_2_*), 3.94–3.84 (m, 1H, C*H_2_*-N), 3.85–3.74 (m, 1H, C*H*-OH), 3.59–3.54 (m, 4H, C*H_2_*-OH), 3.00 (t, *J* = 11.2 Hz, 1H, C*H_2_*-N), 2.00–1.89 (m, 1H, C*H_2_*-CH_2_-N), 1.78–1.69 (m, 1H, C*H_2_*-CH_2_-N), 1.59–1.47 (m, 2H, C*H_2_*-CH-OH), 0.99 (t, *J* = 8.3 Hz, 2H, TMS-C*H_2_*), 0.83 (s, 3H, C*H_3_*), 0.03 (s, 9H, TMS); ^13^C{1H}-NMR (CDCl_3_, 100 MHz): 171.8 (s, C_α_H-*C*=O), 156.9 (s, N-*C*=O), 68.6 (d, *C*H-OH), 68.2 (t, C_α_H-CO_2_-*C*H_2_), 67.4 (t, *C*H_2_-OH), 67.1 (t, *C*H_2_-OH), 64.6 (t, TMS-CH_2_-*C*H_2_), 58.3 (d, *C*_α_), 41.2 (s, *C_q_*), 40.6 (t, *C*H_2_-N), 29.7 (t, *C*H_2_-CH-OH), 23.4 (t, *C*H_2_-CH_2_-N), 17.8 (t, TMS-*C*H_2_), 17.2 (q, *C*H_3_), −1.4 (q, TMS); HRMS (ESI) *m*/*z*: [M + Na]^+^ calcd for C_17_H_33_NO_7_SiNa 414.1919; found: 414.1925; *R*_f_ (ethyl acetate): 0.3; specific rotation [α]_D_^20^ = −33.4° (*c* = 1.0; CHCl_3_).

#### 1-(*tert*-Butyl) 2-(3-Hydroxy-2-(hydroxymethyl)-2-methylpropyl) (2*S*,3*R*)-3-Hydroxypiperidine-1,2-dicarboxylate (**31**)

Amine **29** (77 mg, 0.31 mmol, 1.0 equiv) was dissolved in 1,4-dioxane/H_2_O (3 mL, 2:1). The reaction mixture was cooled to 0 °C. Solid NaHCO_3_ (789 mg, 9.40 mmol, 30.0 equiv) and water (3 mL) were added to adjust a pH of 9. Afterward, Boc_2_O (68 mg, 0.31 mmol, 1.0 equiv) was dissolved in 1,4-dioxane (1 mL) and was added dropwise at 0 °C. The reaction mixture was stirred at room temperature overnight. The reaction mixture was extracted with ethyl acetate (3 × 10 mL), the combined organic layers were dried over Na_2_SO_4_ and filtered, and the solvent was removed under reduced pressure. The resulting crude product was purified via column chromatography (silica, 100% ethyl acetate) to obtain Boc-protected 3-hydroxypipecolic ester **31** (48 mg, 0.14 mmol, 44%) as a yellowish oil. Main conformer: ^1^H-NMR (CDCl_3_, 400 MHz): 4.96–4.93 (m, 1H, C_α_*H*), 4.24 (s, 1H, CO_2_-C*H_2_*), 4.17–4.04 (m, 1H, CO_2_-C*H_2_*), 4.02–3.84 (m, 1H, C*H_2_*-N), 3.81–3.76 (m, 1H, C*H*-OH), 3.59–3.48 (m, 4H, C*H_2_*-OH), 2.99–2.86 (m, 1H, C*H_2_*-N), 1.98–1.87 (m, 1H, C*H_2_*-CH-OH), 1.77–1.65 (m, 1H, C*H_2_*-CH_2_-N), 1.55–1.47 (m, 2H, C*H_2_*-CH_2_-N, C*H_2_*-CH-OH), 1.44 (s, 9H, Boc), 0.83 (s, 3H, C*H_3_*); ^13^C{1H}-NMR (CDCl_3_, 100 MHz): 172.0 (s, C_α_H-*C*=O), 154.9 (s, Boc-*C*=O), 81.0 (s, Boc-*C_q_*), 72.9 (d, *C*H-OH), 68.1 (t, *C*H_2_-OH), 66.9 (t, CO_2_-*C*H_2_), 57.8 (d, *C*_α_), 41.4 (s, CH_3_-*C_q_*), 40.6 (t, *C*H_2_-N), 29.7 (t, *C*H_2_-CH-OH), 28.4 (q, Boc), 23.5 (t, *C*H_2_-CH_2_-N), 17.2 (q, *C*H_3_). Minor conformer: ^1^H-NMR (CDCl_3_, 400 MHz): 4.93–4.86 (m, 1H, C_α_*H*), 4.27 (s, 1H, CO_2_-C*H_2_*), 4.17–4.04 (m, 1H, CO_2_-C*H_2_*), 4.03–3.91 (m, 1H, C*H_2_*-N), 3.82–3.75 (m, 1H, C*H*-OH), 3.59–3.48 (m, 4H, C*H_2_*-OH), 2.84–2.70 (m, 1H, C*H_2_*-N), 1.98–1.87 (m, 1H, C*H_2_*-CH-OH), 1.77–1.65 (m, 1H, C*H_2_*-CH_2_-N), 1.55–1.47 (m, 2H, C*H_2_*-CH_2_-N, C*H_2_*-CH-OH), 1.44 (s, 9H, Boc), 0.83 (s, 3H, C*H_3_*); ^13^C{1H}-NMR (CDCl_3_, 100 MHz): 172.0 (s, C_α_H-*C*=O), 154.9 (s, Boc-*C*=O), 81.0 (s, Boc-*C_q_*), 72.9 (d, *C*H-OH), 68.6 (t, *C*H_2_-OH), 67.1 (t, CO_2_-*C*H_2_), 57.8 (d, *C*_α_), 41.4 (s, CH_3_-*C_q_*), 40.6 (t, *C*H_2_-N), 29.7 (t, *C*H_2_-CH-OH), 28.4 (q, Boc), 23.5 (t, *C*H_2_-CH_2_-N), 17.2 (q, *C*H_3_); HRMS (ESI) *m*/*z*: [M + Na]^+^ calcd for C_16_H_29_NO_7_Na, 370.1836; found: 370.1839; *R*_f_ (ethyl acetate): 0.25; specific rotation [α]_D_^20^ = −35° (*c* = 1.0; CHCl_3_).

#### (2*S*,3*R*)-3-Hydroxy-1-((2-(trimethylsilyl)ethoxy)carbonyl)piperidine-2-carboxylic Acid (**5**)

From intermediate **28**. Ester **28** (24 mg, 0.079 mmol, 1.0 equiv) was dissolved in THF (0.8 mL). Aqueous LiOH (1 M, 0.079 mL, 0.079 mmol, 1.0 equiv) was added, and the reaction mixture was stirred for 5 h at room temperature. The reaction mixture was neutralized by addition of AcOH (4.5 μL, 0.079 mmol, 1.0 equiv), and the solvent was removed under reduced pressure. The crude product was purified *via* MPLC (RP-C18, 4 g, 20 min, 0.05% TFA in CH_3_CN/H_2_O, 50–60% CH_3_CN) to obtain Teoc-protected 3-hydroxypipecolic acid **5** (18 mg, 0.062 mmol, 79%) as colorless oil.

From intermediate **30**: Ester **30** (780 mg, 1.90 mmol, 1.00 equiv) was dissolved in THF (20 mL). Aqueous LiOH (1 M, 2.79 mL, 2.79 mmol, 1.40 equiv) was added, and the reaction mixture was stirred for 6 h at room temperature. The reaction mixture was then neutralized by addition of AcOH (0.160 mL, 2.79 mmol, 1.40 equiv), and the solvent was removed under reduced pressure. The crude product was purified *via* MPLC (RP-C18, 12 g, 20 min, 0.05% TFA in CH_3_CN/H_2_O, 50–60% CH_3_CN) to obtain Teoc-protected 3-hydroxypipecolic acid **5** (550 mg, 1.90 mmol, 95%) as colorless oil. ^1^H-NMR (C_6_D_6_, 400 MHz): 5.40–5.21 (m, 1H, C_α_*H*), 4.22 (t, *J* = 7.7 Hz, 2H, TMS-CH_2_-C*H_2_*), 3.87–3.86 (m, 2H, C*H*-OH, C*H_2_*-N), 2.97 (t, *J* = 12.1 Hz, 1H, C*H_2_*-N), 1.80 (d, *J* = 11.8 Hz, 1H, C*H_2_*-CH-OH), 1.47 (q, *J* = 11.8 Hz, 1H, C*H_2_*-CH-OH), 1.27–1.21 (m, 2H, C*H_2_*-CH_2_-N), 0.92 (t, *J* = 7.7 Hz, 2H, TMS-C*H_2_*), −0.04 (s, 9H, TMS); ^13^C{1H}-NMR (C_6_D_6_, 100 MHz): 172.7 (s, *C*O_2_H), 157.5 (s, Teoc-*C*=O), 68.5 (d, *C*H-OH), 64.9 (t, CO_2_-*C*H_2_), 58.3 (d, *C*_α_), 41.0 (t, *C*H_2_-N), 29.7 (t, *C*H_2_-CH-OH), 23.6 (t, *C*H_2_-CH_2_-N), 17.8 (t, TMS-*C*H_2_), −1.5 (q, TMS); HRMS (ESI) *m*/*z*: [M + Na]^+^ calcd for C_12_H_23_NO_5_SiNa, 312.1238; found: 312.1248; specific rotation [α]_D_^20^ = −22.3° (*c* = 1.0; MeOH).

#### (2*S*,3*R*)-1-(*tert*-Butoxycarbonyl)-3-hydroxypiperidine-2-carboxylic Acid (**6**)

Ester **31** (27 mg, 0.080 mmol, 1.0 equiv) was saponified analogously to ester **30**. The crude product was purified by MPLC (RP-C18, 4 g, 15 min, 0.05% TFA in CH_3_CN/H_2_O, 35–45% CH_3_CN) to obtain Boc-protected 3-hydroxypipecolic acid **6** (17 mg, 0.070 mmol, 89%) as a colorless oil. Main conformer: ^1^H-NMR (CDCl_3_, 400 MHz): 5.38–5.22 (m, 1H, C_α_*H*), 3.76–3.86 (m, 2H, C*H_2_*-N), 2.98–2.77 (m, 1H, C*H*-OH), 1.77–1.63 (m, 1H, C*H_2_*-CH-OH), 1.51–1.45 (m, 1H, C*H_2_*-CH_2_-N), 1.38 (s, 9H, Boc), 1.26–1.06 (m, 2H, C*H_2_*-CH-OH, C*H_2_*-CH_2_-N); ^13^C{1H}-NMR (CDCl_3_, 100 MHz): 172.5 (s, *C*O_2_H), 156.6 (s, Boc-*C*=O), 81.2 (s, Boc-*C_q_*), 68.5 (d, *C*H-OH), 57.9 (d, *C*_α_), 41.3 (t, *C*H_2_-N), 29.7 (t, *C*H_2_-CH-OH), 28.3 (q, Boc), 23.6 (t, *C*H_2_-CH_2_-N). Minor conformer: ^1^H-NMR (CDCl_3_, 400 MHz): 5.13–4.98 (m, 1H, C_α_*H*), 4.19–4.01 (m, 2H, C*H_2_*-N), 2.98–2.77 (m, 1H, C*H*-OH), 1.77–1.63 (m, 1H, C*H_2_*-CH-OH), 1.51–1.45 (m, 1H, H-C*H_2_*-CH_2_-N), 1.38 (s, 9H, Boc), 1.26–1.06 (m, 2H, C*H_2_*-CH-OH, C*H_2_*-CH_2_-N); ^13^C{1H}-NMR (CDCl_3_, 100 MHz): 172.5 (s, *C*O_2_H), 156.6 (s, Boc-*C*=O), 81.2 (s, Boc-*C_q_*), 68.5 (d, *C*H-OH), 57.9 (d, *C*_α_), 41.3 (t, *C*H_2_-N), 29.7 (t, *C*H_2_-CH-OH), 28.3 (q, Boc), 23.6 (t, *C*H_2_-CH_2_-N); HRMS (ESI) *m*/*z*: [M + Na]^+^ calcd for C_11_H_19_NO_5_Na 268.1155; found: 268.1169; specific rotation [α]_D_^20^ = −30.4° (*c* = 1.0; CHCl_3_).

#### 2-Chloro-4-iodo-1-trityl-1*H*-imidazole (**24**)

Imidazole **33** (19.1 g, 43.1 mmol, 1.00 equiv) was dissolved in THF (450 mL) and cooled to −78 °C. Then LDA (1 M in THF, 45.0 mL, 45.0 mmol, 1.05 equiv) was added dropwise. The reaction mixture was stirred at −78 °C for 1 h. C_2_Cl_6_ (11.3 g, 47.6 mmol, 1.10 equiv) was added portion wise to the reaction solution at −78 °C. After 10 min, saturated aqueous NH_4_Cl (150 mL) was added. The solution was warmed to room temperature, and the THF was removed under reduced pressure. Water (150 mL) was added, and the mixture was extracted with ethyl acetate (3 × 400 mL). The combined organic layers were dried over Na_2_SO_4_ and filtered, and the solvent was removed under reduced pressure. The residue was recrystallized from ethanol (250 mL) to obtain pure imidazole **24** (17.5 g, 37.1 mmol, 86%) as a colorless solid. ^1^H-NMR (CDCl_3_, 400 MHz): 7.39–7.37 (m, 9H, Trt), 7.17–7.12 (m, 6H, Trt), 6.94 (s, 1H, Im-*H*); ^13^C{1H}-NMR (CDCl_3_, 100 MHz): 141.0 (Trt), 134.6 (Im-C2), 130.0 (Trt), 129.9 (Trt), 129.1 (Im-C5), 128.2 (Trt), 78.0 (Trt), 77.0 (Im-C4); HRMS (ESI) *m*/*z*: [M + Na]^+^ calcd for C_22_H_16_ClIN_2_Na 492.9944; found: 492.9949; *R*_f_ (petroleum ether:ethyl acetate 9:1): 0.50; melting point: 224 °C.

#### (9*H*-Fluoren-9-yl)methyl ((1*S*,2*S*)-2-(2-Chloro-1-trityl-1*H*-imidazol-4-yl)-2-hydroxy-1-(4-methyl-2,6,7-trioxabicyclo[2.2.2]octan-1-yl)ethyl)carbamate (**23a**) and (9*H*-Fluoren-9-yl)methyl ((1*S*,2*R*)-2-(2-Chloro-1-trityl-1*H*-imidazol-4-yl)-2-hydroxy-1-(4-methyl-2,6,7-trioxabicyclo[2.2.2]octan-1-yl)ethyl)carbamate (**34a**)

Iodo-imidazole **24** (3.68 g, 7.82 mmol, 4.00 equiv) was dissolved in CH_2_Cl_2_ (40 mL) and cooled to 0 °C. Then EtMgBr (1 M in THF, 7.82 mL, 7.82 mmol, 4.00 equiv) was added dropwise. After 1 h, the solution was cooled to −78 °C. Aldehyde **21a** (800 mg, 1.95 mmol, 1.00 equiv) was dissolved in CH_2_Cl_2_ (10 mL) in a separate flask, cooled to −78 °C, and added to the Grignard reaction solution at −78 °C. After 2 h, saturated aqueous NH_4_Cl (40 mL) was added. The solution was warmed to room temperature and diluted with CH_2_Cl_2_ (25 mL). The aqueous layer was extracted with CH_2_Cl_2_ (3 × 25 mL). The combined organic layers were washed with saturated aqueous NH_4_Cl (20 mL), dried over MgSO_4_, and filtered, and the solvent was removed under reduced pressure. The resulting crude product was purified via column chromatography (silica, conditioning with 2% NEt_3_ in *n*-heptane, *n*-heptane to *n*-heptane/ethyl acetate 1:3) to obtain a diastereomeric mixture of **23a** and **34a** (980 mg, 1.3 mmol, 67%, *dr* > 5:1 by ^1^H-NMR and >10:1 by chiral HPLC) as a colorless solid.

HPLC (Chiralpak IE, *n*-heptane/EtOH/MeOH = 72/14/14, flow rate = 1.0 mL/min, l = 265 nm) *t*_R_ = 12.1 (**23a**), 17.7 (**34a**); Main diastereomer **23a**, main conformer: ^1^H-NMR (CDCl_3_, 600 MHz): 7.77–7.28 (m, 8H, aryl-*H*), 7.35–7.06 (m, 15H, Trt), 6.84 (s, 1H, Im-*H*), 5.55 (d, *J* = 10.3 Hz, 1H, N*H*), 5.30 (bs, 1H, C*H*-OH), 4.22/4.17 (d, 2H, Fmoc-C*H_2_*), 4.12 (bs, 1H, Fmoc-C*H*), 4.24 (bs, 1H, C_α_*H*), 3.97 (s, 6H, OBO-C*H_2_*), 3.28 (d, *J* = 2.2 Hz, 1H, O*H*), 0.83 (s, 3H, C*H_3_*); ^13^C{1H}-NMR (CDCl_3_, 150 MHz): 156.5 (s, *C*=O), 144.5 (s, *C_q_*-Fmoc), 144.1 (s, *C_q_*-Fmoc), 141.6 (s, *C_q_*-Trt), 141.4 (s, *C_q_*-Fmoc), 141.3 (s, *C_q_*-Fmoc), 138.6 (s, Im-*C_q_*), 133.4 (s, Im-*C*Cl), 130.1 (d, *C*H-Trt), 127.9 (d, *C*H-Trt), 127.7 (d, *C*H-Trt), 127.22 (d, *C*H-Fmoc), 127.16 (d, *C*H-Fmoc), 125.6 (d, *C*H-Fmoc), 125.5 (d, *C*H-Fmoc), 119.9 (d, Im-*C*H), 108.8 (s, OBO-*C_q_*), 76.3 (s, Trt-*C_q_*), 73.0 (t, OBO-*C*H_2_), 67.30 (t, Fmoc-*C*H_2_), 67.25 (d, *C*HOH), 57.2 (d, *C*_α_), 47.3 (d, Fmoc-*C*H), 30.9 (s, OBO-*C_q_*-CH_3_), 14.5 (q, *C*H_3_); Main diastereomer **23a**, minor conformer: ^1^H-NMR (CDCl_3_, 600 MHz): 7.77–7.28 (m, 8H, aryl-*H*), 7.35–7.06 (m, 15H, Trt), 6.88 (s, 1H, Im-*H*), 5.18 (d, *J* = 10.5 Hz, 1H, N*H*), 5.34 (bs, 1H, C*H*-OH), 4.22/4.17 (d, 2H, Fmoc-C*H_2_*), 4.11 (bs, 1H, Fmoc-C*H*), 4.39 (bs, 1H, C_α_*H*), 3.97 (s, 6H, OBO-C*H_2_*), 3.22 (bs, 1H, O*H*), 0.83 (s, 3H, C*H_3_*); ^13^C{1H}-NMR (CDCl_3_, 150 MHz): 156.3 (s, *C*=O), 144.5 (s, *C_q_*-Fmoc), 144.1 (s, *C_q_*-Fmoc), 141.6 (s, *C_q_*-Trt), 141.4 (s, *C_q_*-Fmoc), 141.3 (s, *C_q_*-Fmoc), 138.8 (s, Im-*C_q_*), 133.1 (s, Im-*C*Cl), 130.1 (d, *C*H-Trt), 127.9 (d, *C*H-Trt), 127.7 (d, *C*H-Trt), 127.22 (d, *C*H-Fmoc), 127.16 (d, *C*H-Fmoc), 125.6 (d, *C*H-Fmoc), 125.5 (d, *C*H-Fmoc), 120.0 (d, Im-*C*H), 108.8 (s, OBO-*C_q_*), 76.3 (s, Trt-*C_q_*), 73.0 (t, OBO-*C*H_2_), 67.73 (d, *C*HOH), 67.30 (t, Fmoc-*C*H_2_), 57.7 (d, *C*_α_), 47.3 (d, Fmoc-*C*H), 30.9 (s, OBO-*C_q_*-CH_3_), 14.5 (q, *C*H_3_); Minor diastereomer **34a**, main conformer: ^1^H-NMR (CDCl_3_, 600 MHz): 7.77–7.28 (m, 8H, aryl-*H*), 7.35–7.06 (m, 15H, Trt), 6.85 (s, 1H, Im-*H*), 5.46 (d, *J* = 10.3 Hz, 1H, N*H*), 4.87 (dd, *J* = 7.6, 3.7 Hz, 1H, C*H*-OH), 4.22/4.17 (d, 2H, Fmoc-C*H_2_*), 4.12 (bs, 1H, C_α_*H*), 4.11 (bs, 1H, Fmoc-C*H*), 3.97 (s, 6H, OBO-C*H_2_),* 3.76 (d, *J* = 3.7 Hz, 1H, O*H*), 0.83 (s, 3H, C*H_3_*); ^13^C{1H}-NMR (CDCl_3_, 150 MHz): 156.5 (s, *C*=O), 144.5 (s, *C_q_*-Fmoc), 144.1 (s, *C_q_*-Fmoc), 141.6 (s, *C_q_*-Trt), 141.4 (s, *C_q_*-Fmoc), 141.3 (s, *C_q_*-Fmoc), 139.2 (s, Im-*C_q_*), 132.5 (s, Im-*C*Cl), 130.1 (d, *C*H-Trt), 127.9 (d, *C*H-Trt), 127.7 (d, *C*H-Trt), 127.22 (d, *C*H-Fmoc), 127.16 (d, *C*H-Fmoc), 125.6 (d, *C*H-Fmoc), 125.5 (d, *C*H-Fmoc), 120.7 (d, Im-*C*H), 108.8 (s, OBO-*C_q_*), 108.6 s, OBO-*C_q_*), 76.3 (s, Trt-*C_q_*), 73.0 (t, OBO-*C*H_2_), 68.2 (d, *C*HOH), 67.30 (t, Fmoc-*C*H_2_), 59.7 (d, *C*_α_), 47.3 (d, Fmoc-*C*H), 30.9 (s, OBO-*C_q_*-CH_3_), 14.5 (q, *C*H_3_); Minor diastereomer **34a**, minor conformer: ^1^H-NMR (CDCl_3_, 600 MHz): 7.77–7.28 (m, 8H, aryl-*H*), 7.35–7.06 (m, 15H, Trt), 6.77 (s, 1H, Im-*H*), 4.95 (d, *J* = 10.3 Hz, 1H, N*H*), 4.81 (bs, 1H, C*H*-OH), 4.22/4.17 (d, 2H, Fmoc-C*H_2_*), 4.12 (bs, 1H, C_α_*H*), 4.11 (bs, 1H, Fmoc-C*H*), 3.97 (s, 6H, OBO-C*H_2_*), 3.83 (d, *J* = 3.7 Hz, 1H, O*H*), 0.83 (s, 3H, C*H_3_*); ^13^C{1H}-NMR (CDCl_3_, 150 MHz): 156.5 (s, *C*=O), 144.5 (s, *C_q_*-Fmoc), 144.1 (s, *C_q_*-Fmoc), 141.6 (s, *C_q_*-Trt), 141.4 (s, *C_q_*-Fmoc), 141.3 (s, *C_q_*-Fmoc), 139.2 (s, Im-*C_q_*), 132.5 (s, Im-*C*Cl), 130.1 (d, *C*H-Trt), 127.9 (d, *C*H-Trt), 127.7 (d, *C*H-Trt), 127.22 (d, *C*H-Fmoc), 127.16 (d, *C*H-Fmoc), 125.6 (d, *C*H-Fmoc), 125.5 (d, *C*H-Fmoc), 120.7 (d, Im-*C*H), 108.6 (s, OBO-*C_q_*), 108.8 (s, OBO-*C_q_*), 76.3 (s, Trt-*C_q_*), 73.0 (t, OBO-*C*H_2_), 68.2 (d, *C*HOH), 67.30 (t, Fmoc-*C*H_2_), 59.7 (d, *C_α_*), 47.3 (d, Fmoc-*C*H), 30.9 (s, OBO-*C_q_*-CH_3_), 14.5 (q, *C*H_3_); 776.2498; found: 776.2499 (M + Na)^+^; *R*_f_ (*n*-heptane/ethyl acetate 3:1): 0.47.

#### *tert*-Butyl ((1*S*,2*S*)-2-(2-Chloro-1-trityl-1*H*-imidazol-4-yl)-2-hydroxy-1-(4-methyl-2,6,7-trioxabicyclo[2.2.2]octan-1-yl)ethyl)carbamate (**23b**) and *tert*-Butyl ((1*S*,2*R*)-2-(2-Chloro-1-trityl-1*H*-imidazol-4-yl)-2-hydroxy-1-(4-methyl-2,6,7-trioxabicyclo[2.2.2]octan-1-yl)ethyl)carbamate (**34b**)

##### Conditions 1 (Scheme 4, Conditions c)

Iodo-imidazole **24** (324 mg, 0.743 mmol, 2.20 equiv) was dissolved in CH_2_Cl_2_ (5 mL) and cooled to 0 °C. Then *i*BuMgBr (2 M in *n-*hexane, 0.370 mL, 0.743 mmol, 2.20 equiv) was added dropwise. After 1 h, the solution was cooled to −78 °C. Aldehyde **21b** (97.0 mg, 0.340 mmol, 1.00 equiv) was dissolved in CH_2_Cl_2_ (5 mL) in a separate flask, cooled to −78 °C, and added to the Grignard reaction solution at −78 °C. After 2 h, saturated aqueous NH_4_Cl (15 mL) was added. The solution was warmed to room temperature and diluted with CH_2_Cl_2_ (10 mL). The aqueous layer was extracted with CH_2_Cl_2_ (3 × 10 mL). The combined organic layers were washed with saturated aqueous NH_4_Cl (2 × 10 mL) and saturated aqueous NaCl (10 mL), dried over Na_2_SO_4_, and filtered, and the solvent was removed under reduced pressure. The resulting crude product was purified via column chromatography (silica, conditioning with 2% NEt_3_ in petroleum ether, petroleum ether to petroleum ether/ethyl acetate 1:2) to obtain a diastereomeric mixture of **23b** and **34b** (177 mg, 0.280 mmol, 83%, *dr* 1.8:1 by ^1^H-NMR) as a colorless solid. HPLC (Chiralpak IE, *n*-heptane/EtOH/MeOH = 72/14/14, flow rate = 1.0 mL/min, l = 202 nm) *t*_R_ = 9.3 (**23b**), 11.5 (**34b**).

##### Conditions 2 (Scheme 4, Conditions d)

Iodo-imidazole **24** (836 mg, 1.78 mmol, 3.00 equiv) was dissolved in THF (9 mL) and cooled to −78 °C. Then *n*-BuLi (1.54 M in *n-*hexane, 1.15 mL, 1.18 mmol, 3.00 equiv) was added dropwise, and the reaction mixture was stirred for 30 min. Aldehyde **21b** (170 mg, 0.592 mmol, 1.00 equiv) was dissolved in THF (6 mL) in a separate flask, cooled to −78 °C, and added to the lithium reaction solution at −78 °C. After 5 h, saturated aqueous NH_4_Cl (10 mL) was added. The solution was warmed to room temperature and diluted with CH_2_Cl_2_ (10 mL). The aqueous layer was extracted with CH_2_Cl_2_ (3 × 10 mL). The combined organic layers were washed with saturated aqueous NH_4_Cl (10 mL) and saturated aqueous NaCl (10 mL), dried over Na_2_SO_4_, and filtered, and the solvent was removed under reduced pressure. The resulting crude product was purified *via* column chromatography (silica, conditioning with 2% NEt_3_ in petroleum ether, petroleum ether/ethyl acetate 4:1 to 100% ethyl acetate) to obtain **23b** and **34b** as a colorless solid and a mixture of diastereomers with a *dr* of 5:1(determined by ^1^H-NMR). The diastereomers were separated by a second purification *via* flash chromatography (25 g PF-15SIHC flash column, conditioning with 2% NEt_3_ in petroleum ether, 0–100% ethyl acetate in *n*-heptane in 60 min) to obtain the desired main diastereomer **23b** (231 mg, 0.364 mmol, 62%) as a colorless solid (all fractions containing traces of **23b** and the undesired minor diastereomer **34b** were discarded).

##### Conditions 3 (Scheme 4, Conditions e)

To a solution of the diastereomeric mixture of **23a** and **34a** (282 mg, 0.374 mmol, *dr* > 5:1, 1.00 equiv) in THF (10 mL) was added aqueous NHMe_2_ (40%, 1.00 mL, 7.93 mmol, 21.2 equiv), and the reaction mixture was stirred at room temperature for 30 min until LC/MS indicated complete Fmoc-deprotection. Toluene (30 mL) was added, and all volatiles were removed *in vacuo*. The crude product was further dried in high vacuum (0.1 mbar) overnight to remove all traces of NHMe_2_. The crude mixture was dissolved in THF (10 mL) and saturated aqueous NaHCO_3_ (1 mL), followed by Boc_2_O (245 mg, 1.12 mmol, 3.00 equiv) were added and the reaction mixture was stirred at room temperature overnight. Ethyl acetate (30 mL) and water (5 mL) were added, and the layers were separated. The organic layer was washed with saturated aqueous NaCl (5 mL) and dried over Na_2_SO_4_. After filtration the solvent was removed under reduced pressure and the resulting crude product was purified *via* flash chromatography (25 g PF-15SIHC flash column, conditioning with 2% NEt_3_ in petroleum ether, 0%–100% ethyl acetate in *n*-heptane in 60 min) to obtain the desired main diastereomer **23b** (153 mg, 0.242 mmol, 65%) as a colorless solid (all fractions containing traces of **23b** and the undesired minor diastereomer **34b** were discarded). Main diastereomer **23b**: ^1^H-NMR (CDCl_3_, 400 MHz): 7.31–7.27 (m, 9H, Trt), 7.17–7.09 (m, 6H, Trt), 6.82 (s, 1H, Im-H), 5.24 (d, *J* = 10.4 Hz, 1H, CH-OTIPS), 5.21 (bs, 1H, NH), 4.03 (d, *J* = 10.1 Hz, 1H, C_α_H), 3.93 (s, 6H, OBO-CH_2_), 3.23 (d, *J* = 1.9 Hz, 1H. OH), 1.35 (s, 9H, Boc), 0.80 (s, 3H, OBO-CH_3_); ^13^C{1H}-NMR (CDCl_3_, 100 MHz): 155.8 (s, Boc-C=O), 141.7 (s, Trt), 138.8 (s, Im), 133.2 (s, OBO-C_q_), 130.1 (d, Trt), 128.0 (d, Trt), 127.9 (d, Trt), 119.8 (d, Im-CH), 108.9 (s, Im), 79.1 (s, Trt-C_q_), 76.3 (s, Boc-C_q_), 72.8 (t, OBO-CH_2_), 67.2 (d, C-OH), 56.6 (d, C_α_), 30.8 (s, OBO-C_q_), 28.5 (q, Boc-CH_3_), 14.5 (q, OBO-CH_3_); HRMS (ESI) *m*/*z*: [M + H]^+^ calcd for C_35_H_39_N_3_O_6_Cl 632.2527; found: 632.2525; *R*_f_ (3% MeOH in CH_2_Cl_2_): 0.25; specific rotation [α]_D_^20^ = −7° (*c* = 1.0; CHCl_3_). Minor diastereomer **34b** (the chemical shifts for **34b** were determined from the mixture derived from conditions 1): ^1^H-NMR (CDCl_3_, 400 MHz): 7.31–7.27 (m, 9H, Trt), 7.17–7.09 (m, 6H, Trt), 6.80 (s, 1H, Im-H), 5.21 (bs, 1H, NH), 5.13 (d, *J* = 10.04 Hz, 1H, CH-OTIPS), 4.03 (d, *J* = 10.1 Hz, 1H, C_α_H), 3.85 (s, 6H, OBO-CH_2_), 3.23 (s, 1H. OH), 1.35 (s, 9H, Boc), 0.78 (s, 3H, OBO-CH_3_); ^13^C{1H}-NMR (CDCl_3_, 100 MHz): 155.8 (s, Boc-C=O), 141.7 (s, Trt), 138.8 (s, Im), 133.2 (s, OBO-C_q_), 130.1 (d, Trt), 128.0 (d, Trt), 127.9 (d, Trt), 119.8 (d, Im-CH), 108.5 (s, Im), 79.3 (s, Trt-C_q_), 76.2 (s, Boc-C_q_), 72.6 (t, OBO-CH_2_), 68.2 (d, C-OH), 58.4 (d, C_α_), 30.7 (s, OBO-C_q_), 28.5 (q, Boc-CH_3_), 14.5 (q, OBO-CH_3_).

#### *tert*-Butyl ((1*S*,2*S*)-2-(2-Chloro-1-trityl-1*H*-imidazol-4-yl)-1-(4-methyl-2,6,7-trioxabicyclo[2.2.2]octan-1-yl)-2-((triisopropylsilyl)oxy)ethyl)carbamate (**35**)

Alcohol **23b** (100 mg, 0.158 mmol, 1.00 equiv) was dissolved in CH_2_Cl_2_ (1.6 mL) and cooled to −78 °C. 2,6-Lutidine (129 μL, 1.27 mmol, 8.00 equiv) and TIPS-OTf (170 μL, 0.633 mmol, 4.00 equiv) were added dropwise, and the reaction mixture was stirred for 5 h at −78 °C. Saturated aqueous NH_4_Cl (3 mL) was added. The aqueous layer was extracted with CH_2_Cl_2_ (3 × 10 mL). The combined organic layers were dried over Na_2_SO_4_ and filtered, and the solvent was removed under reduced pressure. The resulting crude product was purified *via* column chromatography (silica, 1% MeOH in CH_2_Cl_2_) to obtain **35** (117 mg, 0.148 mmol, 94%) as a colorless solid. ^1^H-NMR (CDCl_3_, 400 MHz): 7.32–7.26 (m, 9H, Trt), 7.15–7.08 (m, 6H, Trt), 6.70 (s, 1H, Im-*H*), 5.36 (m, 1H, C*H*-OTIPS), 5.12 (d, *J* = 10.3 Hz, 1H, N*H*), 3.93 (d, *J* = 10.3 Hz, 1H, C_α_*H*), 3.84 (s, 6H, OBO-C*H_2_*), 1.34 (s, 9H, Boc-C*H_3_*), 0.97 (s, 21H, TIPS), 0.76 (s, 3H, OBO-C*H_3_*); ^13^C{1H}-NMR (CDCl_3_, 100 MHz): 155.8 (s, Boc-*C*=O), 142.3 (s, Im-*C*Cl), 141.8 (s, Trt), 132.5 (s, OBO-*C_q_*), 130.1 (d, Trt), 128.0 (d, Trt), 127.8 (d, Trt), 120.2 (d, Im-*C*H), 108.2 (s, Im), 78.6 (s, Trt-*C_q_*), 76.0 (s, Boc-*C_q_*), 72.6 (t, OBO-*C*H_2_), 68.4 (d, *C*H-OTIPS), 58.6 (d, *C*_α_H), 30.6 (s, OBO-*C_q_*), 28.6 (q, Boc-*C*H_3_), 18.13 (q, TIPS-*C*H_3_), 18.10 (q, TIPS-*C*H_3_), 14.6 (q, OBO-*C*H_3_), 12.9 (d, TIPS-*C*H); HRMS (ESI) *m*/*z*: [M + H]^+^ calcd for C_44_H_59_N_3_O_6_SiCl 788.3862; found: 788.3862; *R*_f_ (petroleum ether/ethyl acetate 1:1): 0.63; specific rotation [α]_D_^20^ = −24.7° (*c* = 1.0; CHCl_3_).

#### 3-Hydroxy-2-(hydroxymethyl)-2-methylpropyl (2*S*,3*S*)-2-((*tert*-Butoxycarbonyl)amino)-3-(2-chloro-1*H*-imidazol-4-yl)-3-((triisopropylsilyl)oxy)propanoate (**36**)

OBO ester **35** (44 mg, 0.056 mmol, 1.0 equiv) was dissolved in 2,2,2-trifluoroethanol (0.6 mL), and aqueous AcOH (80%, 0.6 mL) was added subsequently. The reaction mixture was warmed to 30 °C and stirred overnight. Then the mixture was cooled to room temperature, and aqueous saturated NaHCO_3_ (2 mL) was added. After dilution with ethyl acetate (8 mL), the layers were separated. The organic layer was dried over Na_2_SO_4_ and filtered, and the solvent was removed under reduced pressure. Diol **36** (32 mg, 0.056 mmol, quant.) was obtained as a yellowish oil. The highly polar crude product was utilized in the next step without further purification.

#### Methyl (2*S*,3*S*)-2-((*tert*-Butoxycarbonyl)amino)-3-(2-chloro-1*H*-imidazol-4-yl)-3-((triisopropylsilyl)oxy)propanoate (**37**)

Diol **36** (32 mg, 0.056 mmol, 1.0 equiv) was dissolved in MeOH (1.9 mL), and K_2_HPO_4_ (291 mg, 1.67 mmol, 30.0 equiv) was added subsequently. The reaction mixture was warmed to 40 °C and stirred for 48 h. Then the reaction mixture was cooled to room temperature, diluted with CH_2_Cl_2_ (10 mL), and filtered over Celite. The solvent was removed under reduced pressure, and the crude product was purified *via* column chromatography (silica, 1–3% MeOH in CH_2_Cl_2_) to obtain methylester **37** (22 mg, 0.046 mmol, 83%) as a colorless solid. ^1^H-NMR (CDCl_3_, 400 MHz): 6.88 (s, 1H, Im-H), 5.67–5.36 (m, 1H, N*H*), 5.37–5.09 (m, 1H, C*H*-OTIPS), 4.56–4.38 (m, 1H, C_α_*H*), 3.75 (s, 3H, CH_3_), 1.43 (s, 9H, Boc), 0.99 (s, 21H, TIPS); ^13^C{1H}-NMR (CDCl_3_, 100 MHz): 171.5 (s, CH_3_O-*C*=O), 156.0 (s, Boc-*C*=O), 142.6 (s, Im-*C_q_*), 130.0 (s, Im-*C*Cl), 115.1 (d, Im-*C*H), 80.6 (s, Boc-*C_q_*), 70.5 (d, *C*-OTIPS), 59.2 (d, *C*_α_), 52.5 (q, *C*H_3_), 28.4 (q, Boc-*C*H_3_), 18.0 (q, TIPS-*C*H_3_), 17.9 (q, TIPS-*C*H_3_), 12.5 (d, TIPS-*C*H).; HRMS (ESI) *m*/*z*: [M + Na]^+^ calcd for C_21_H_38_N_3_O_5_SiClNa 498.2161; found: 498.2169; *R*_f_ (5% MeOH in CH_2_Cl_2_): 0.45; specific rotation [α]_D_^25^ = −18.4° (*c* = 1.0; CHCl_3_); HPLC (Chiralpak IC, *n*-heptane/EtOH/MeOH = 94/3/3, flow rate = 1.0 mL/min, *l* = 212 nm) *t*_R_ = 6.1 min.

#### Methyl (2*S*,3*S*)-2-Amino-3-(2-chloro-1*H*-imidazol-4-yl)-3-((triisopropylsilyl)oxy)propanoate (**22**)

The deprotection of methylester **37** was performed based on our previously reported procedure.^4^ Dihydrochloride **22** was obtained as a colorless solid and was used without further purification.

#### 2-(Trimethylsilyl)ethyl (5*S*,7*S*,10*S*,13*S*)-7-Azido-5-((carbamoyloxy)methyl)-13-((*S*)-(2-chloro-1*H*-imidazol-4-yl)((triisopropylsilyl)oxy)methyl)-2,2,3,3-tetramethyl-8,11-dioxo-10-((1-trityl-1*H*-imidazol-4-yl)methyl)-4-oxa-9,12-diaza-3-silatetradecan-14-oate (**45**)

Carboxylic acid **43** (99 mg, 0.14 mmol, 1.0 equiv) was dissolved in CH_2_Cl_2_/DMF (1.4 mL, 1:1), and the resulting reaction mixture was cooled to −40 °C. Oxyma (44 mg, 0.31 mmol, 2.2 equiv), EDC · HCl (59 mg, 0.31 mmol, 2.2 equiv), and NaHCO_3_ (118 mg, 1.41 mmol, 10.0 equiv) were added, and the suspension was stirred for 5 min before hydrochloride **44**(4) (90 mg, 0.17 mmol, 1.2 equiv) was added in one portion. The suspension was slowly warmed to room temperature and stirred for 48 h. After addition of water (30 mL) and ethyl acetate (40 mL), the layers were separated, and the aqueous layer was extracted with ethyl acetate (3 × 40 mL). The combined organic layers were washed with saturated aqueous NH_4_Cl (70 mL), H_2_O (70 mL), saturated aqueous NaHCO_3_ (70 mL), and saturated aqueous NaCl (70 mL), and then dried over Na_2_SO_4_. After filtration, the solvent was removed under reduced pressure, and the resulting crude product was purified *via* column chromatography (silica, petroleum ether:ethyl acetate 5:1) to obtain **45** (130 mg, 0.112 mmol, 80% over two steps) as a light orange foam. ^1^H-NMR (CDCl_3_, 400 MHz): 12.58 (bs, 1H, Im-N*H*), 7.87 (m, 1H, Im), 7.82–7.72 (m, 1H, N*H*), 7.57–7.40 (m, 1H, N*H*) 7.38–7.30 (m, 9H, Trt), 7.15–7.06 (m, 6H, Trt), 6.82 (m, 1H, Cl-Im), 6.66 (s, 1H, Im), 5.32 (m, 1H, TIPSO-C*H*), 4.93 (dd, 1H, *J* = 6.9, 4.3 Hz, C*H*-CH_2_-Im), 4.78 (br s, 2H CON*H*_2_), 4.64–4.39 (m, 1H, C*H*-CO_2_TMSE), 4.14–3.91 (m, 5H, C*H*_2_-C*H*OTBS, C*H*_2_-CH_2_-TMS), 3.16–2.98 (m, 2H, C*H_2_*-Im), 2.09–1.97 (m, 1H, N_3_-CH-C*H*_2_), 1.86–1.71 (m, 1H, N_3_-CH-C*H*_2_), 1.05–0.86 (m, 32H, TIPS, TBS, C*H*_2_-TMS), 0.14–0.06 (m, 6H, TBS), 0.05–0.02 (m, 9H, TMS); ^13^C{1H}-NMR (CDCl_3_, 100 MHz): 170.6 (N_3_-CH-*C*O-NH), 170.1 (CH-*C*O-NH), 169.3 (*C*O_2_TMSE), 156.4 (s, *C*ONH_2_), 141.9 (Trt), 138.8 (Im), 136.7 (Im), 129.7 (Trt), 128.3 (Trt), 128.2 (Trt), 120.3 (Cl-Im), 68.1 (NH-CO_2_-*C*H_2_), 66.9 (*C*H-OTIPS), 64.2 (*C*H-OTBS), 60.4 (N_3_-*C*H), 59.1 (NH-*C*H-CO_2_TMSE), 55.1 (NH-*C*H-CO-NH), 45.8 (CO_2_-*C*H_2_), 37.0 (*C*H_2_-CH-OTBS), 25.83 (TBS), 25.79 (*C*H_2_-TMS), 18.1 (s, TBS), 17.8 (TIPS), 17.7 (TIPS), 12.0 (TIPS), −1.5 (TMS), −4.3 (q, TBS), −4.9 (q, TBS); HRMS (ESI) *m*/*z*: [M + H]^+^ calcd for C_57_H_84_ClN_10_O_8_Si_3_ 1155.5470; found: 1155.5475; *R*_f_ (8% MeOH in CH_2_Cl_2_): 0.55.

#### 2-(Trimethylsilyl)ethyl (5*S*,7*S*,10*S*,13*S*)-7-Amino-5-((carbamoyloxy)methyl)-13-((*S*)-(2-chloro-1*H*-imidazol-4-yl)((triisopropylsilyl)oxy)methyl)-2,2,3,3-tetramethyl-8,11-dioxo-10-((1-trityl-1*H*-imidazol-4-yl)methyl)-4-oxa-9,12-diaza-3-silatetradecan-14-oate (**46**)

Azide **45** (130 mg, 0.112 mmol, 1.00 equiv) was dissolved in degassed THF/H_2_O (1.5 mL, 5:1). Trimethylphosphine (1 M in THF, 134 μL, 0.134 mmol, 1.20 equiv) was added, and the resulting reaction mixture was stirred for 12 h at room temperature. All volatiles were removed under reduced pressure and the resulting crude product **46** was used in the following peptide coupling without further purification.

#### 2-(Trimethylsilyl)ethyl (2*R*,3*S*)-2-(((7*S*,10*S*,13*S*,15*S*)-15-((Carbamoyloxy)methyl)-7-((*S*)-(2-chloro-1*H*-imidazol-4-yl)((triisopropylsilyl)oxy)methyl)-2,2,17,17,18,18-hexamethyl-6,9,12-trioxo-10-((1-trityl-1*H*-imidazol-4-yl)methyl)-5,16-dioxa-8,11-diaza-2,17-disilanonadecan-13-yl)carbamoyl)-3-hydroxypiperidine-1-carboxylate (**47**)

Acid **5** (39 mg, 0.13 mmol, 1.2 equiv) was dissolved in CH_2_Cl_2_/DMF (1.2 mL, 1:1), and the resulting mixture was cooled to 0 °C. After addition of Oxyma (35 mg, 0.25 mmol, 2.2 equiv), EDC·HCl (47 mg, 0.25 mmol, 2.2 equiv), and NaHCO_3_ (49 mg, 0.56 mmol; 5.0 equiv), the suspension was stirred for 5 min before amine **46** (crude product from previous reaction, 0.112 mmol, 1.00 equiv) dissolved in CH_2_Cl_2_/DMF (1.0 mL, 1:1) was added dropwise. The suspension was slowly warmed to room temperature and was stirred for 12 h. After hydrolysis by addition of water (20 mL) and ethyl acetate (30 mL), the layers were separated, and the aqueous layer was extracted with ethyl acetate (3 × 40 mL). The combined organic layers were washed with saturated aqueous NH_4_Cl (40 mL), water (40 mL), saturated aqueous NaHCO_3_ (40 mL), and saturated aqueous NaCl (40 mL) and finally dried over Na_2_SO_4_. After filtration, the solvent was removed under reduced pressure, and the resulting crude product was purified *via* column chromatography (silica, 2–6% MeOH in CH_2_Cl_2_) to obtain tetrapeptide **47** (129 mg, 0.0920 mmol, 82% over 2 steps) as a colorless solid. ^1^H-NMR and ^13^C{1H}-NMR: broad signals even by utilizing a variety of solvents, no meaningful spectra; HRMS (ESI)**:***m*/*z*: [M + H]^+^ calcd for C_69_H_107_ClN_9_O_12_Si_4_ 1400.6799; found: 1400.6809; *R*_f_ (6% MeOH in CH_2_Cl_2_): 0.45.

#### (2*S*,3*S*)-2-((*S*)-2-((2*S*,4*S*)-5-(Carbamoyloxy)-4-hydroxy-2-((2*S*,3*R*)-3-hydroxypiperidine-2-carboxamido)pentanamido)-3-(1-trityl-1*H*-imidazol-4-yl)propanamido)-3-(2-chloro-1*H*-imidazol-4-yl)-3-hydroxypropanoic Acid (**48**)

Tetrapeptide **47** (129 mg, 0.0920 mmol, 1.00 equiv) was dissolved in DMF (0.6 mL) and water (33.1 μL, 1.84 mmol, 20.0 equiv), and then TAS-F (1 M in DMF, 1.84 mL, 1.84 mmol, 20.0 equiv) was added. The reaction mixture was stirred for 48 h. The reaction mixture was directly applied to MPLC (RP-C18, 4 g, 20 min, 0.1% FA in CH_3_CN/H_2_O, 0–80% CH_3_CN) in order to obtain tetrapeptide **48** (55 mg, 0.062 mmol, 67%) as a colorless solid. ^1^H-NMR and ^13^C{1H}-NMR: broad signals even by utilizing a variety of solvents, no meaningful spectra; HRMS (ESI) *m*/*z*: [M + H]^+^ calcd for C_43_H_49_ClN_9_O_10_ 886.3285; found: 886.3285; *R*_f_ (20% MeOH in CH_2_Cl_2_): 0.21.

#### (2*S*,3*S*)-2-((*S*)-2-((2*S*,4*S*)-5-(Carbamoyloxy)-4-hydroxy-2-((2*S*,3*R*)-3-hydroxypiperidine-2-carboxamido)pentanamido)-3-(1*H*-imidazol-4-yl)propanamido)-3-(2-chloro-1*H*-imidazol-4-yl)-3-hydroxypropanoic Acid (GE81112A, **1**)

Tetrapeptide **48** (55 mg, 0.062 mmol, 1.0 equiv) was dissolved in TFE/formic acid (1 mL, 9:1), and the resulting solution was stirred for 12 h. The reaction was stopped by dilution with water (5 mL) and direct freeze drying. The crude product was purified *via* flash chromatography (RP-18, 15 min, 0–80% MeCN (0.1% formic acid)) to obtain GE81112A (**1**, 27 mg, 0.042 mmol, 67%) as a colorless solid. To generate the TFA salt, **1** was lyophilized with a solution of 0.05% TFA in water. ^1^H-NMR **(**DMSO-d_6_, 500 MHz) and ^13^C{1H}-NMR **(**DMSO-d_6_, 100 MHz) are in full accordance with the previously reported data^4^ (spectra see the Supporting Information); HRMS (ESI) *m*/*z*: [M + H]^+^ calcd for C_24_H_35_ClN_9_O_10_ 644.2190; found: 644.2186; specific rotation [α]_D_^25^ = +21.4° (*c* = 1.1; MeOH; Lit.: +23.7°^4^).

## Data Availability

The data underlying this study are available in the published article and its online Supporting Information.

## Supporting Information Available

Detailed experimental procedures including spectroscopic data and determination of enantiomeric excess (PDF)
