# Supplementary material for: Total Synthesis of GE81112A: An Orthoester-Based Approach
Source: J Org Chem. 2023 Apr 6;88(9):5597–608. doi: 10.1021/acs.joc.3c00094 (PMC10167690; doi:10.1021/acs.joc.3c00094)
Supplement: Supplementary file 1 — jo3c00094_si_001.pdf [file jo3c00094_si_001.pdf]

Supporting Information

Total Synthesis of GE81112A: An Orthoester-based Approach

Scherin Fayad [a, b], Ardalan Jafari [b], Sören M. M. Schuler[c, d], Michael Kurz[a], Oliver Plettenburg[b], Peter E. Hammann[d, e], Armin Bauer[a], Gerrit Jürjens[b, c] ,*, Christoph Pöverlein[a],*

[a] Sanofi-Aventis Deutschland GmbH, R&D, Integrated Drug Discovery, Industriepark Hoechst, 65926 Frankfurt am Main, Germany
[b] Helmholtz Zentrum München, Deutsches Forschungszentrum für Gesundheit und Umwelt (GmbH), Institut für Medizinalchemie, 30167 Hannover, Germany
[c] Fraunhofer Institute for Molecular Biology and Applied Ecology IME, Branch for Bioresources, 35392 Giessen, Germany
[d] Evotec International GmbH, 37079 Göttingen, Germany
[e] Sanofi-Aventis Deutschland GmbH, R&D, Infectious Diseases, Industriepark Hoechst, 65926 Frankfurt am Main, Germany

*gerrit.juerjens@helmholtz-muenchen.de, christoph.poeverlein@sanofi.com

Table of Contents

1. Synthesis of building blocks **20**, **21a-c**, **32**, **37**, and **44**. SI-2
2. NMR Spectra SI-14
3. Determination of the enantiomeric excesses (*ee*) SI-47

1. Synthesis of building blocks 20, 21a-c, 32, 37, and 44.

Scheme S1. Synthesis of alkyne 20 and serinals 21a-c.[a]

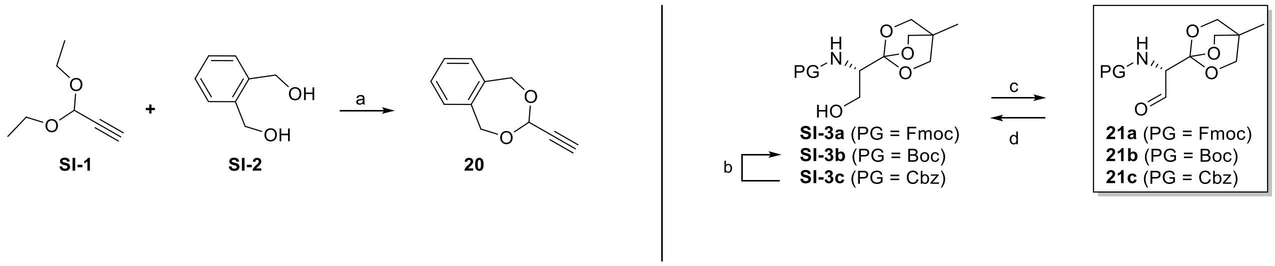

[a]Conditions: (a) *p*TsOH · H_2O (0.05 eq.), toluene, 60 °C, 72 h, 84%; (b) H_2, Pd/C, Boc_2O, THF, rt, 18 h, then $LiOH_{(aq.)}$ (1 M), THF, rt, 30 min, quant. (c) $(COCl)_2$, DMSO, DIPEA, DCM, −78 °C to −20 °C, 2.5 h, quant. (d) $LiBH_4$, THF, rt, quant. (for the chiral analysis of the unstable aldehydes **21a-c**).

3-Ethynyl-1,5-dihydrobenzo[e][1,3]dioxepine (20):

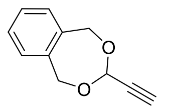

20

1,2-Benzenedimethanol (**SI-2**, 6.00 g, 43.4 mmol, 1.00 eq.), 3,3-diethoxyprop-1-ine (**SI-1**, 6.68 g, 52.1 mmol, 1.20 eq.) and *p*-toluene sulfonic acid monohydrate (413 mg, 2.17 mmol, 0.05 eq.) were dissolved in toluene (0.44 M, 100 mL) and stirred at 60 °C. After 72 h the solvent was removed under reduced pressure. The resulting crude product was purified by column chromatography (silica, *n*-heptane to *n*-heptane/EtOAc 1:1) to obtain alkyne **20** (6.33 g, 36.3 mmol, 84%) as a colorless solid.

^{1}H-NMR ($CDCl_3$, 600 MHz): 7.22 (m, 2H, aryl-*H*), 7.13 (m, 2H, aryl-*H*), 5.61 (d, *J* = 1.6 Hz, 1H, C*H*), 5.13 (d, *J* = 14.3 Hz, 2H, C*H₂*), 4.83 (d, *J* = 14.3 Hz, 2H, C*H₂*), 2.58 (d, *J* = 1.6 Hz, 1H, C≡C-*H*); **^{13}C{1H}-NMR** ($CDCl_3$, 150 MHz): 138.1 (s, Ar-C), 127.3 (d, Ar-*C*), 126.9 (d, Ar-*C*), 93.3 (d, *C*H), 79.2 (s, *C*≡C-H), 73.4 (d, C≡*C*-H), 68.1 (t, *C*H_2); **HRMS (ESI)** m/z $[M+Na]^+$ calcd. for $C_{11}H_{10}NaO_2$ 197.0573; found: 197.0572; **R_f** (*n*-heptane/ethyl acetate 3:1): 0.92.

***tert*-Butyl (*S*)-(2-hydroxy-1-(4-methyl-2,6,7-trioxabicyclo[2.2.2]octan-1-yl)ethyl)carbamate (SI-3b):**

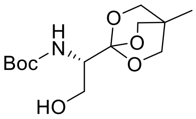

SI-3b

Cbz-protected alcohol **SI-3c**[1] (890 mg, 2.75 mmol, 1.00 eq.) was dissolved in THF (0.2 M, 14 mL). Boc_2O (720 mg, 3.30 mmol, 1.20 eq.) and Pd/C (10 mol%, 291 mg, 0.275 mmol, 0.10 eq.) were added and the reaction mixture was hydrogenated at room temperature under a hydrogen atmosphere (1 bar) for 18 h. After completion of the reaction the catalyst are filtrated by Celite® and the solvent was removed under reduced pressure. Afterwards the crude product was dissolved in THF (0.2 M, 14 mL), aqueous LiOH (1 M, 2.75 mL, 2.75 mmol, 1.00 eq.) was added and the reaction mixture was stirred for 30 min at room temperature.[2] After dilution with ethyl acetate (50 mL) the mixture was washed with saturated aqueous NaCl (20 mL) and the layers were separated. The organic layer was dried over Na_2SO_4 and the solvent was removed under reduced pressure to obtain the crude product **SI-3b**[1] (790 mg, 2.73 mmol, quant.), which was used without further purification.

^{1}H-NMR ($CDCl_3$, 400 MHz): 5.07 (s, 1H, N*H*), 3.91 (s, 6H, OBO-CH_2), 3.85–3.62 (m, 3H, CH_2 and $C_\alpha H$), 2.66 (br s, 1H, O*H*), 1.44 (s, 9H, Boc), 0.81 (s, 3H, OBO-CH_3); **^{13}C{1H}-NMR** ($CDCl_3$, 100 MHz): 156.1 (s, C=O), 108.7 (s, $C_\alpha H$-C_q), 79.8

[1] Blaskovich, M. A.; Evindar, G.; Rose, N. G. W.; Wilkinson, S.; Luo, Y.; Lajoie, G. A. *Stereoselective Synthesis of Threo and Erythro β-Hydroxy and β-Disubstituted-β-Hydroxy α-Amino Acids. J. Org. Chem.* **1998**, *63*, 3631–3646.
[2] Under the reaction condition, we monitored traces of the *O*-Boc-protected side-product of **SI-3b**. A short treatment with LiOH hydrolyzed this unwanted side-product quantitatively.

(s, Boc-C_q), 72.8 (t, OBO-CH_2), 62.4 (d, $C_\alpha H$), 55.1 (t, CH_2-OH), 30.7 (s, C_q-CH_3), 28.5 (q, Boc-CH_3), 14.4 (q, OBO-CH_3);[3]
R_f (CH_2Cl_2/ethyl acetate 4:1): 0.47.

General Procedure A: Swern oxidation of SI-3a-c with suppressed racemization:

21a-c were synthesized according to a slightly modified literature-known procedure[4]: The reaction was carried out in moisture-free glassware under inert atmosphere. To a solution of $(COCl)_2$ (1.70 eq.) in anhydrous DCM (4 mL/mmol of $(COCl)_2$) anhydrous DMSO (3.20 eq.) was added carefully at −78 °C. After stirring for 15 min, alcohol **SI-3a-c** (1.00 eq.) dissolved in anhydrous DCM (6 mL/mmol of **SI-3a-c**) was added. The reaction mixture was stirred for 1.5 h at −78 °C. Then DIPEA (6.23 eq.) was added and the solution was stirred for 30 min at −78 °C and for further 30 min without cooling bath. A mixture of cooled toluene/saturated aqueous NH_4Cl (4:1, 50 mL/mmol of **SI-3a-c**, pre-cooled to 0 °C) was added to the reaction mixture. The layers were separated and the organic layer was washed with saturated aqueous NH_4Cl (3 × 20 mL/mmol of **SI-3a-c**, pre-cooled to 0 °C), with saturated aqueous $NaHCO_3$ (20 mL/mmol of **SI-3a-c**, pre-cooled to 0 °C), and with saturated aqueous NaCl (20 mL/mmol of **SI-3a-c**, pre-cooled to 0 °C). The organic layer was dried over $MgSO_4$, filtered, and concentrated under reduced pressure to yield crude aldehyde **21a-c** (quant.) as slightly yellow oil, which was used in the next stage without further purification.

General Procedure B: Swern oxidation of SI-3a-c with (partial) racemization by column chromatography on silica:

***Rac*-21a-c** were synthesized starting from **SI-3a-c** in a similar manner with an additional purification step *via* chromatography which led to partial/full epimerization of the stereocenter[5]: The reaction was carried out in moisture-free glassware under inert atmosphere. To a solution of $(COCl)_2$ (1.70 eq.) in anhydrous DCM (4 mL/mmol) anhydrous DMSO (3.20 eq.) was added carefully at −78 °C. After stirring for 15 min, alcohol **SI-3a-c** (1.00 eq.) dissolved in anhydrous DCM (6 mL/mmol) was added. The reaction mixture was stirred for 1.5 h at −78 °C. Then DIPEA (6.23 eq.) was added and the solution was stirred for 30 min at −78 °C and for further 30 min without cooling bath. Saturated aqueous NH_4Cl (10 mL/mmol) and DCM (20 mL/mmol) were added to the reaction mixture, the layers were separated, and the organic layer was washed with saturated aqueous NH_4Cl (10 mL/mmol), with saturated aqueous $NaHCO_3$ (10 mL/mmol), and with saturated aqueous NaCl (10 mL/mmol). The organic layer was dried over $MgSO_4$, filtered, and concentrated under reduced pressure. The crude product was purified by flash column chromatography[6] (100% DCM, then DCM : EtOAc 4:1) to give *rac*-**21a-c** (80–90%) as a colorless solid/oil and was directly used for the next step.

General Procedure C: Reduction of aldehyde 21a-c to alcohol SI-3a-c for *ee*-determination.

To a solution of aldehyde **21a-c** (10 mg, 0.03 mmol, 1.0 eq.) in THF (1 mL) was added $LiBH_4$ (4 mg, 0.2 mmol, 5 eq.) and the reaction mixture was stirred for 15 min until TLC indicated complete reduction of the aldehyde. Excess of $LiBH_4$ was quenched by addition of saturated aqueous NH_4Cl (1 mL). Ethyl acetate (5 mL) was added and the layers were separated. The organic layer was washed by saturated aqueous NaCl (1 mL). The organic layer was dried over $MgSO_4$, filtered and

[3] The shifts of the ^1H and $^{13}C\{1H\}$ signals slightly differed from the signals reported in literature (Blaskovich, M. A.; Evindar, G.; Rose, N. G. W.; Wilkinson, S.; Luo, Y.; Lajoie, G. A. *Stereoselective Synthesis of Threo and Erythro β-Hydroxy and β-Disubstituted-β-Hydroxy α-Amino Acids. J. Org. Chem.* **1998**, *63*, 3631–3646). Most likely, the differences are related to the used concentrations and the NMR-spectrometers.
[4] Rose, N. G. W.; Blaskovich, M. A.; Evindar, G.; Wilkinson, S.; Luo, Y.; Fishlock, D.; Reid, C.; Lajoie, G. A. *Preparation of 1-[N-benzyloxycarbonyl-(1S)-1-amino-2-oxoethyl]-4-methyl-2,6,7-trioxabicyclo[2.2.2]octane. Org. Synth.* **2002**, *79*, 216-227.
[5] The epimerization of the stereocenter of OBO-protected serinals *via* chromatography is a known phenomenon and was described by Blaskovich and Lajoie (*J. Am. Chem. Soc.* **1993**, *115*, 5021–5030). In our case, the purification *via* column chromatography resulted in measured *ee*-values of 0–6% for **21a** and **21c** and 40–58% for **21b**.
[6] Conditioning of the silica column was performed with a mixture of DCM and NEt_3 (98:2) to neutralize the slightly acidic silica and to prevent partial ortho ester hydrolysis during chromatography.

concentrated under reduced pressure. The crude product (10 mg, quant.) was directly used for chiral chromatography to determine the *ee* of alcohols **SI-3a-c** and to deduce the *ee* of the corresponding aldehydes **21a-c** thereof.

(9*H*-Fluoren-9-yl)methyl (*S*)-(1-(4-methyl-2,6,7-trioxabicyclo[2.2.2]octan-1-yl)-2-oxoethyl)carbamate (21a):

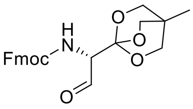

21a

Aldehyde **21a**[7] was prepared according to general procedure A from **SI-3a** and obtained in quantitative yield as a colorless oil which was used for the next step without any further purification or analytics. It is recommended to use crude aldehyde **21a** as quickly as possible (ideally at the same day or with maximum one night storage in a freezer) for the next reaction as it tends to epimerize at longer storage times. $\mathbf{R_f}$ (*n*-heptane/ethyl acetate 3:1): 0.65. The *ee* was determined at a later stage of the synthesis for compound **23a** (*ee* = 98%).

***tert*-Butyl (*S*)-(1-(4-methyl-2,6,7-trioxabicyclo[2.2.2]octan-1-yl)-2-oxoethyl)carbamate (21b):**

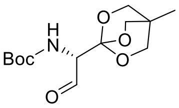

21b

Aldehyde **21b**[1] was prepared according to general procedure A from **SI-3b** and obtained in quantitative yield as a colorless oil which was used for the next step without any further purification or analytics. It is recommended to use crude aldehyde **21a** as quickly as possible (ideally at the same day or with maximum one night storage in a freezer) for the next reaction as it tends to epimerize at longer storage times. $\mathbf{R_f}$ (3% MeOH in CH_2Cl_2): 0.45. The *ee*-determination of **21b** was not possible by reduction to alcohol **SI-3b** and chiral chromatography due to the lack of an UV chromophor. The *ee* was determined at a later stage of the synthesis for compound **37** (*ee* > 99%).

Benzyl (*S*)-(1-(4-methyl-2,6,7-trioxabicyclo[2.2.2]octan-1-yl)-2-oxoethyl)carbamate (21c):

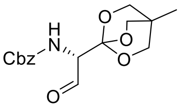

21c

Aldehyde **21c**[1] was prepared according to general procedure A from **SI-3c** and obtained in quantitative yield as a colorless oil which was used for the next step without any further purification or analytics.It is recommended to use crude aldehyde **21c** as quickly as possible (ideally at the same day or with maximum one night storage in a freezer) for the next reaction as it tends to epimerize at longer storage times. $\mathbf{R_f}$ (*n*-heptane/ethyl acetate 3:1): 0.74. For the *ee*-determination, a small sample of crude aldehyde **21c** was reduced according to general procedure C and the *ee* of the resulting alcohol **SI-3c** was determined by chiral chromatography (a partial racemic reference sample of **SI-3c** was obtained by general procedure B): *ee* = 97%. **SI-3c: HPLC** (Chiralpak AD-H, EtOH/MeOH = 50/50, flow rate = 1.0 mL/min, l = 208 nm) t_R = 6.1 min (**SI-3c**), 11.3 min (***ent*-SI-3c**).

[7] Blaskovich, M. A.; Lajoie, G. A. *Synthesis of a Chiral Serine Aldehyde Equivalent and its Conversion to Chiral Alpha-Amino-Acid Derivatives, J. Am. Chem. Soc.* **1993**, *115*, 5021–5030.

Scheme S2. Oxidation-reduction strategy for the preparation of 25 and conversion to methyl ester 32.[a]

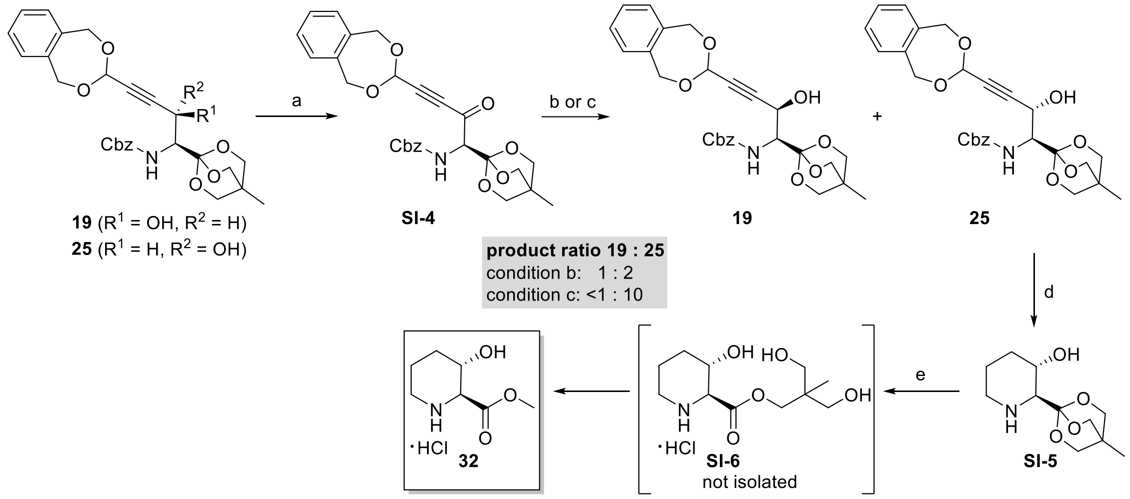

[a]Conditions: (a) $(COCl)_2$, DMSO, DIPEA, CH_2Cl_2, −78 °C to −20 °C, 2.5 h, quant.; (b) **SI-4**, $LiBH_4$ (3 eq.), THF, −78 °C to rt, 24 h, 50%, (*dr* = 1 :2) (c) **SI-4**, $LiBHEt_3$ (4 eq.), THF, −78 °C, 2 h, 81% (*dr* < 1:10); (d) H_2 (85 bar), $Pd(OH)_2$/C, THF, 70 °C; (e) AcCl, MeOH, 70 °C, 80% over two steps.

Benzyl (*S*)-(4-(1,5-dihydrobenzo[e][1,3]dioxepin-3-yl)-1-(4-methyl-2,6,7-trioxabicyclo[2.2.2]octan-1-yl)-2-oxobut-3-yn-1-yl)carbamate (SI-4):

Ketone **SI-4** was synthesized following general procedure A for Swern oxidation with suppressed racemization: Starting from a diastereomeric mixture of **19** and **25** (0.34 g, 0.69 mmol, 1.0 eq.), crude ketone **SI-4** (0.34 g, 0.69 mmol, quant.) was obtained as a colorless solid and was used without further purification for the reduction step. $\mathbf{R_f}$ (*n*-heptane/ethyl acetate 1:3): 0.71.

Benzyl ((1*S*,2*R*)-4-(1,5-dihydrobenzo[e][1,3]dioxepin-3-yl)-2-hydroxy-1-(4-methyl-2,6,7-trioxabicyclo[2.2.2]oc-tan-1-yl)but-3-yn-1-yl)carbamate (19) and benzyl ((1*S*,2*S*)-4-(1,5-dihydrobenzo[e][1,3]dioxepin-3-yl)-2-hydroxy-1-(4-methyl-2,6,7-trioxabicyclo[2.2.2]octan-1-yl)but-3-yn-1-yl)carbamate (25):

Condition 1 (Reduction with $LiBH_4$; Scheme SI 2, condition b). A solution of ketone **SI-4** (0.34 g, 0.69 mmol, 1.0 eq.) in THF (15 mL) was cooled to −78 °C and $LiBH_4$ (4.0 M in THF, 1.0 mL, 4.1 mmol, 6.0 eq.) was added. The reaction mixture was stirred at −78 °C for 2 h, for another 22 h without cooling bath at room temperature, and was then diluted with saturated aqueous NH_4Cl (30 mL) and ethyl acetate (80 mL).The layers were separated and the organic layer was washed with saturated aqueous NaCl (30 mL), and was dried over $MgSO_4$, filtered, and the solvent was removed under reduced pressure. The crude product was purified *via* column chromatography (silica, conditioning with 2% NEt_3 in *n*-heptane, 0–100% ethyl acetate in *n*-heptane) to obtain the diastereomeric mixture of **19** and **25** (0.12 g, 0.24 mmol, 35%, *dr* 1:1.1)[8] and pure diastereomer **25** (50 mg, 0.10 mmol, 15%) as colorless solids. The total *dr* was calculated as 1:2.

Condition 2 (Reduction with $LiBHEt_3$; Scheme SI-2, condition c). A solution of ketone **SI-4** (0.19 g, 0.39 mmol, 1.0 eq.) in THF (15 mL) was cooled to −78 °C and $LiBHEt_3$ (1.0 M in THF, 1.6 mL, 1.6 mmol, 4.0 eq.) was added. The reaction mixture was stirred at −78 °C for 2 h and was then diluted with saturated aqueous NH_4Cl (20 mL) and ethyl acetate (50 mL).The layers were separated and the organic layer was washed with saturated aqueous NaCl (20 mL), was dried over $MgSO_4$, filtered, and the solvent was removed under reduced pressure. The crude product was purified *via* column chromatography (silica, conditioning with 2% NEt_3 in *n*-heptane, 0–100% ethyl acetate in *n*-heptane) to obtain the diastereomeric mixture of **19** and **25** (0.16 g, 0.32 mmol, 81%, *dr* < 1:10)[8] as a colorless solid.

[8] The *dr* was determined by ^{1}H-NMR.

(2*S*,3*S*)-2-(4-Methyl-2,6,7-trioxabicyclo[2.2.2]octan-1-yl)piperidin-3-ol (SI-5):

Alkyne **25** (70 mg, 0.14 mmol, 1.0 eq.) was dissolved in THF (30 mL). The reaction mixture was hydrogenated by the H-Cube® at 70 °C and 85 bar in the presence of $Pd(OH)_2$ (20 mol%). After completion of the reaction the solvent was removed under reduced pressure. Amine **SI-5** (32 mg, 0.14 mmol, quant.) was obtained as a yellowish oil. The acid labile, high polar crude product was used without further analytics.

HRMS (ESI) m/z $[M+H]^+$ calcd. for $C_{11}H_{20}NO_4$ 230.1387; found: 230.1396.

(2*S*,3*S*)-3-Hydroxy-2-(methoxycarbonyl)piperidine hydrochloride (32):

Anhydrous MeOH (2.3 mL) was cooled to 0 °C. Acetyl chloride (1 mL) was added dropwise and the reaction mixture was stirred at room temperature for 20 min. Afterwards, OBO-ester **SI-5** (32 mg, 0.14 mmol, 1.0 eq.) was dissolved in MeOH (1 mL) and added dropwise. The reaction mixture was heated to 70 °C with an oil bath and was stirred for 10 days.[9] The solvent was removed under reduced pressure and the crude product was applied to a cation exchange chromatography (Dowex 50WX8 200-400, MeOH) in order to obtain methyl ester hydrochloride **32** (22 mg; 0.11 mmol; 80% over 2 steps) as a colorless oil.

^{1}H-NMR (D_2O, 400 MHz): 4.13–4.08 (dt, 1H, *J* = 8.2, 3.25 Hz, C*H*-OH), 3.92–3.90 (d, 1H, *J* = 8.2 Hz, $C_\alpha H$), 3.85 (s, 3H, C*H₃*), 3.37 (m, 1H, CH_2-NH), 3.07 (m, 1H, CH_2-NH), 2.69 (s, 1H, OH), 2.02 (m, 2H, CH_2-CH_2-OH, CH_2-CH_2-N), 1.69 (m, 2H, CH_2-CH_2-OH, CH_2-CH_2-N); **^{13}C{1H}-NMR** (D_2O, 100 MHz): 168.4 (s, *C*=O), 65.6 (d, *C*H-OH), 60.8 (d, C_α), 53.7 (q, CH_3), 42.8 (t, CH_2-N), 29.1 (t, CH_2-CH-OH), 18.8 (t, CH_2-CH_2-N); **HRMS (ESI)** m/z $[M+H]^+$ calcd. for $C_7H_{14}NO_3$ 160.0968; found: 160.0967.[10]

[9] LC/MS indicated that transesterification of compound **SI-6** to methyl ester **33** was the rate-limiting step.

[10] Compound **SI-6** was only obtained starting from a partially racemic starting material **25**. Therefore, no specific rotation was measured.

Scheme S3. Synthetic route to diastereomers 37 and SI-9 from the diastereomeric mixture of 23b and 34b.[a]

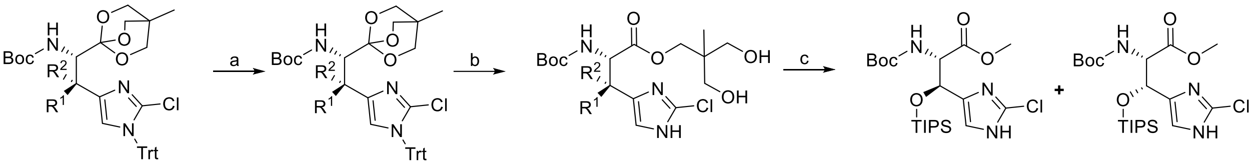

[a]Conditions: (a) TIPSOTf, 2,6-lutidine, CH_2Cl_2, −78 °C, 5 h, 80% (b) aq. AcOH (80%), TFE, 30 °C, 24 h; (c) K_2HPO_4, MeOH, 40 °C, 72 h, 74% over 2 steps (as a mixture); after HPLC purification: **37**, 52% over 2 steps and **SI-9**, 18% over 2 steps.

***tert*-Butyl ((1*S*,2*S*)-2-(2-chloro-1-trityl-1*H*-imidazol-4-yl)-1-(4-methyl-2,6,7-trioxabicyclo[2.2.2]octan-1-yl)-2-((triisopropylsilyl)oxy)ethyl)carbamate (35) and *tert*-Butyl ((1*S*,2*R*)-2-(2-chloro-1-trityl-1*H*-imidazol-4-yl)-1-(4-methyl-2,6,7-trioxabicyclo[2.2.2]octan-1-yl)-2-((triisopropylsilyl)oxy)ethyl)carbamate (SI-7):**

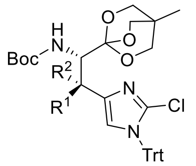

35 (R1= OTIPS, R2 = H)
SI-7 (R1= H, R2 = OTIPS)
inseparable mixture

A mixture of the diastereomeric alcohols **23b** and **34b** (60 mg, 0.095 mmol, 1.0 eq., *dr* 1.8:1) was dissolved in CH_2Cl_2 (0.1 M, 1.0 mL) and cooled to −78 °C. 2,6-Lutidine (43 µL, 0.42 mmol, 4.4 eq.) and TIPS-OTf (56 µL, 0.21 mmol, 2.2 eq.) were added dropwise and the reaction mixture was stirred for 18 h at −78 °C. Saturated, aqueous NH_4Cl (3 mL) was added. The aqueous layer was extracted with CH_2Cl_2 (3x 10 mL). The combined organic layers were dried over Na_2SO_4, filtered and the solvent was removed under reduced pressure. The resulting crude product was purified *via* column chromatography (silica, 1% MeOH in CH_2Cl_2) to obtain an inseparable mixture of **35** and **SI-7** (60 mg, 0.076 mmol, 80%, *dr* 1.8:1)[8] as a colorless solid.[11]

Main diastereomer **35**: **^{1}H NMR** ($CDCl_3$, 400 MHz): 7.32–7.26 (m, 9H, Trt), 7.15–7.08 (m, 6H, Trt), 6.70 (s, 1H, Im-*H*), 5.36 (m, 1H, C*H*OTIPS), 5.12 (d, *J* = 10.3 Hz, 1H, N*H*), 3.93 (d, *J* = 10.3 Hz, 1H, C_α*H*), 3.84 (s, 6H, OBO-C*H2*), 1.34 (s, 9H, Boc-C*H3*), 0.97 (s, 21H, TIPS), 0.76 (s, 3H, OBO-C*H3*); **^{13}C{1H}-NMR** ($CDCl_3$, 100 MHz,): 155.8 (s, Boc-*C*=O), 142.3 (s, Im-*C*Cl), 141.8 (s, Trt), 132.5 (s, OBO-C_q), 130.1 (d, Trt), 128.0 (d, Trt), 127.8 (d, Trt), 120.2 (d, Im-*C*H), 108.2 (s, Im), 78.6 (s, Trt-C_q), 76.0 (s, Boc-C_q), 72.6 (t, OBO-CH_2), 68.4 (d, *C*HOTIPS), 58.6 (d, C_αH), 30.6 (s, OBO-C_q), 28.6 (q, Boc-CH_3), 18.1 (q, TIPS-CH_3), 18.1 (q, TIPS-CH_3), 14.6 (q, OBO-CH_3), 12.9 (d, TIPS-*C*H).

Minor diastereomer **SI-7** (the chemical shifts for **SI-7** were determined from the mixture): **^{1}H-NMR** ($CDCl_3$, 400 MHz): 7.32–7.26 (m, 9H, Trt), 7.15–7.08 (m, 6H, Trt), 6.72 (s, 1H, Im-*H*), 5.36 (m, 1H, C*H*OTIPS), 5.12 (d, *J* = 10.3 Hz, 1H, N*H*), 3.93 (d, *J* = 10.3 Hz, 1H, C_α*H*), 3.83 (s, 6H, OBO-C*H2*), 1.36 (s, 9H, Boc-C*H3*), 0.94 (s, 21H, TIPS), 0.73 (s, 3H, OBO-C*H3*); **^{13}C{1H}-NMR** ($CDCl_3$, 100 MHz): 156.2 (s, *C*=O), 142.3 (s, Im-*C*Cl), 141.8 (s, Trt), 132.5 (s, OBO-C_q), 130.1 (d, Trt), 128.0 (d, Trt), 127.8 (d, Trt), 120.2 (d, Im-*C*H), 107.9 (s, Im), 78.7 (s, Trt-C_q), 75.8 (s, Boc-C_q), 72.34(t, OBO-CH_2), 68.4 (d, *C*HOTIPS), 59.2 (d, C_αH), 30.6 (s, OBO-C_q), 28.6 (q, Boc-CH_3), 18.2 (q, TIPS-CH_3), 18.1 (q, TIPS-CH_3), 14.7 (q, OBO-CH_3), 12.3 (d, TIPS-*C*H). **HRMS (ESI)** m/z $[M+H]^+$ calcd. for $C_{44}H_{59}N_3O_6SiCl$ 788.3856; found: 788.3862; **R_f** (petroleum ether/ethyl acetate 1:1): 0.63.

[11] Due to the high lipophilicity and instability during the run, the diastereomeric ratio was not determined by HPLC at this point.

3-Hydroxy-2-(hydroxymethyl)-2-methylpropyl (2*S*,3*S*)-2-((tert-butoxycarbonyl)amino)-3-(2-chloro-1*H*-imidazol-4-yl)-3-((triisopropylsilyl)oxy)propanoate (36) and 3-Hydroxy-2-(hydroxymethyl)-2-methylpropyl (2*S*,3*R*)-2-((tert-butoxycarbonyl)amino)-3-(2-chloro-1*H*-imidazol-4-yl)-3-((triisopropylsilyl)oxy)propanoate (SI-8):

A mixture of diastereomers **35** and **SI-7** (112.0 mg, 0.142 mmol, 1.00 eq., *dr* 1.8:1) was dissolved in 2,2,2-trifluoroethanol (1.4 mL) and aqueous AcOH (80%, 1.4 mL) was added subsequently. The reaction mixture was warmed to 30 °C and stirred over night. Then the mixture was cooled to room temperature and saturated aqueous $NaHCO_3$ (2 mL) was added. After dilution with ethyl acetate (6 mL) the layers were separated. The organic layer was dried over Na_2SO_4, filtered and the solvent was removed under reduced pressure. Crude mixture of diols **36** and **SI-8** (80 mg, 0.14 mmol, quant.) was obtained as a yellowish oil. The high polar crude product was utilized in the next step without further purification.

Methyl (2*S*,3*S*)-2-((*tert*-butoxycarbonyl)amino)-3-(2-chloro-1*H*-imidazol-4-yl)-3-((triisopropylsilyl)oxy)propanoate (37) and methyl (2*S*,3*R*)-2-((*tert*-butoxycarbonyl)amino)-3-(2-chloro-1*H*-imidazol-4-yl)-3-((triisopropylsilyl) oxy)propanoate (SI-9):

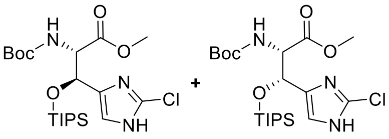

37 **SI-9**

separated by preparative HPLC

The crude mixture of diastereomeric diols **36** and **SI-8** (80 mg, 0.14 mmol, 1.0 eq.) was dissolved in MeOH (4.7 mL) and K_2HPO_4 (742 mg, 4.26 mmol, 30.0 eq.) was added subsequently. The reaction mixture was warmed to 40 °C and stirred for 72 h. Then the reaction mixture was cooled to room temperature, diluted with CH_2Cl_2 (15 mL) and filtered over Celite®. The solvent was removed under reduced pressure and the crude product was purified *via* column chromatography (silica, 1-3% MeOH in CH_2Cl_2) to obtain methyl esters **37** and **SI-9** (50 mg, 0.11 mmol, 74% over two steps) as a colorless solid of a mixture of diastereomers with *dr* 1.8:1. The diastereomers were separated by a second purification *via* preparative HPLC (75%–85% acetonitrile in 15 min). To the product containing fractions (approx. 40 mL acetonitrile/water mixture each), ethyl acetate (100 mL) and saturated aqueous $NaHCO_3$ (20 mL) were added. The layers were separated and the aqueous layer was extracted with ethyl acetate (3 × 20 mL). The combined organic layers were washed with saturated aqueous NaCl (20 mL), were dried over $MgSO_4$, filtered, and the solvent was removed under reduced pressure to obtain main diastereomer **37** (35 mg, 0.074 mmol, 52%) and minor diastereomer **SI-9** (12 mg, 0.025 mmol, 18%) as colorless solids.

Main diastereomer **37**: **^{1}H-NMR** ($CDCl_3$, 400 MHz): 6.88 (s, 1H, Im-H), 5.67–5.36 (m, 1H, N*H*), 5.37–5.09 (m, 1H, C*H*OTIPS), 4.56–4.38 (m, 1H, $C_\alpha H$), 3.75 (s, 3H, CH_3), 1.43 (s, 9H, Boc), 0.99 (s, 21H, TIPS); **^{13}C{1H}-NMR** ($CDCl_3$, 100 MHz): 171.5 (s, CH_3O-*C*=O), 156.0 (s, Boc-*C*=O), 142.6 (s, Im-C_q), 130.0 (s, Im-*C*Cl), 115.1 (d, Im-*C*H), 80.6 (s, Boc-C_q), 70.5 (d, *C*-OTIPS), 59.2 (d, C_α), 52.5 (q, CH_3), 28.43 (q, Boc-CH_3), 18.0 (q, TIPS-CH_3), 17.9 (q, TIPS-CH_3), 12.5 (d, TIPS-*C*H); **HRMS** (ESI) m/z $[M+Na]^+$ calcd. for $C_{21}H_{38}N_3O_5SiClNa$ 498.2161; found: 498.2169; **R_f** (5% MeOH in CH_2Cl_2): 0.45; **Specific rotation** $[\alpha]_D^{25}$ = −18.4° (c = 1.0; $CHCl_3$).

Minor diastereomer **SI-9**: **^{1}H-NMR** ($CDCl_3$, 400 MHz): 6.89 (s, 1H, Im-H), 5.45–5.34 (m, 1H, N*H*), 5.28–5.21 (m, 1H, C*H*OTIPS), 4.72–4.62 (m, 1H, $C_\alpha H$), 3.73 (s, 3H, CH3), 1.42 (s, 9H, Boc), 1.05–0.97 (m, 21H, TIPS); **^{13}C{1H}-NMR** ($CDCl_3$, 100 MHz): 170.7 (s, CH_3O-*C*=O), 155.6 (s, Boc-*C*=O), 137.9 (s, Im-C_q), 130.8 (s, Im-*C*Cl), 119.7 (d, Im-*C*H), 80.2 (s, Boc-C_q), 69.5 (d, *C*-OTIPS), 59.9 (d, *Cα*), 52.6 (q, CH_3), 28.4 (q, Boc-CH_3), 18.0 (q, TIPS-CH_3), 18.0 (q, TIPS-CH_3), 12.4 (d, TIPS-*C*H); **HRMS** (ESI) m/z $[M+Na]^+$ calcd. for $C_{21}H_{38}N_3O_5SiClNa$ 498.2161; found: 498.2169; **R_f** (5% MeOH in CH_2Cl_2): 0.45.[12]

[12] The minor diastereomer **SI-9** was obtained in a test sequence starting from a partially racemic aldehyde **21b**. Therefore, no specific rotation was measured.

Scheme S4. Synthesis of hydroxy-histidine intermediates 22 and 44.[a]

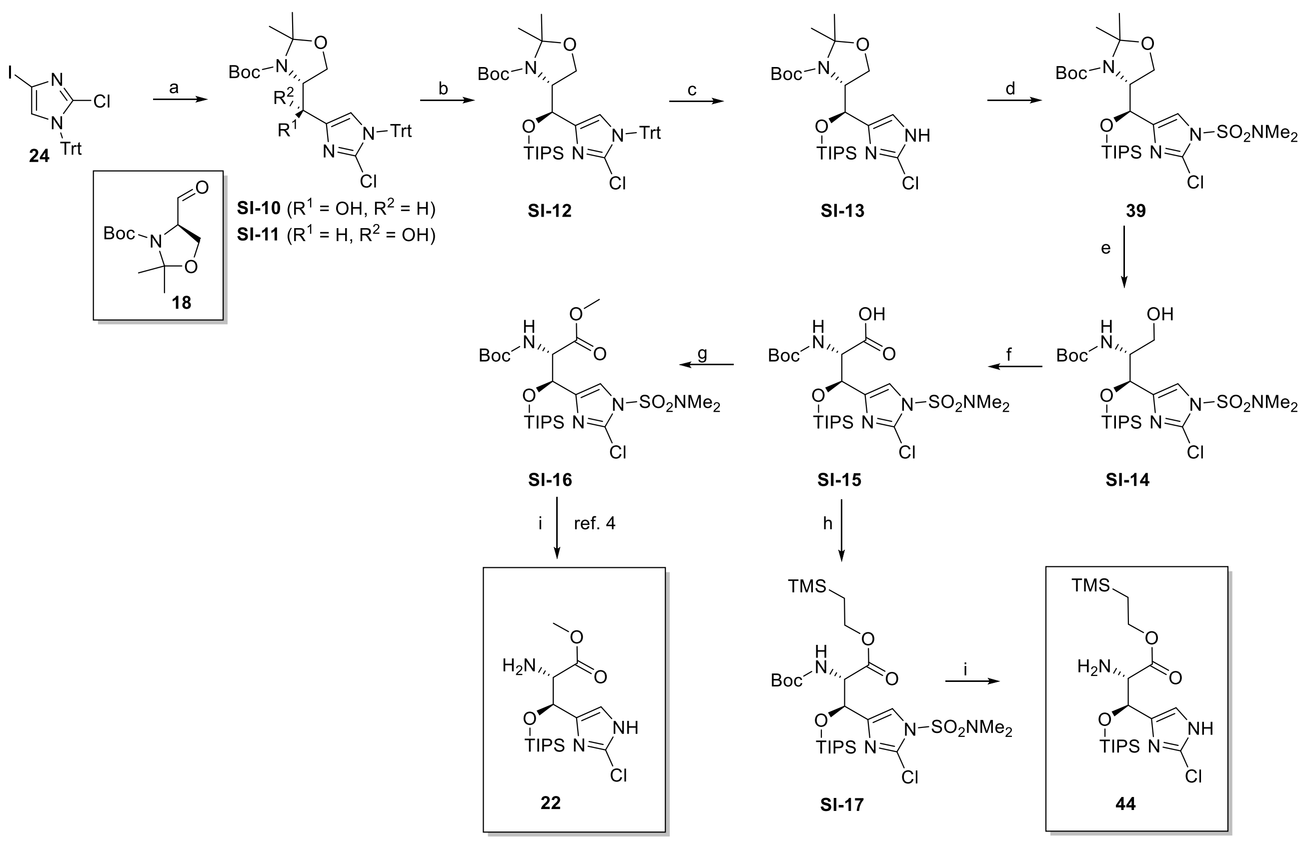

[a]Conditions: (a) *i*-PrMgBr, CH_2Cl_2, 0 °C, 30 min, then **18**, −78 °C, 4 h, 73% (**SI-10**/**SI-11** *dr* 10:1); b) TIPSOTf, 2,6-lutidine, CH_2Cl_2, 0 °C, 2 h, 72%; c) CSA, TFE, rt, 12 h, 90%; d) SO_2NMe_2Cl, NaH, CH_2Cl_2, 0 °C, 12 h, 90%; e) 1 M $HCl_{(aq.)}$/AcOH (1:9), rt, 80%; f) 2.4 M $NaOCl_{(aq.)}$,TEMPO, $NaClO_2$, MeCN, rt, 12 h; g) $TMSCHN_2$, CH_2Cl_2/MeOH, rt, 5 min, 67% over two steps; h) DIC, DMAP, $Me_3SiCH_2CH_2OH$, CH_2Cl_2, 0 °C to rt, 12 h, 85% over two steps, i) 4 M HCl in 1,4-dioxane, THF, rt, 12 h.

***tert*-Butyl (*R*)-4-((*S*)-(2-chloro-1-trityl-1*H*-imidazol-4-yl)(hydroxy)methyl)-2,2-dimethyloxazolidine-3-carboxylate (SI-10) and *tert*-(Butyl (*R*)-4-((*R*)-(2-Chloro-1-trityl-1*H*-imidazol-4-yl)(hydroxy)methyl)-2,2-dimethyloxazolidine-3-carboxylate (SI-11):**

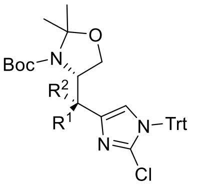

SI-10 (R^1 = OH, R^2 = H)
SI-11 (R^1 = H, R^2 = OH)

To a stirred solution of imidazole **24** (36.1 g, 76.8 mmol, 1.10 eq.) in CH_2Cl_2 (500 mL) *i*-PrMgBr (2 M in Et_2O, 40.1 mL, 80.3 mmol, 1.15 eq.) was added dropwise at 0 °C. After stirring at this temperature for 30 min, the reaction mixture was cooled to −78 °C. At this temperature, a solution of aldehyde **18** (16.0 g, 69.8 mmol, 1.00 eq.) dissolved in CH_2Cl_2 (200 mL) was transferred *via* cannula. The reaction mixture was stirred at this temperature for 4 h. After addition of saturated aqueous NH_4Cl (600 mL) and phase separation, the aqueous layer was extracted with CH_2Cl_2 (1.80 L). The combined organic layers were dried over Na_2SO_4, filtered, and concentrated under reduced pressure. After purification (silica, petroleum ether/ethyl acetate 1:1 to 1:2) the diastereomeric mixture of **SI-10** and **SI-11** (33.1 g, 57.6 mmol, 75%, *dr* 10:1)[8] was obtained as off-white foam.

^{1}H-NMR ($CDCl_3$, 400 MHz): **Major diastereomer SI-10**, **main rotamer**: 7.36–7.27 (m, 9H, Trt-*H*), 7.17–7.08 (m, 6H, Trt-*H*), 6.83 (s, 1H, Im-*H*), 4.87 (m, 1H, C*H*OH), 4.70 – 4.58 (m, 1H, O*H*), 4.40–4.25 (m, 1H, oxazolidine-C*H*$_2$), 4.15–3.89 (m, 2H, oxazolidine-C*H*, oxazolidine-C*H*$_2$), 1.52–1.39 (m, 15H, acetonide-Me$_2$, Boc); **minor rotamer:** 7.36–7.27 (m, 9H, Trt-*H*), 7.17–7.08 (m, 6H, Trt-*H*), 6.83 (s, 1H, Im-*H*), 5.00 (m, 1H, C*H*OH), 4.70–4.59 (m, 1H, O*H*), 4.40–4.25 (m, 1H, oxazolidine-C*H*$_2$), 4.15–3.89 (m, 2H, oxazolidine-C*H*, oxazolidine-C*H*$_2$), 1.52–1.39 (m, 15H, acetonide-Me$_2$, Boc). **Minor**

diastereomer SI-11: 7.36–7.27 (m, 9H, Trt-*H*), 7.17–7.08 (m, 6H, Trt-*H*), 6.94 (s, 1H, Im-*H*), 3.80–3.70 (m, 2H, oxazolidine-C*H*$_2$), 3.64 (m, 1H, oxazolidine-C*H*), 1.28–1.12 (m, 15H, acetonide-Me$_2$, Boc); **^{13}C{1H}-NMR** (CDCl$_3$, 100 MHz): **Major diastereomer** :141.6 (Im-*C*4), 130.1 (Trt), 128.0 (Im-*C*2), 121.3 (Im-*C*5), 77.4 (Trt), 76.4 (Boc), 69.5 (*C*HOH), 62.7 (oxazolidine-*C*H$_2$), 63.2 (oxazolidine-*C*H), 28.6 (Boc), 26.3 (acetonide-Me), 24.0 (acetonide-Me), **Minor diastereomer**: 141.5 (Im-*C*4), 129.8 (Trt), 127.4 (Im-*C*2), 121.3 (Im-*C*5), 77.4 (Trt), 76.4 (Boc), 69.5 (*C*HOH), 62.7 (oxazolidine-*C*H$_2$), 63.2 (oxazolidine-*C*H), 29.8 (Boc), 25.3 (acetonide-Me), 20.9 (acetonide-Me); **HRMS (ESI)** m/z [M+Na]$^+$ calcd. for $C_{33}H_{36}ClN_3O_4Na$ 596.2287; found: 596.2300; **R$_f$** (petroleum ether/ethyl acetate 5:1): 0.2.

***tert*-Butyl (*R*)-4-((*S*)-(2-chloro-1-trityl-1*H*-imidazol-4-yl)((triisopropylsilyl)oxy)methyl)-2,2-dimethyloxazolidine-3-carboxylate (SI-12):**

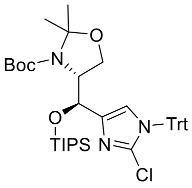

SI-12

To a stirred solution of the diastereomeric mixture of the secondary alcohols **SI-10** and **SI-11** (9.87 g, 17.2 mmol, 1.00 eq.) in CH_2Cl_2 (170 mL), 2,6-lutidine (3.00 mL, 25.8 mmol, 1.50 eq.) and TIPSOTf (4.90 mL, 18.1 mmol, 1.05 eq.) were added at −78 °C. After stirring for 2 h the reaction was stopped by addition of saturated aqueous $NaHCO_3$ (100 mL) and the reaction mixture was warmed to room temperature. The layers were separated and the aqueous layer was extracted with CH_2Cl_2 (3 × 150 mL). The combined organic layers were washed with saturated aqueous NaCl (100 mL), dried over Na_2SO_4, filtered, and concentrated under reduced pressure. MeOH (130 mL) was added to the off-white foam and the mixture was heated to 65 °C with an oil bath until complete dissolution. The solution was slowly cooled to room temperature as a colorless precipitate was formed. The precipitate was filtered off and the mother liquor was crystallized again by evaporating the MeOH, then dissolving the residue in MeOH (50 mL) and heating the mixture to 65 °C in an oil bath to fully dissolve the off-white foam. After cooling, the precipitate was filtered off, dried and combined with the first batch. The desired product **SI-12** (9.09 g, 12.4 mmol, 72%) was obtained as a single diastereomer.

^{1}H-NMR (CDCl$_3$, 400 MHz): **Main rotamer**: 7.32–7.31 (t, 9H, *J* = 2 Hz, Trt-*H*), 7.16–7.15 (m, 6H, Trt-*H*), 6.75 (s, 1H, Im-*H*), 5.19 (d, 1H, *J* = 2.9 Hz, OTIPS-C*H*), 4.48–4.46 (d, 1H, *J* = 6.2 Hz, oxazolidine-C*H*$_2$), 4.03 (m, 1H, oxazolidine-C*H*$_2$), 3.94 (t, 1H, *J* = 7.5 Hz, oxazolidine-C*H*), 1.62 (s, 3H, acetonide-Me$_2$), 1.44 (m, 9H, Boc), 1.01–0.96 (m, 21H, TIPS); **minor rotamer:** 7.32–7.31 (t, 9H, *J* = 2 Hz, Trt-*H*), 7.16–7.15 (m, 6H, Trt-*H*), 6.69 (s, 1H, Im-*H*), 5.28 (m, 1H, OTIPS-C*H*), 4.32 (m, 1H, oxazolidine-C*H*$_2$), 4.03 (m, 1H, oxazolidine-C*H*$_2$), 3.84 (m, 1H, oxazolidine-C*H*), 1.59 (s, 3H, acetonide-Me$_2$), 1.46 (m, 9H, Boc), 1.01–0.96 (m, 21H, TIPS); **^{13}C{1H}-NMR** (CDCl$_3$, 100 MHz): 141.5 (Im-*C*4), 130.0 (Trt), 127.9 (Im-*C*2), 120.9 (Im-*C*5), 79.8 (Trt), 76.0 (Boc), 68.12 (*C*HOTIPS), 63.6 (oxazolidine-*C*H$_2$), 63.2 (oxazolidine-*C*H), 28.5 (Boc), 26.9 (acetonide-Me), 25.0 (acetonide-Me), 18.1 (TIPS-Me), 18.0 (TIPS-Me), 12.8 (TIPS-*C*H); **HRMS (ESI)** m/z [M+Na]$^+$ calcd. for $C_{42}H_{56}ClN_3O_4SiNa$ 752.3621; found: 752.3619; **R$_f$** (petroleum ether/ethyl acetate 9:1): 0.2; **Specific rotation** $[\alpha]_D^{25.6}$ = +20.8 (c = 1.07; CHCl$_3$).

***tert*-Butyl (*R*)-4-((*S*)-(2-chloro-1*H*-imidazol-4-yl)((triisopropylsilyl)oxy)methyl)-2,2-dimethyloxazolidine-3-carboxylate (SI-13):**

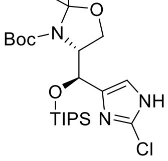

SI-13

To a solution of **SI-12** (9.09 g, 12.4 mmol, 1.00 eq.) in CF_3CH_2OH (120 mL) was added a catalytic amount of (±)-camphorsulfonic acid (29 mg, 0.12 mmol, 0.10 eq.). The reaction mixture was stirred at room temperature and immediately turned yellow. The mixture was stirred until a white precipitate was formed and the solution turned colorless (at this point TLC showed complete conversion to the product). Then the suspension aged for 12 h at room temperature without being stirred. Afterwards, the precipitate was filtered off and washed with cold CF_3CH_2OH (70 mL). To the filtrate saturated aqueous NH_4Cl (100 mL) was added and the organic solvent was removed under reduced pressure. The aqueous slurry was diluted with H_2O (50 mL) and then extracted with ethyl acetate (500 mL). The combined organic layers were dried over Na_2SO_4, filtered, and concentrated under reduced pressure to obtain **SI-13** as a colorless foam (5.40 g, 11.2 mmol, 90%).

^{1}H-NMR ($CDCl_3$, 400 MHz): 6.92 (s, 1H, Im-*H*), 5.26 (bs, 1H) 5.13 (d, 1H, *J* = 3.7 Hz, OTIPS-C*H*), 3.95–3.78 (m, 2H, oxazolidine-C*H*$_2$), 3.67–3.54 (m, 1H, oxazolidine-C*H*),1.48 (s, 9H, Boc), 1.25 (m, 6H, acetonide-Me$_2$), 1.03–0.95 (m, 21H, TIPS); **^{13}C{1H}-NMR** ($CDCl_3$, 100 MHz): 152.9 (Boc-*C*O), 143.9 (Im-*C*4),132.4 (Im-*C*2), 127.8 (Im-*C*5), 94.1 (acetonide-*C*$_q$), 81.0 (Boc-*C*$_q$), 68.2 (*C*HOTIPS), 65.5 (oxazolidine-*C*H$_2$), 61.9 (oxazolidine-*C*H), 28.2 (Boc), 27.5 (acetonide-Me1), 24.6 (acetonide-Me2), 18.0 (TIPS-Me), 17.9 (TIPS-Me), 12.5 (TIPS-*C*H). **HRMS (ESI)** m/z $[M+H]^+$ calcd. for $C_{23}H_{43}ClN_3O_4Si$ 488.2706; found: 488.2705; **R$_f$** (petroleum ether/ethyl acetate 3:1): 0.35; **Specific rotation** $[\alpha]_D^{25.6}$ = +41.1 (c = 1.00; $CHCl_3$).

***tert*-Butyl (*R*)-4-((*S*)-(2-chloro-1-(*N*,*N*-dimethylsulfamoyl)-1*H*-imidazol-4-yl)((triisopropylsilyl)oxy)methyl)-2,2-dimethyloxazolidine-3-carboxylate (39):**

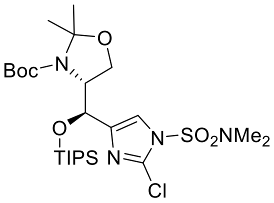

39

To a solution of **SI-13** (5.40 g, 11.2 mmol, 1.00 eq.) in CH_2Cl_2 at 0 °C was added NaH (292 mg, 12.2 mmol, 1.10 eq.) under argon atmosphere. The suspension was stirred at 0 °C for 5 min and was then allowed to warm to room temperature for 25 min. Afterwards, the suspension was cooled to 0 °C and *N,N*-dimethylsulfamoyl chloride (1.40 mL, 13.3 mmol, 1.20 eq.) was added to the solution. The reaction mixture was stirred at room temperature for 12 h. Then saturated aqueous NH_4Cl (150 mL) was added, the organic layer was separated and the aqueous layer was extracted with ethyl acetate (3 × 130 mL). The combined organic layers were dried over Na_2SO_4, filtered, and the solvent was removed under reduced pressure. The resulting crude product was purified *via* column chromatography (silica, petroleum ether : ethyl acetate 5:1) to obtain **39** (6.30 g, 10.5 mmol, 95%) as a colorless oil.

^{1}H-NMR ($CDCl_3$, 400 MHz): **Major rotamer**: 7.27 (s, 1H, Im), 5.08 (m, 1H, CHOTIPS), 4.37 (dd, 1H, *J* = 8.9, 2.4 Hz, oxazolidine-C*H*$_2$), 4.04–3.98 (m, 1H, oxazolidine-CH), 3.90–3.84 (m, 1H, oxazolidine-C*H*$_2$), 2.98 (s, 6H, NMe$_2$), 1.60 (s, 3H, acetonide-Me1), 1.46 (s, 3H, acetonide-Me2), 1.42 (s, 9H, Boc), 1.05–0.98 (m, 21H, TIPS); **^{13}C{1H}-NMR** ($CDCl_3$, 100 MHz): 152.9 (s, Boc-*C*O), 142.9 (s, Im-*C*4), 129.2 (s, Im-*C*2), 119.8 (d, Im-*C*5), 94.2 (s, acetonide-*C*$_q$), 80.1 (s, Boc-*C*$_q$), 68.5 (d, *C*HOTIPS), 63.7 (t, oxazolidine-*C*H$_2$), 63.2 (d, oxazolidine-*C*H), 38.5 (q, NMe$_2$), 28.5 (q, Boc), 27.3 (q, acetonide-Me1), 24.9 (q, acetonide-Me2), 18.1 (q, TIPS-Me), 18.0 (q, TIPS-Me), 12.8 (d, TIPS-*C*H); **^{1}H-NMR** ($CDCl_3$, 400 MHz): **Minor rotamer** 7.20 (s, 1H, Im), 4.98 (m, 1H, CHOTIPS), 4.37 (dd, 1H, *J* = 8.9, 2.4 Hz, oxazolidine-C*H*$_2$), 4.04–3.98 (m, 1H, oxazolidine-CH), 3.90–3.84 (m, 1H, oxazolidine-C*H*$_2$), 2.96 (s, 6H, NMe$_2$), 1.63 (s, 3H, acetonide-Me1), 1.48 (s, 3H, acetonide-Me2), 1.43 (s, 9H, Boc), 1.05–0.98 (m, 21H, TIPS); **^{13}C{1H}-NMR** ($CDCl_3$, 100 MHz): 152.5 (s, Boc-*C*O), 142.6 (s, Im-*C*4), 129.9 (s, Im-*C*2), 119.5 (d, Im-*C*5), 94.8 (s, acetonide-*C*$_q$), 80.1 (s, Boc-*C*$_q$), 69.1 (d, *C*HOTIPS), 63.7 (t, oxazolidine-*C*H$_2$), 62.2 (d, oxazolidine-*C*H), 38.5 (q, NMe$_2$), 28.5 (q, Boc), 26.8 (q, acetonide-Me1), 23.5 (q, acetonide-Me2), 18.1 (q, TIPS-Me), 18.0 (q, TIPS-Me), 12.5 (d, TIPS-*C*H). **HRMS (ESI)** m/z $[M+H]^+$ calcd. for $C_{25}H_{48}ClN_4O_6Si$ 595.2747; found: 595.2750; **R$_f$** (petroleum ether/ethyl acetate 5:1): 0.3; **Specific rotation** $[\alpha]_D^{25.6}$ = +30.2 (c = 1.06; $CHCl_3$).

***tert*-Butyl ((1*S*,2*R*)-1-(2-chloro-1-(*N*,*N*-dimethylsulfamoyl)-1*H*-imidazol-4-yl)-3-hydroxy-1-((triisopropylsilyl)oxy) propan-2-yl)carbamate (SI-14):**

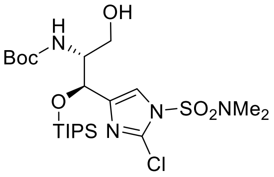

SI-14

Under argon atmosphere, **SI-39** (6.30 g, 10.5 mmol, 1.00 eq.) was dissolved in a pre-cooled mixture of aqueous 1 M HCl/AcOH (2:5, 25 mL, 1:9) at 4 °C. The reaction mixture was stirred until completion was monitored *via* TLC (usually within 2-3 h). Then, Na_2CO_3 was added slowly to the mixture until no further gas formation was observed. Saturated aqueous $NaHCO_3$ (100 mL) was added to the slurry, and the aqueous layer was extracted with ethyl acetate (3 × 120 mL). The combined organic layers were dried over Na_2SO_4, filtered, and the solvent was removed under reduced pressure. The resulting crude product was purified *via* short plug column chromatography (silica, petroleum ether : ethyl acetate 3 :1) to obtain **SI-14** (4.70 g, 8.41 mmol, 80%) as a colorless solid.

^{1}H-NMR ($CDCl_3$, 400 MHz): 7.31 (s, 1H, Im-*H*), 6.93 (bs, 1H, N*H*), 5.29–5.20 (bs, 1H, O*H*), 5.14–5.06 (m, 1H, TIPSO-C*H*), 3.94–3.84 (m, 2H, CH_2-OH), 3.61–3.51 (m, 1H, NHBoc-C*H*), 2.99 (s, 6H, NMe_2), 1.43 (s, 9H, Boc), 1.06–0.98 (m, 21H, TIPS); **^{13}C-NMR** ($CDCl_3$, 100 MHz): 156.0 (s, Boc-*C*O), 141.4 (s, Im-*C*4), 130.1 (s, Im-*C*2), 120.1 (d, Im-*C*5), 79.7 (s, Boc-C_q), 71.2 (d, *C*1), 62.5 (t, *C*3), 56.4 (d, *C*2), 38.6 (q, NMe_2), 28.5 (q, Boc), 18.1 (q, TIPS-Me), 18.1 (q, TIPS-Me), 12.4 (d, TIPS-CH); **HRMS (ESI)** m/z $[M+Na]^+$ calcd. for $C_{22}H_{43}ClN_4O_6SSiNa$ 577.2253; found: 577.2238; **R_f** (petroleum ether/ethyl acetate 3:1): 0.35; **Specific rotation** $[\alpha]_D^{25.6}$ = +56.7 (c = 1.02; $CHCl_3$).

Methyl (2*S*,3*S*)-2-((*tert*-butoxycarbonyl)amino)-3-(2-chloro-1-(*N*,*N*-dimethylsulfamoyl)-1*H*-imidazol-4-yl)-3-((triisopropylsilyl)oxy)propanoate (SI-16):

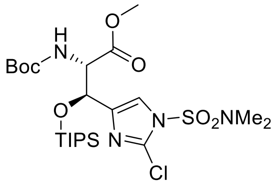

SI-16

To a stirred solution of primary alcohol **SI-14** (120 mg, 0.210 mmol 1.00 eq.) in MeCN (1 mL) a catalytic amount of TEMPO (2 mg, 0.01 mmol; 0.05 eq.) was added at room temperature. Then simultaneously, a solution of $NaClO_2$ (117 mg, 1.29 mmol, 6.00 eq.) in a pH 4.0 phosphate buffer (1.5 mL) and aqueous NaOCl (2.3 M, 91 µL, 0.21 mmol, 1.0 eq.) were added dropwise to the solution. The reaction mixture turned dark red immediately and was stirred at room temperature for 12 h. Afterwards, the reaction was diluted with saturated aqueous Na_2SO_3 (2 mL) and the reaction turned a pale yellow. The mixture was stirred for 20 min until a colorless solution was formed. Then MeCN was removed under reduced pressure and to the resulting slurry CH_2Cl_2 (1 mL) was added. The biphasic system was acidified with aqueous $KHSO_4$ (1 M, 5 mL) to pH 2 and the mixture was extracted with ethyl acetate (3 × 30 mL). The combined organic layers were dried over Na_2SO_4, filtered, and the solvent was removed under reduced pressure. The free carboxylic acid **SI-15** (123 mg, 0.210 mmol, quant.) was obtained as a colorless foam and was used as a crude product without further purification

Carboxylic acid **SI-15** (123 mg, 0.210 mmol, 1.00 eq.) was dissolved in a mixture of anhydrous toluene and MeOH (2 mL, 3:2) and $TMSCH_2N_2$ (2 M in Et_2O, 119 µL, 0.230 mmol, 1.10 eq.) was added. The reaction was stirred at room temperature for 5 min. The solvent was removed under reduced pressure. The resulting crude product was purified *via* short plug column chromatography (silica, petroleum ether/ethyl acetate 5:1) to obtain **SI-16** (76 mg, 0.13 mmol, 60% over two steps) as a colorless solid.

^{1}H-NMR ($CDCl_3$, 400 MHz): 7.27 (s, 1H, Im-*H*),), 5.40–5.35 (m, 1H, OTIPS-C*H*), 4.5 (d, 1H, *J* = 8.6 Hz, NH-CH_2), 3.73 (s, 3H, CO_2Me), 3.67–3.58 (m, 1H, oxazolidine-C*H*), 2.96 (s, 6H, NMe_2), 1.42 (m, 9H, Boc), 1.02–0.98 (m, 21H, TIPS); **^{13}C{1H}-NMR** ($CDCl_3$, 100 MHz): 170.8 (*C*O_2Me), 155.7 (Boc-*C*O), 141.3 (Im-*C*4), 129.3 (Im-*C*2), 119.9 (Im-*C*5), 80.0 (Boc-C_q), 70.5 (C_β), 58.7 (C_α), 52.3 (OMe), 39.4 (NMe_2), 28.3 (Boc), 17.9 (TIPS-Me), 17.8 (TIPS-Me), 12.4 (TIPS-*C*H); **HRMS (ESI)** m/z $[M+Na]^+$ calcd. for $C_{23}H_{43}ClN_4O_7SSiNa$ 605.2202; found: 605.2204; **R_f** (petroleum ether/ethyl acetate 4:1): 0.35; **Specific rotation** $[\alpha]_D^{25.6}$ = +87.7 (c = 1.07; $CHCl_3$).

2-(Trimethylsilyl)ethyl (2*S*,3*S*)-2-((*tert*-butoxycarbonyl)amino)-3-(2-chloro-1-(*N*,*N*-dimethylsulfamoyl)-1*H*-imidazol-4-yl)-3-((triisopropylsilyl)oxy)propanoate (SI-17):

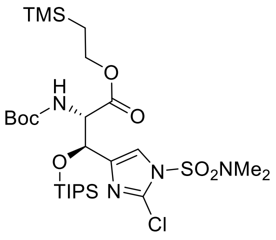

SI-17

To a stirred solution of primary alcohol **SI-14** (4.70 g, 8.41 mmol 1.00 eq.) in MeCN (8 mL) a catalytic amount of TEMPO (7 mg, 0.04 mmol, 0.05 eq.) was added at room temperature. Then simultaneously, a solution of $NaClO_2$ (4.60 g; 50.4 mmol, 6.00 eq.) in a pH 4.0 phosphate buffer (10 mL) and aqueous NaOCl (2.3 M, 3.70 mL, 8.41 mmol, 1.00 eq.) were added dropwise to the solution. The reaction mixture turned dark red immediately and was stirred at room temperature for 12 h. Afterwards, the reaction was diluted with saturated aqueous Na_2SO_3 (15 mL) and the reaction turned a pale yellow. The mixture was stirred for 20 min until a colorless solution was formed. Then MeCN was removed under reduced pressure and to the resulting slurry CH_2Cl_2 (10 mL) was added. The biphasic system was acidified with aqueous 1 M $KHSO_4$ to pH 2 and the mixture was extracted with ethyl acetate (3 × 100 mL). The combined organic layers were dried

over Na_2SO_4, filtered, and the solvent was removed under reduced pressure. The free carboxylic acid **SI-15** (4.80 g, 8.41 mmol, quant.) was obtained as a colorless foam and was used as a crude product without further purification.

To a stirred solution of carboxylic acid **SI-15** (0.16 g, 0.27 mmol 1.0 eq.) in CH_2Cl_2 (0.1 M, 3 mL) at 0 °C a catalytic amount of DMAP (1.7 mg, 0.01 mmol, 0.05 eq.) and DIC (46 µL, 0.30 mmol, 1.1 eq.) were added. Then $Me_3SiCH_2CH_2OH$ (47 µL, 0.33 mmol, 1.2 eq.) was added and the reaction mixture was allowed to warm to room temperature and stirred for 12 h. Afterwards saturated aqueous NH_4Cl (10 mL) was added and the layers were separated. The aqueous layer was extracted with ethyl acetate (3 × 20 mL). The combined organic layers were washed with saturated aqueous NaCl (50 mL), dried over Na_2SO_4, filtered, and the solvent was removed under reduced pressure. The resulting crude product was purified *via* column chromatography (silica, petroleum ether/ethyl acetate 4:1) to obtain **SI-17** (0.16 g, 0.23 mmol, 85% over two steps) as a colorless solid

^{1}H-NMR ($CDCl_3$, 400 MHz): 7.26 (s, 1H, Im-*H*), 5.34 (d, 1H, *J* = 8.6 Hz, N*H*Boc) 5.27–5.21 (m, 1H, OTIPS-C*H*), 4.73–4.67 (m, 1H, NH-CH_2), 4.24–4.10 (m, 2H, CH_2-CH_2TMS), 2.96 (s, 6H, NMe_2), 1.42 (s, 9H, Boc), 1.07–1.00 (m, 21H, TIPS), 0.96 (t, 3H, *J* = 8.7 Hz CH_2TMS), 0.02 (m, 9H, TMS). **^{13}C{1H}-NMR** ($CDCl_3$, 100 MHz): 169.9 (*C*O_2Me), 155.5 (Boc-*C*O), 140.8 (Im-*C*4), 129.9 (Im-*C*2), 120.2 (Im-*C*5), 79.8 (Boc-C_q), 63.9 (TMS-CH_2-*C*H_2-), 59.4 ($C\alpha$), 38.6 (NMe_2), 28.4 (Boc), 18.1 (TIPS-Me), 18.1 (TIPS-Me), 12.4 (TMS-*C*H_2), -1.4 (TMS); **HRMS (ESI)** m/z $[M+Na]^+$ calcd. for $C_{27}H_{53}ClN_4O_7SSi_2Na$ 691.2754; found: 691.2758; **R_f** (petrolum ether/ethyl acetate 3:1): 0.35; **Specific rotation** $[\alpha]_D^{25.6}$ = +78.9 (c = 1.00; $CHCl_3$).

2-(Trimethylsilyl)ethyl (2*S*,3*S*)-2-amino-3-(2-chloro-1*H*-imidazol-4-yl)-3-((triisopropylsilyl)oxy)propanoate (44):

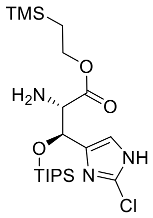

44

To a solution of **SI-18** (0.16 g, 0.23 mmol, 1.0 eq.) in THF (2 mL), a solution of HCl in 1,4-dioxane (4 M, 0.17 mL, 0.70 mmol, 3.0 eq.) was added. The resulting mixture was stirred for 12 h at room temperature. The mixture was concentrated under reduced pressure to provide crude dihydrochloride **44** (0.12 g, 0.23 mmol) as an orange solid, which was used without further purification.

HRMS (ESI) m/z $[M+H]^+$ calcd. for $C_{20}H_{41}ClN_3O_3Si_2$ 462.2369; found: 462.2378; **R_f** (*2*0% MeOH in CH_2Cl_2): 0.19.

2. NMR Spectra

^{1}H-NMR, $CDCl_3$, 600 MHz

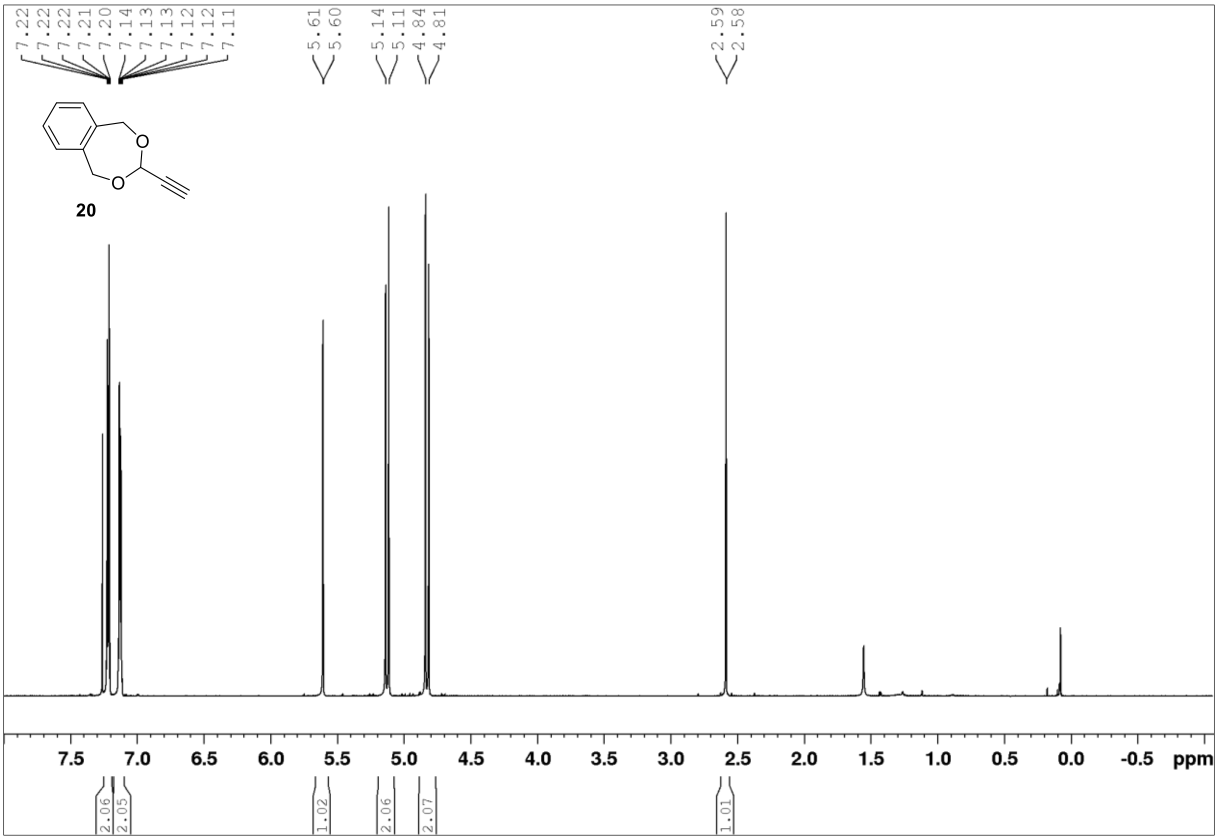

^{13}C{1H}-NMR, $CDCl_3$, 150 MHz

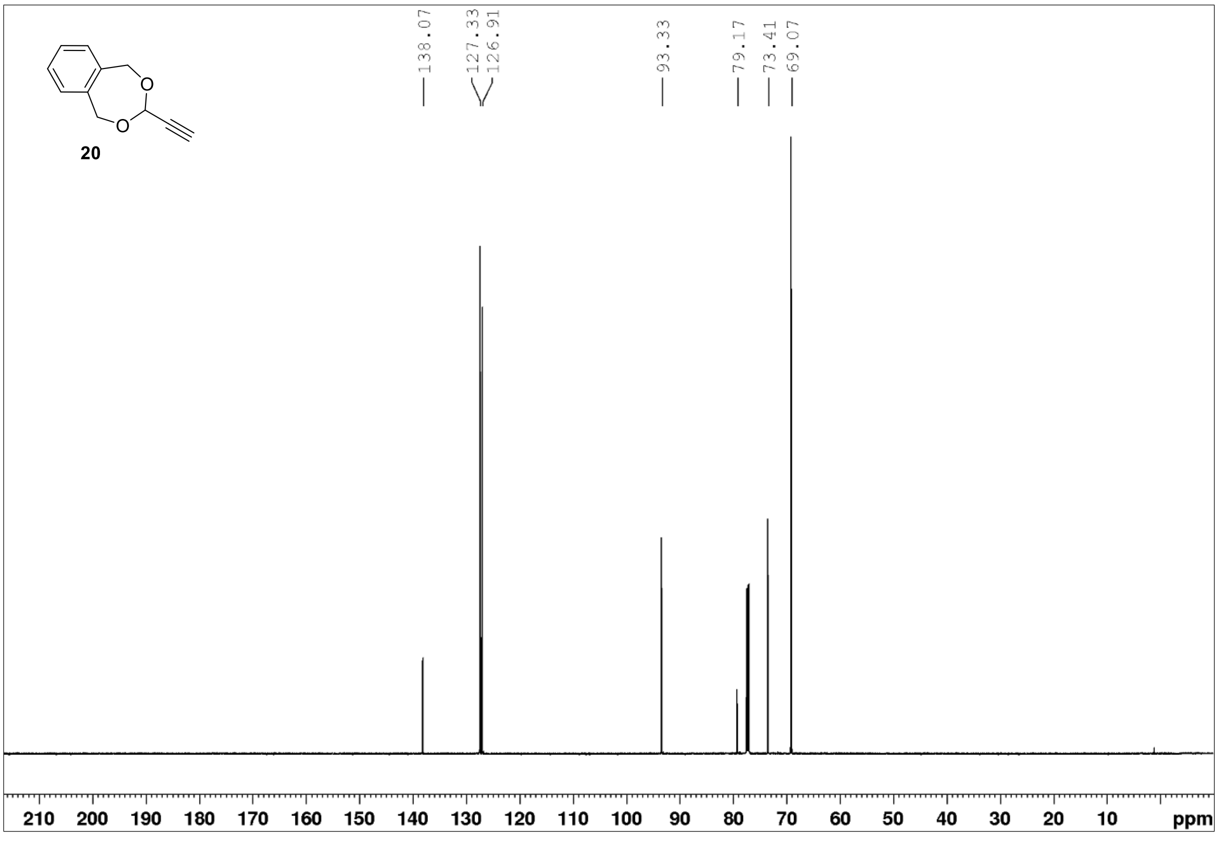

^{1}H-NMR, $CDCl_3$, 400 MHz

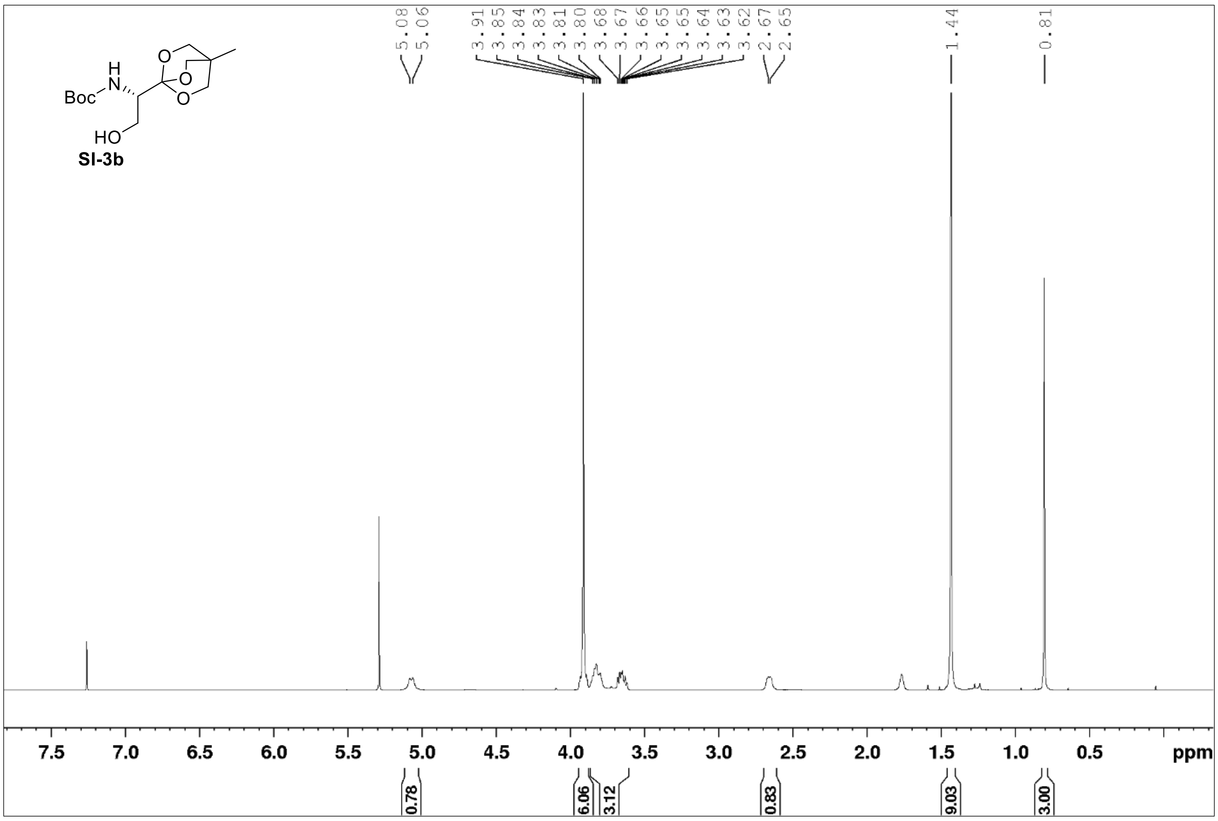

^{13}C{1H}-$CDCl_3$, 100 MHz

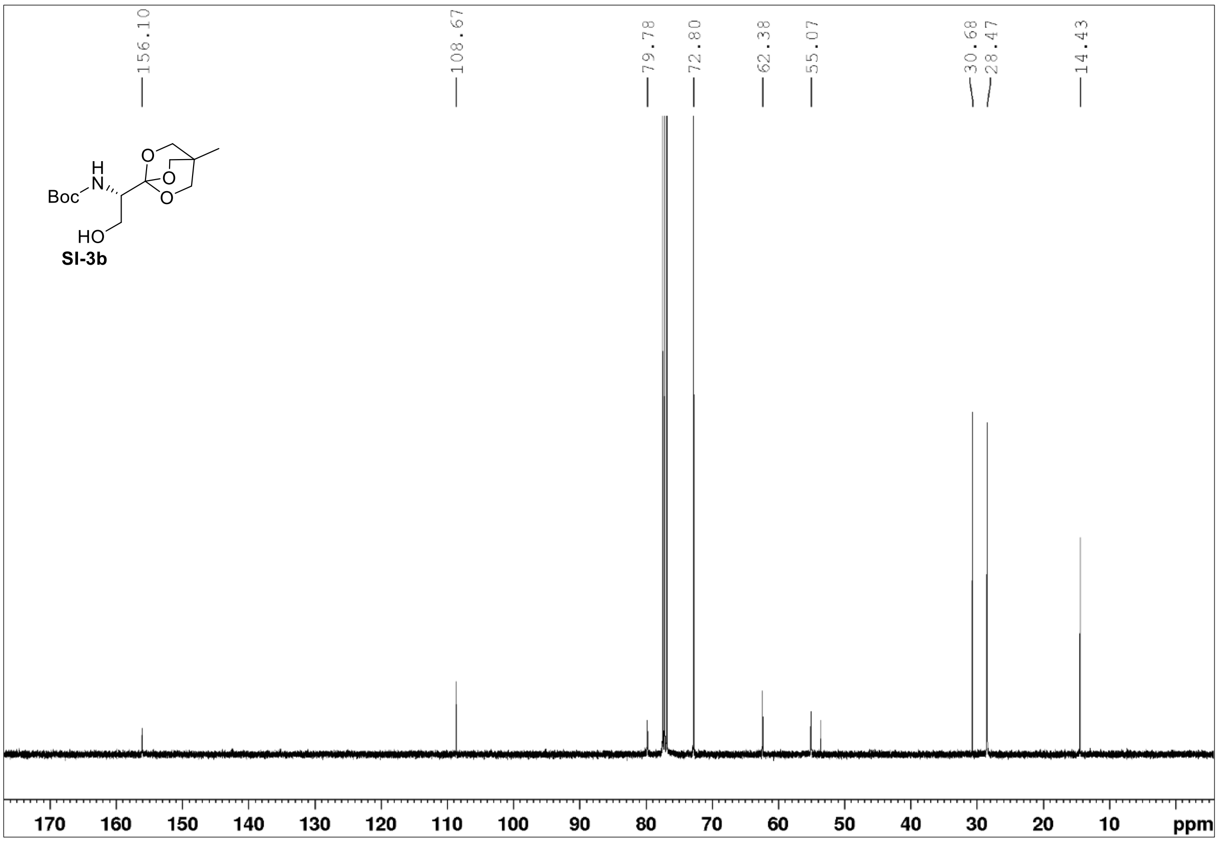

^{1}H-NMR, $CDCl_3$, 600 MHz

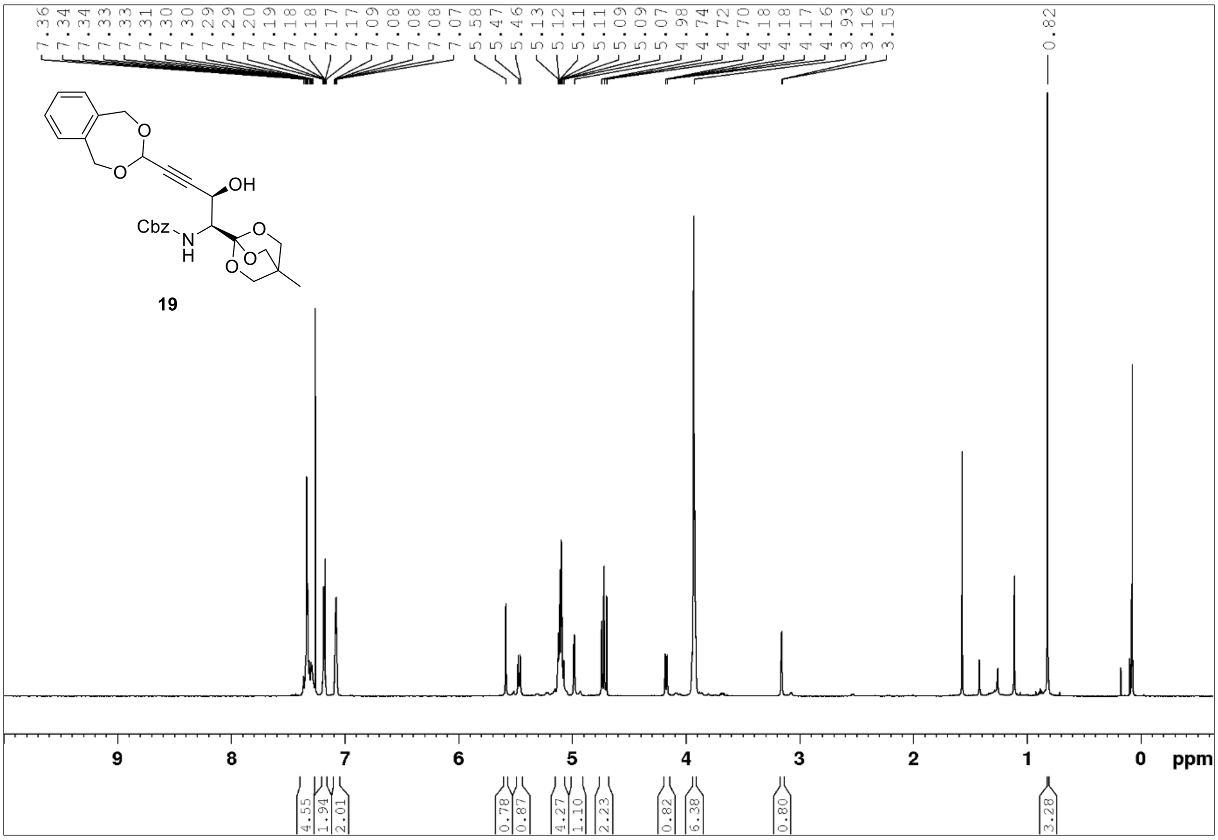

^{13}C{1H}-$CDCl_3$, 150 MHz

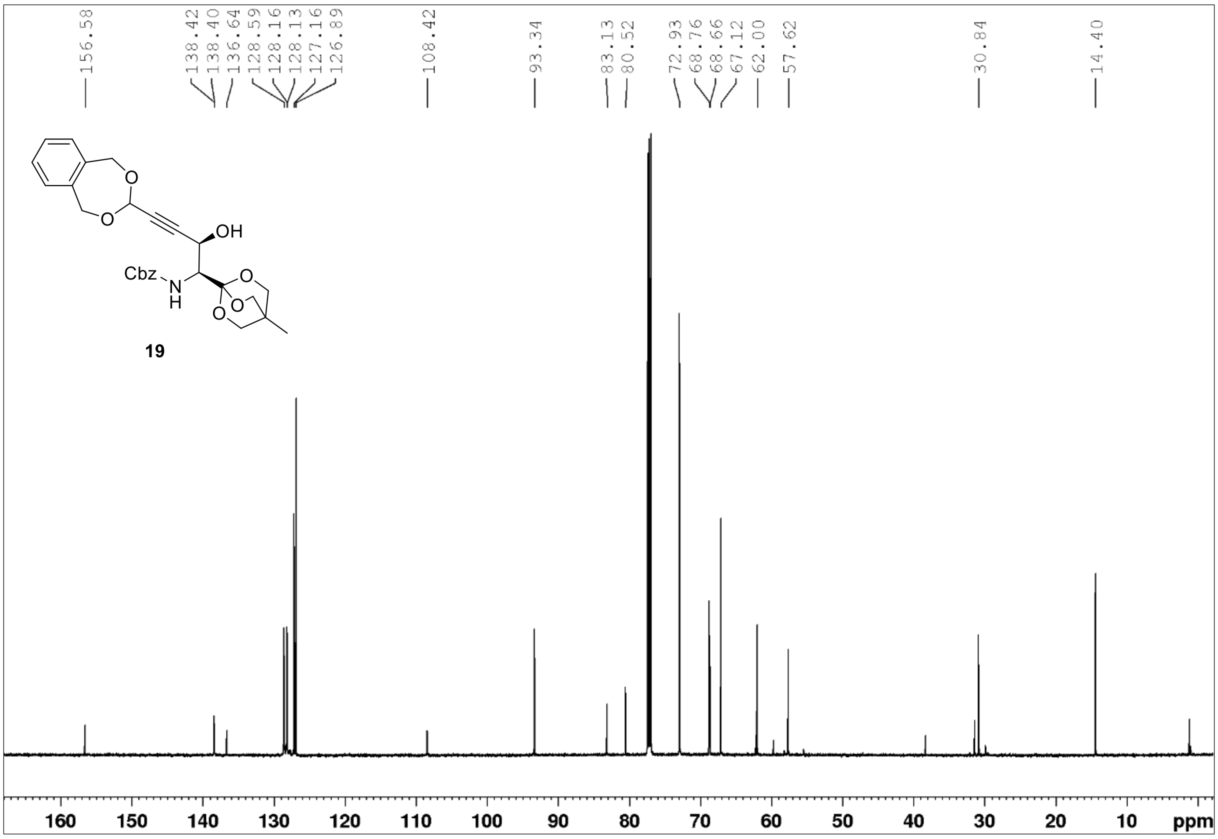

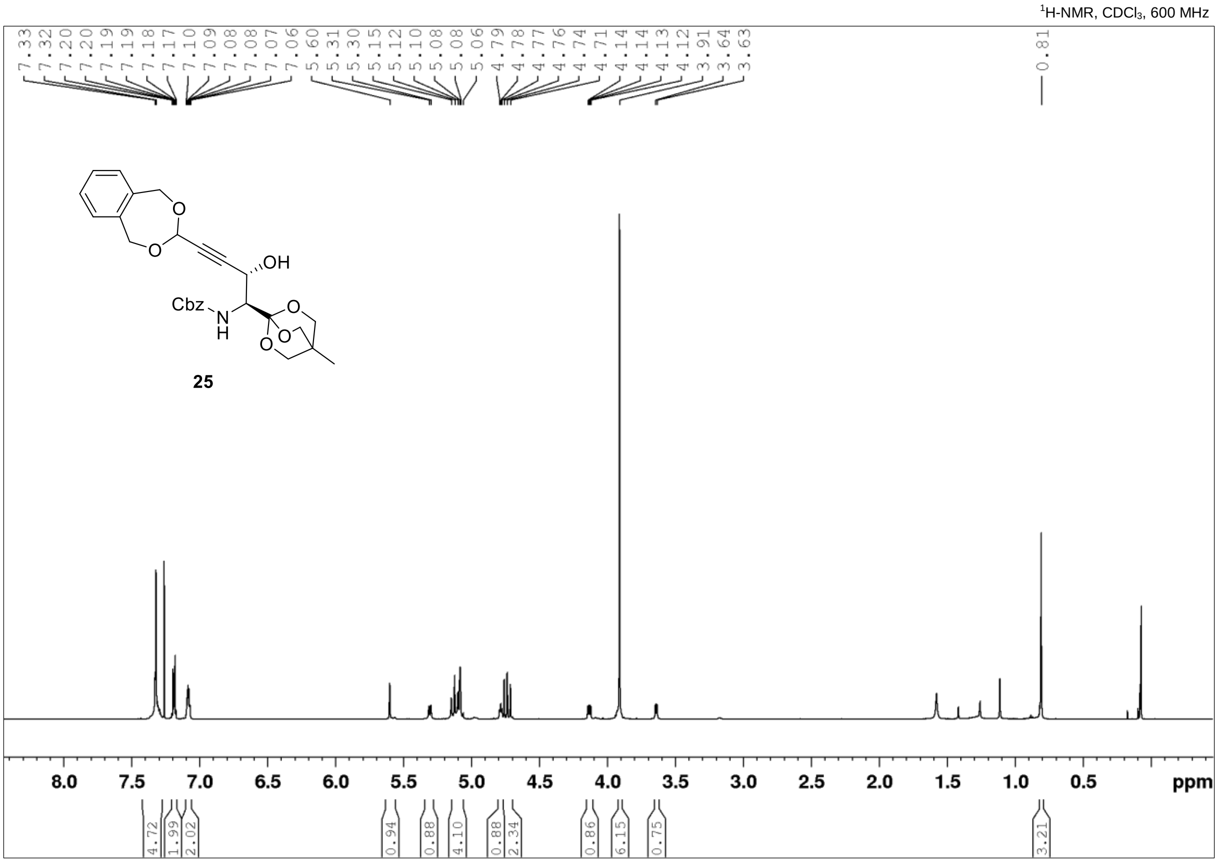
1H-NMR, CDCl3, 600 MHz
OH
Cbz
N
H
O
25
ppm

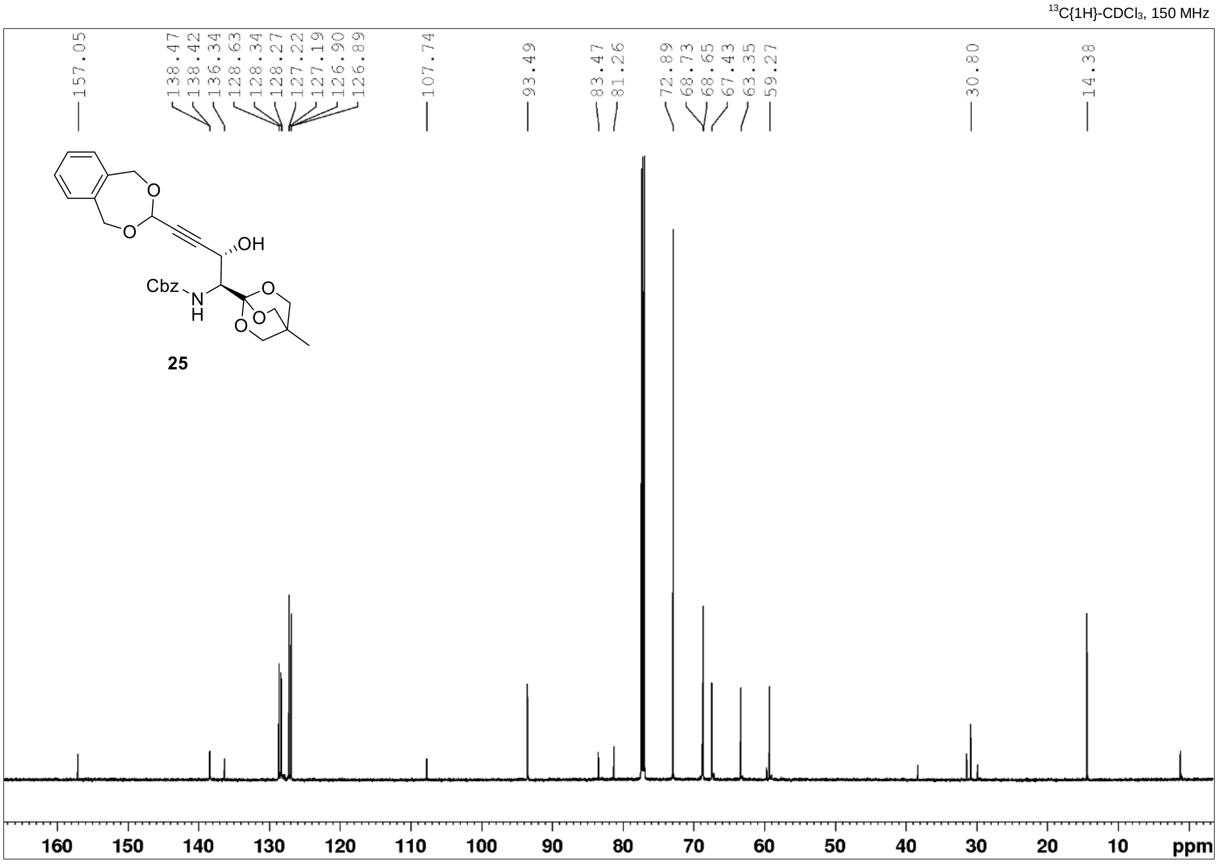
13C{1H}-CDCl3, 150 MHz
OH
Cbz
N
H
O
25
ppm

Mixture of compound **19** and **25**, prepared by the deprotonation of alkyne **20** with *n*-BuLi: *dr* > 20:1 (determined by ^{1}H-NMR)

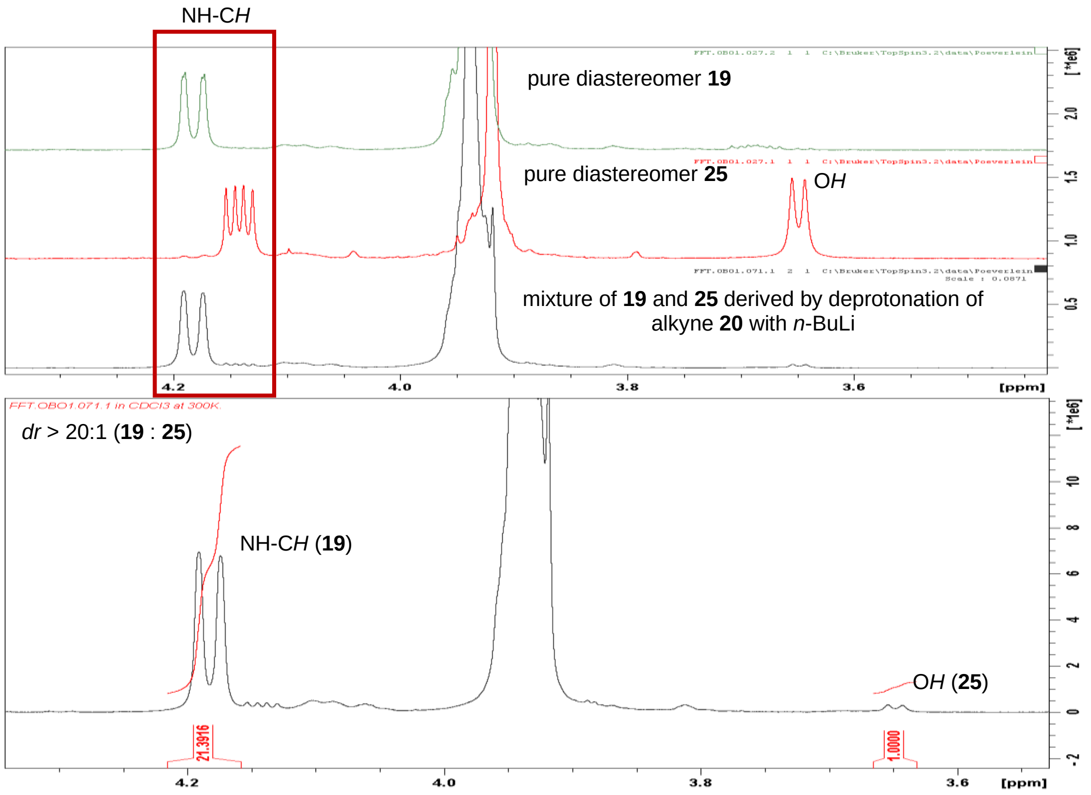

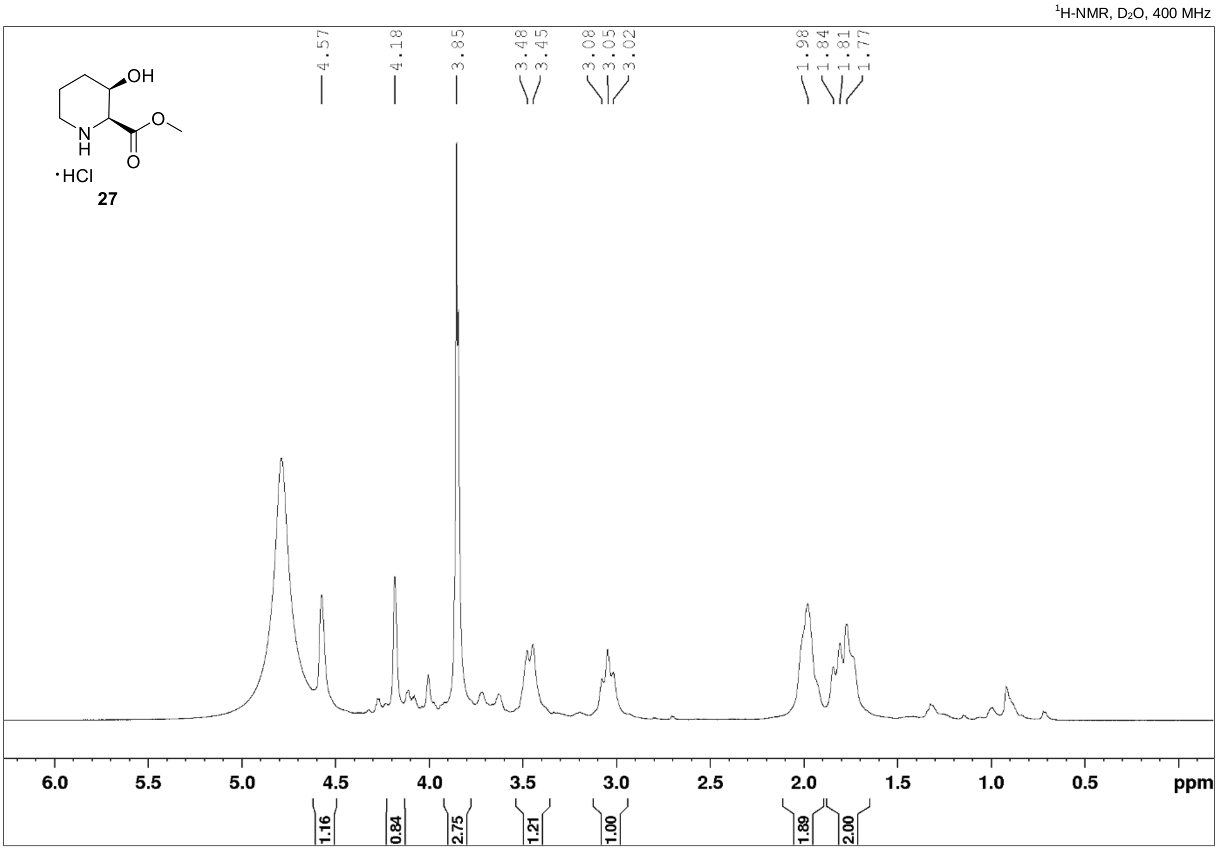
^{1}H-NMR, D$_2$O, 400 MHz
OH
N
H
O
O
•HCl
27
4.57
4.18
3.85
3.48
3.45
3.08
3.05
3.02
1.98
1.84
1.81
1.77
6.0
5.5
5.0
4.5
4.0
3.5
3.0
2.5
2.0
1.5
1.0
0.5
ppm
1.16
0.84
2.75
1.21
1.00
1.89
2.00

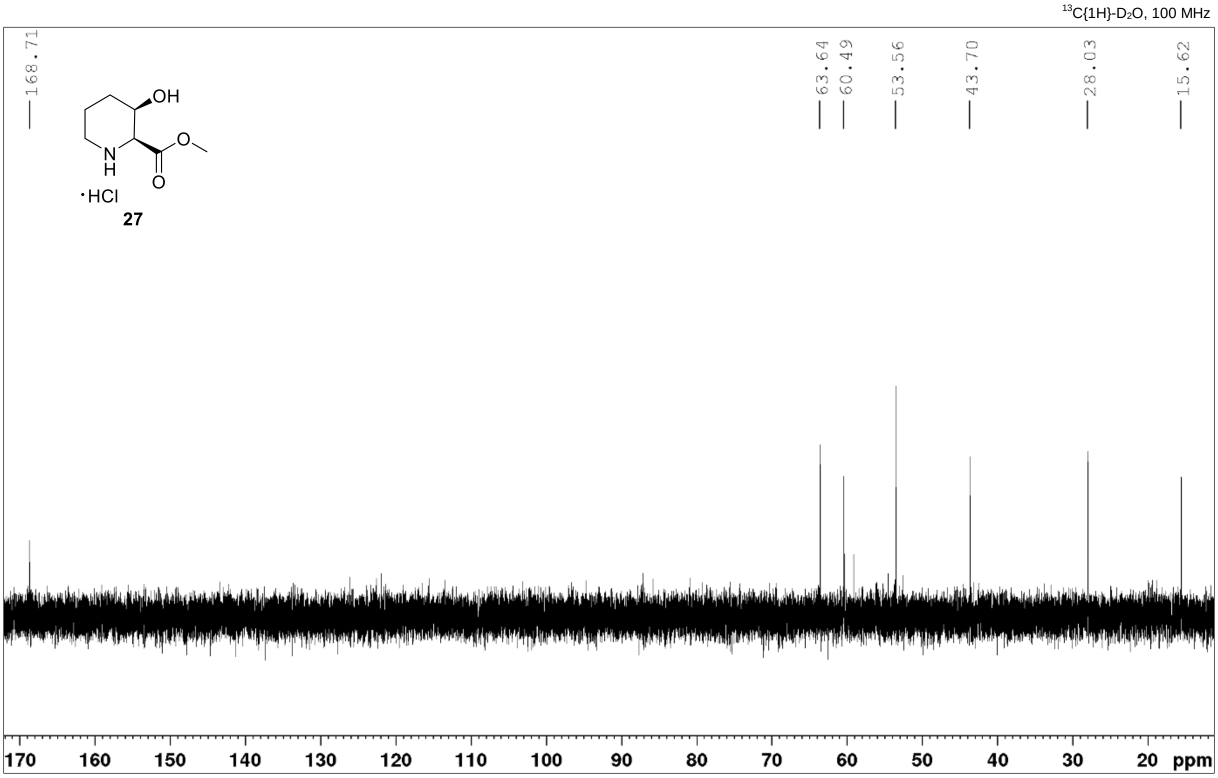
^{13}C{1H}-D$_2$O, 100 MHz
168.71
OH
N
H
O
O
•HCl
27
63.64
60.49
53.56
43.70
28.03
15.62
170
160
150
140
130
120
110
100
90
80
70
60
50
40
30
20
ppm

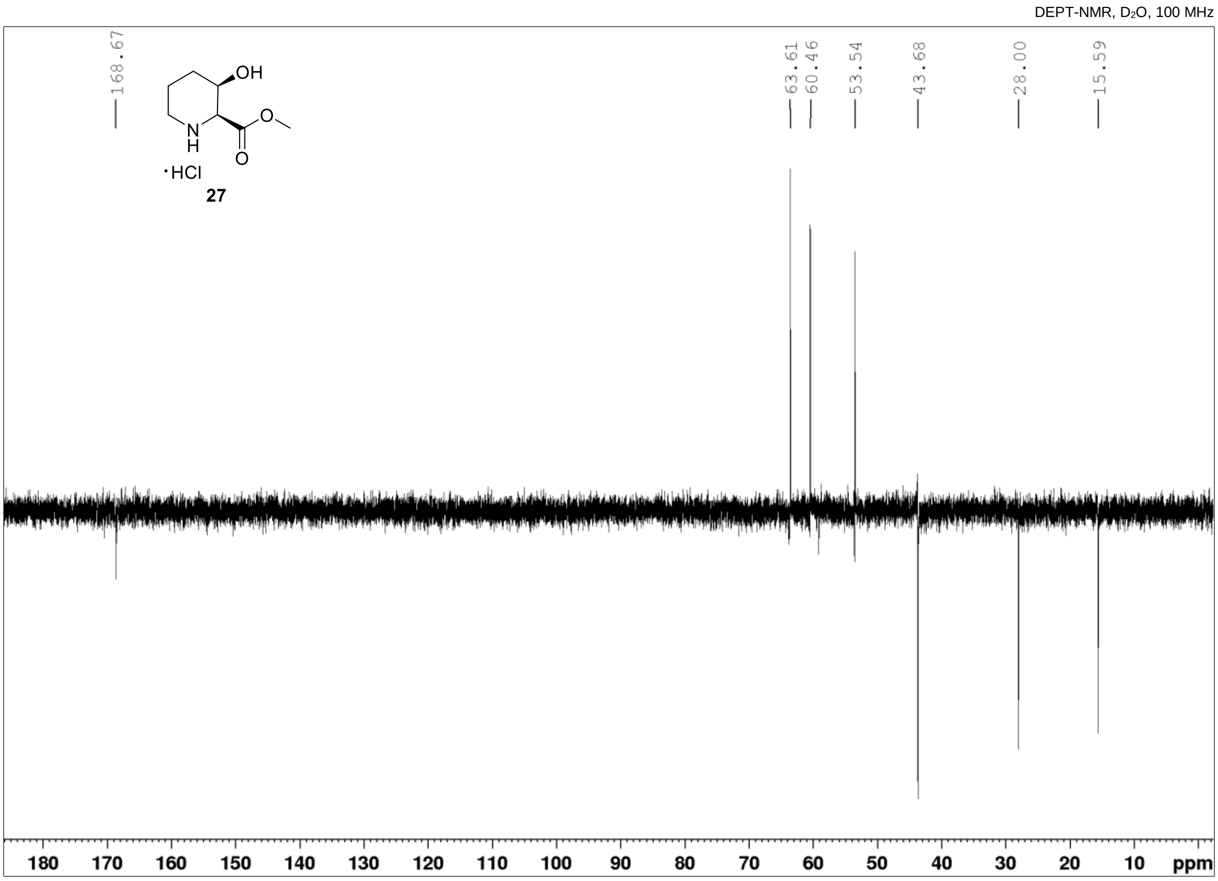
DEPT-NMR, D2O, 100 MHz
168.67
63.61
60.46
53.54
43.68
28.00
15.59
OH
N
H
O
O
·HCl
27
180 170 160 150 140 130 120 110 100 90 80 70 60 50 40 30 20 10 ppm

^{1}H-NMR, $CDCl_3$, 400 MHz

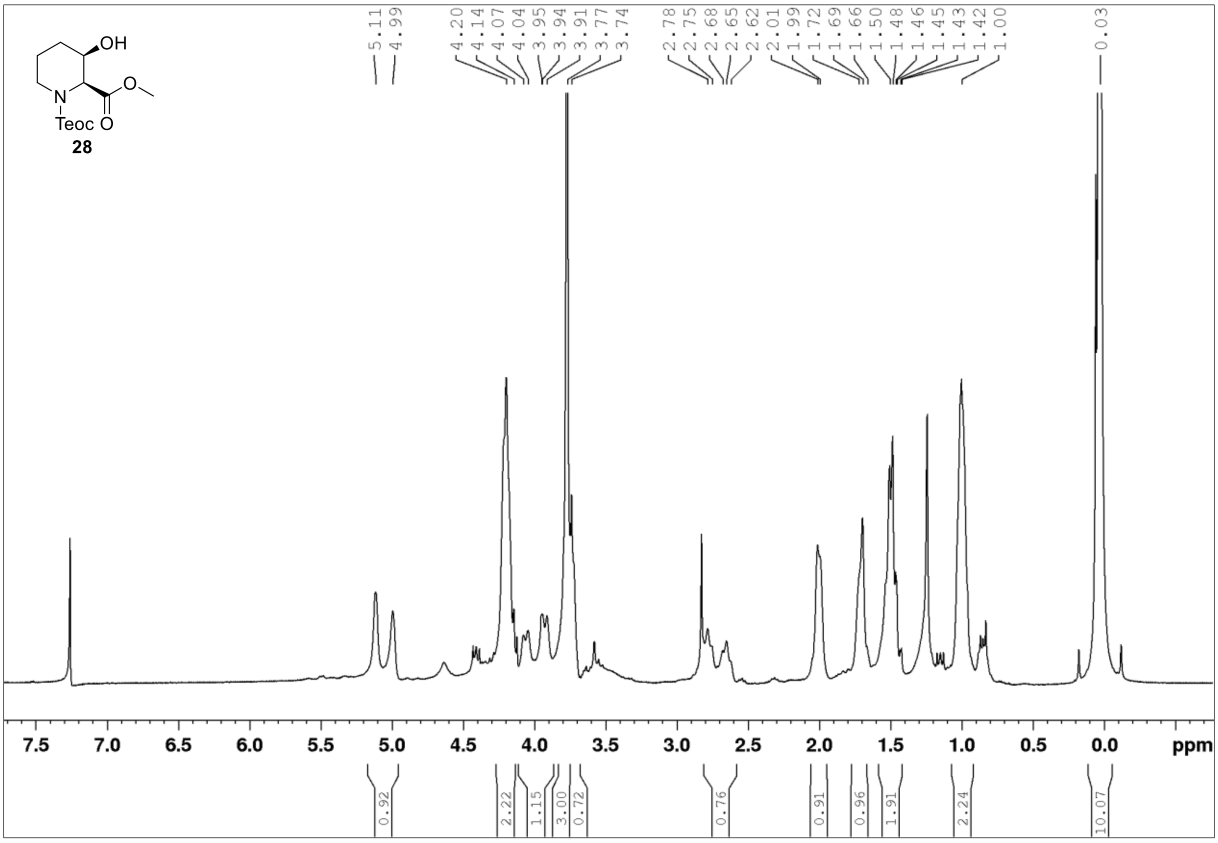

^{13}C{1H}-$CDCl_3$, 100 MHz

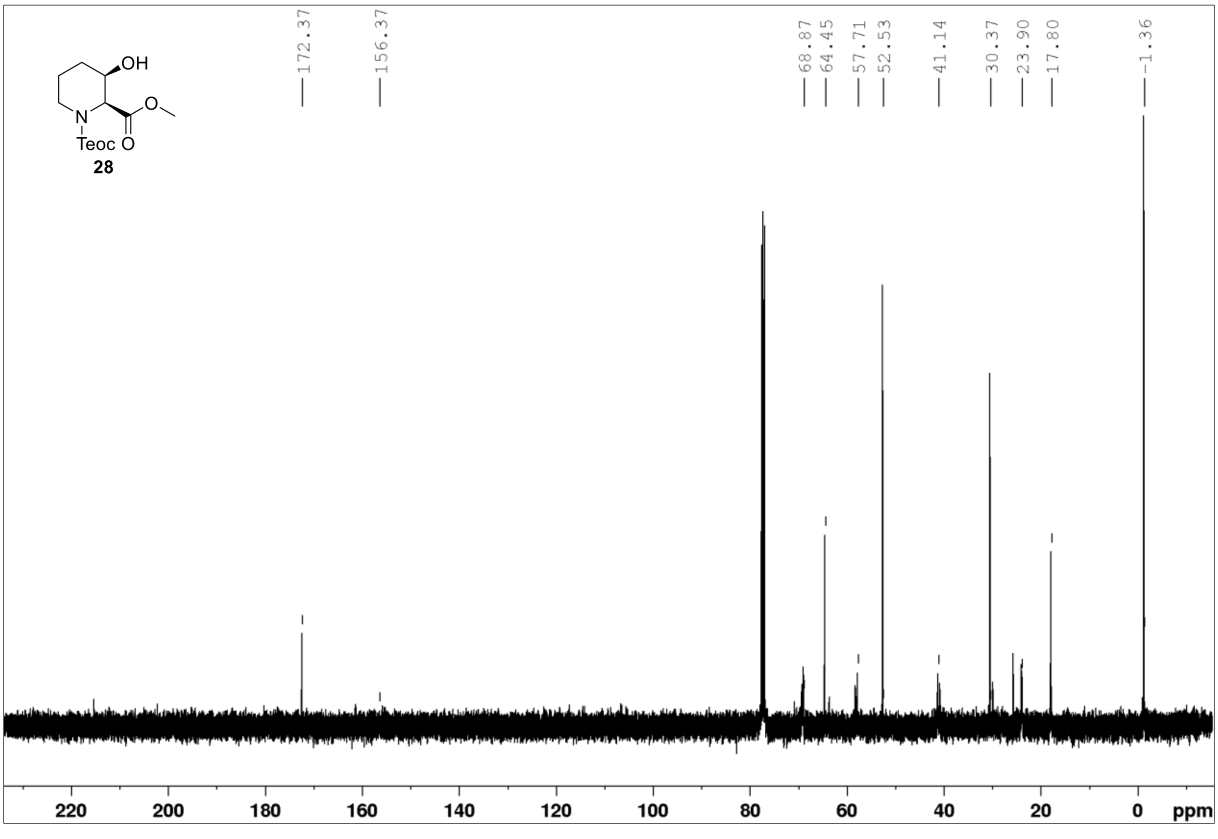

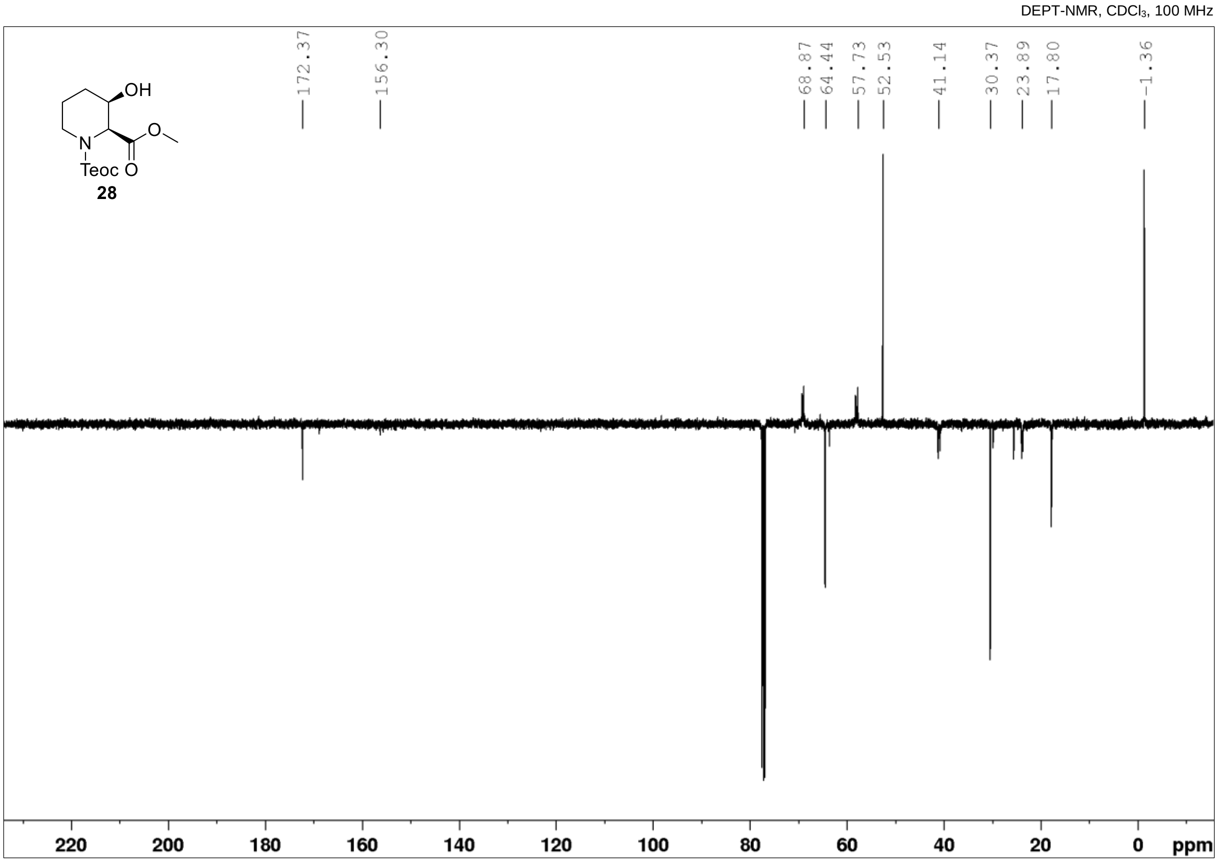
DEPT-NMR, CDCl3, 100 MHz
172.37
156.30
68.87
64.44
57.73
52.53
41.14
30.37
23.89
17.80
-1.36
OH
N
O
Teoc O
28
220
200
180
160
140
120
100
80
60
40
20
0
ppm

^{1}H-NMR, C_6D_6, 400 MHz

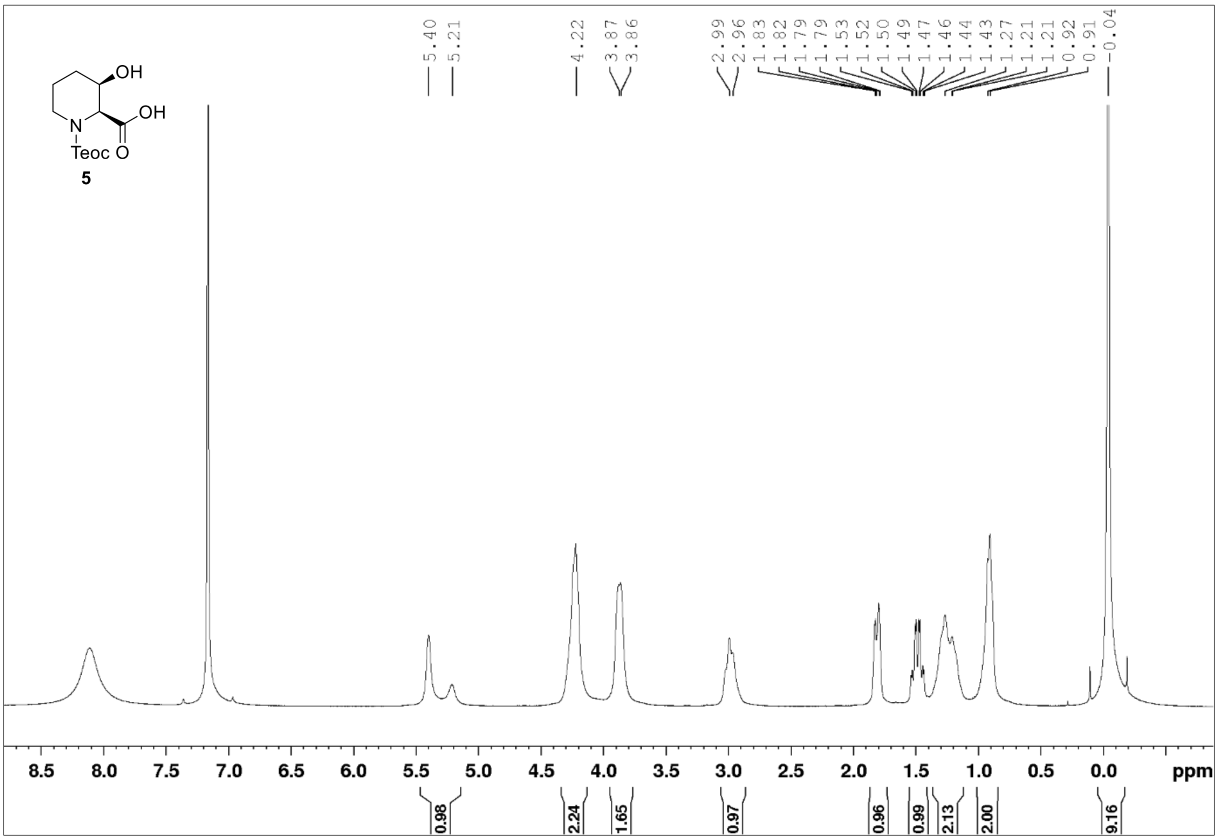

^{13}C{1H}-C_6D_6, 100 MHz

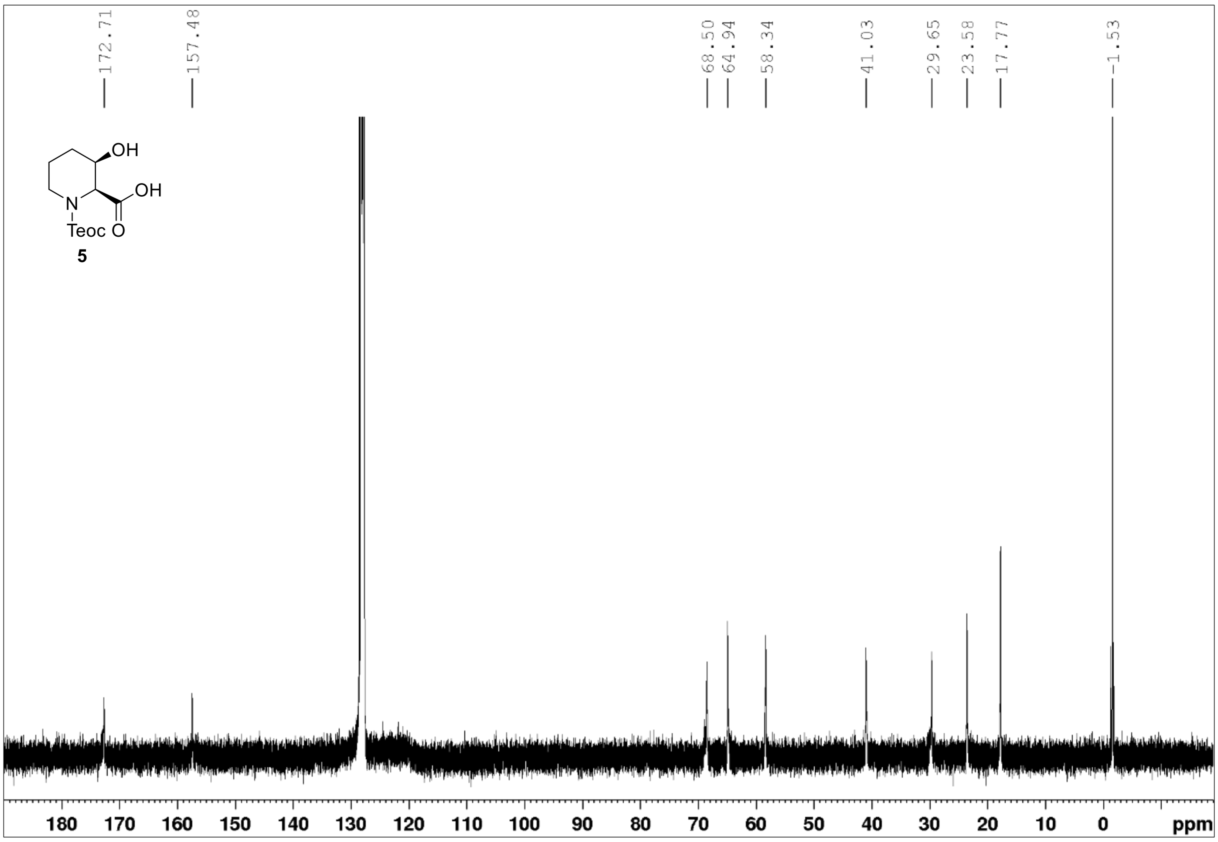

^{1}H-NMR, $CDCl_3$, 400 MHz

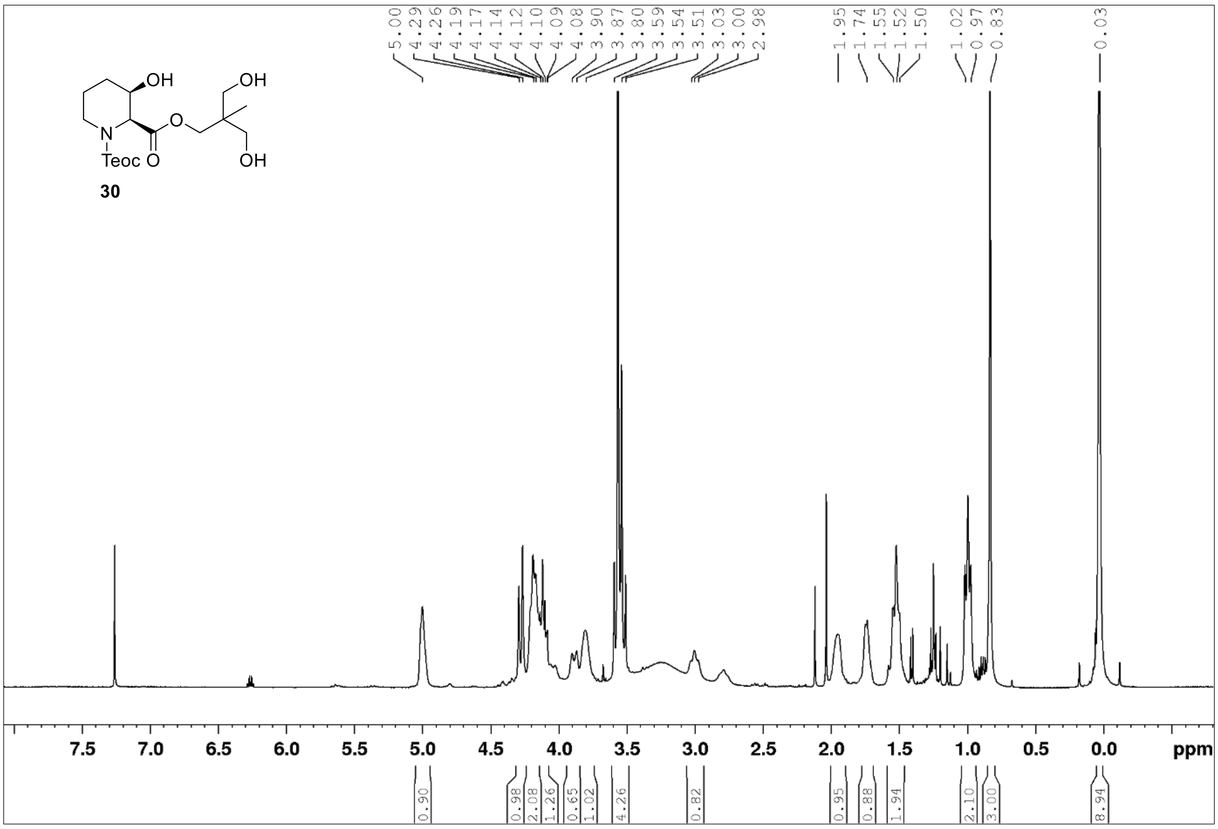

^{13}C{1H}-$CDCl_3$, 100 MHz

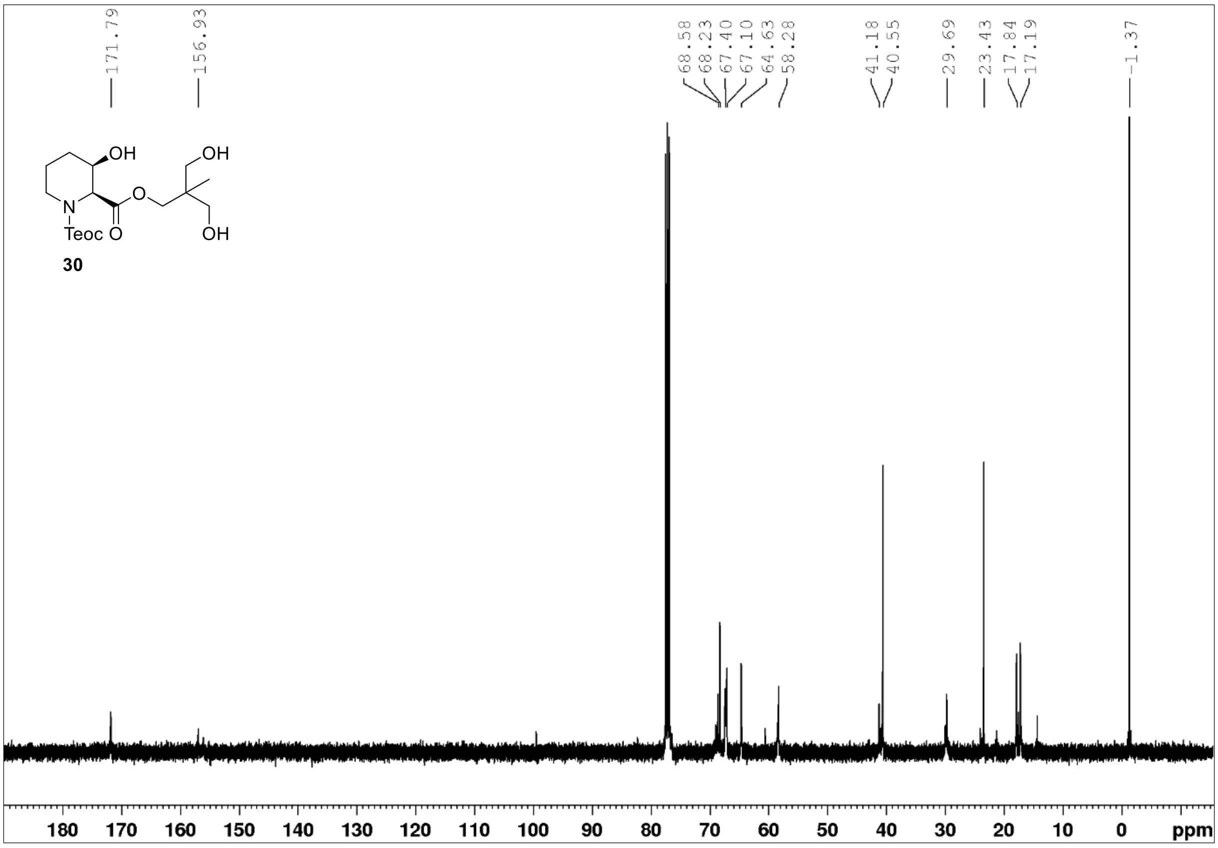

^{1}H-NMR, $CDCl_3$, 400 MHz

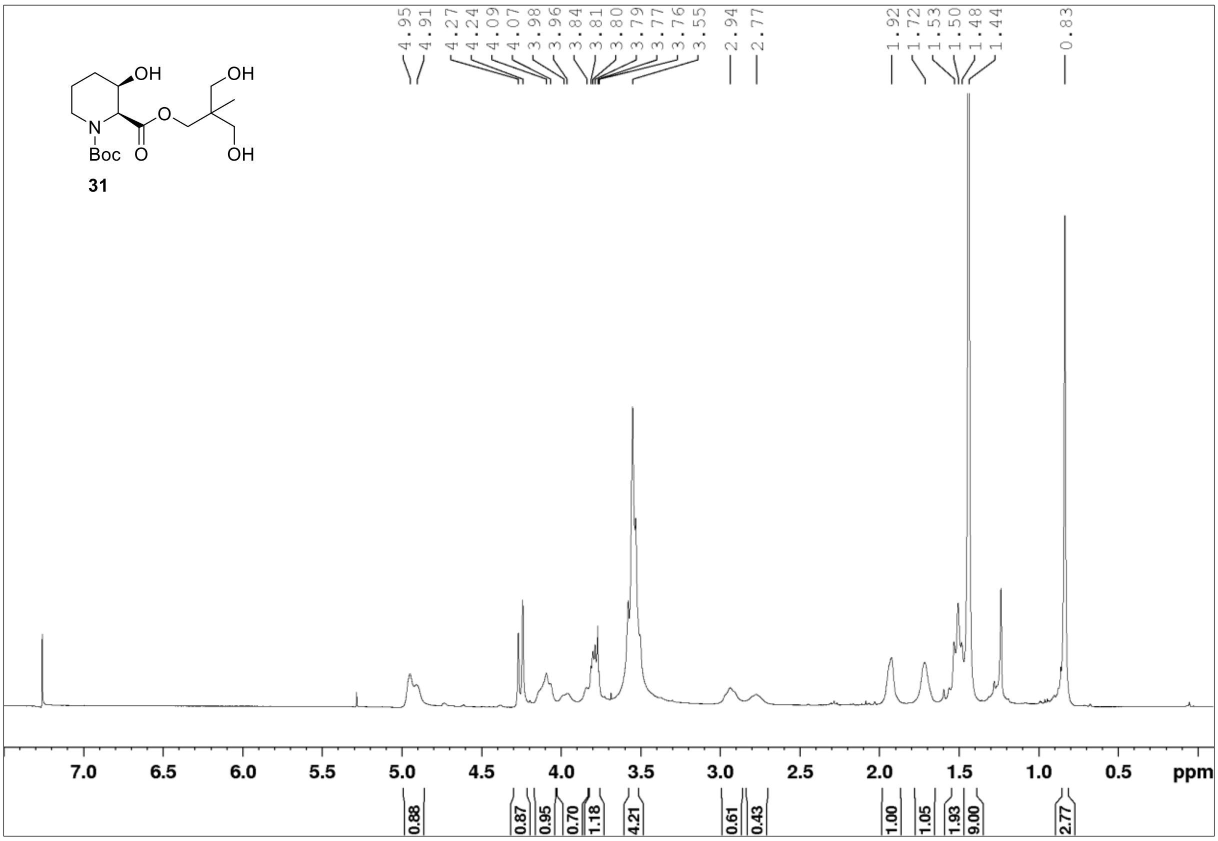

^{13}C{1H}-$CDCl_3$, 100 MHz

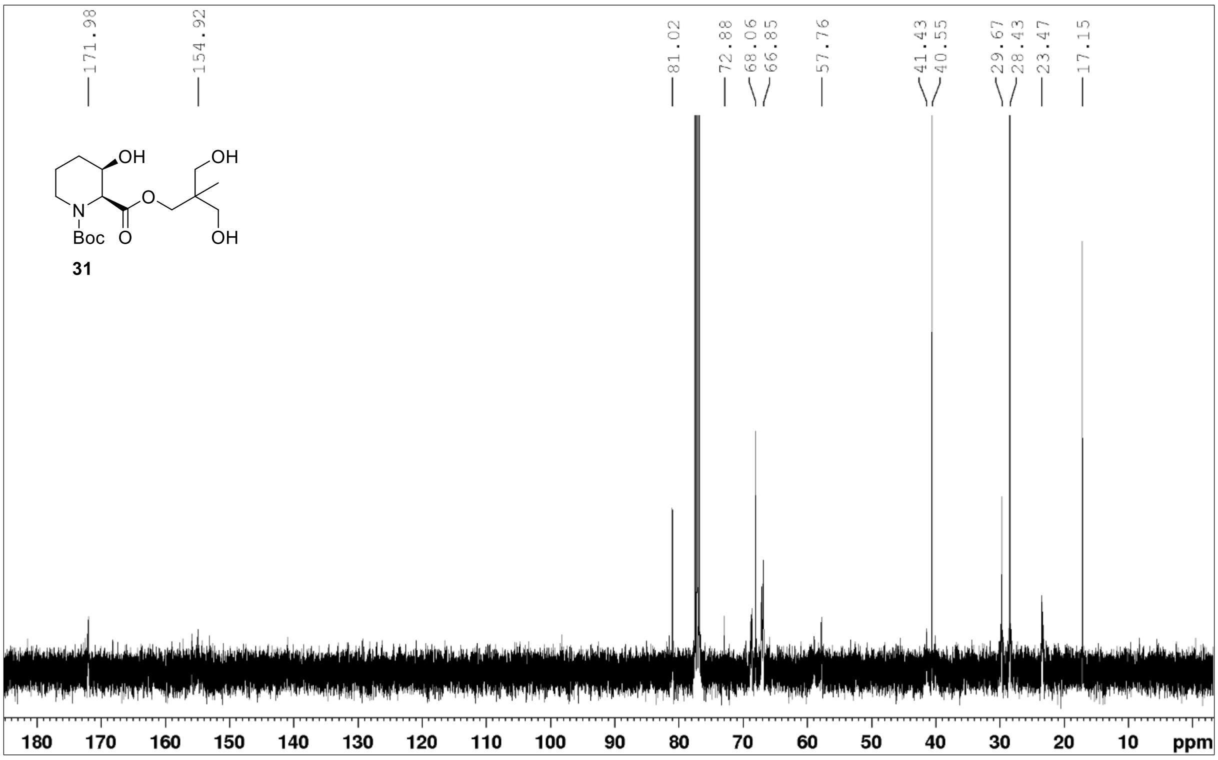

^{1}H-NMR, C_6D_6, 400 MHz

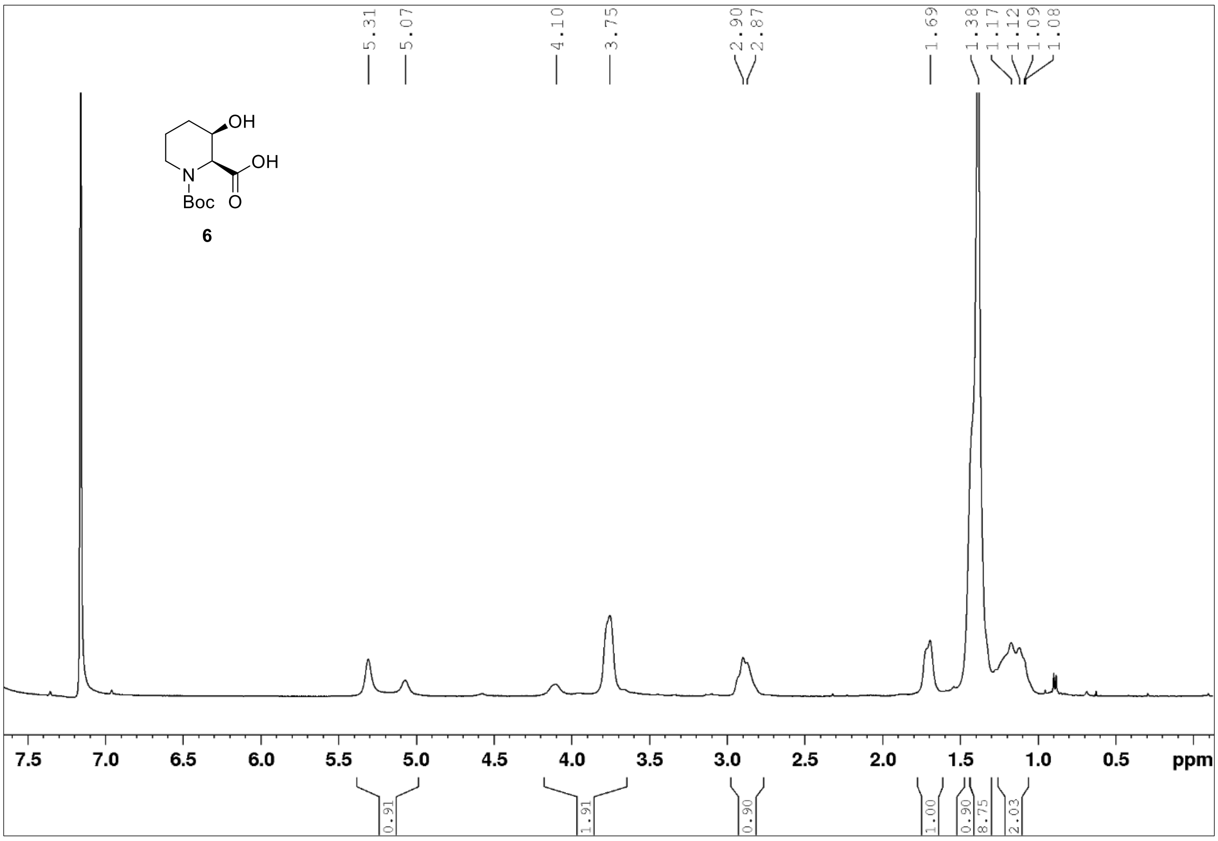

$^{13}C\{1H\}$-C_6D_6, 100 MHz

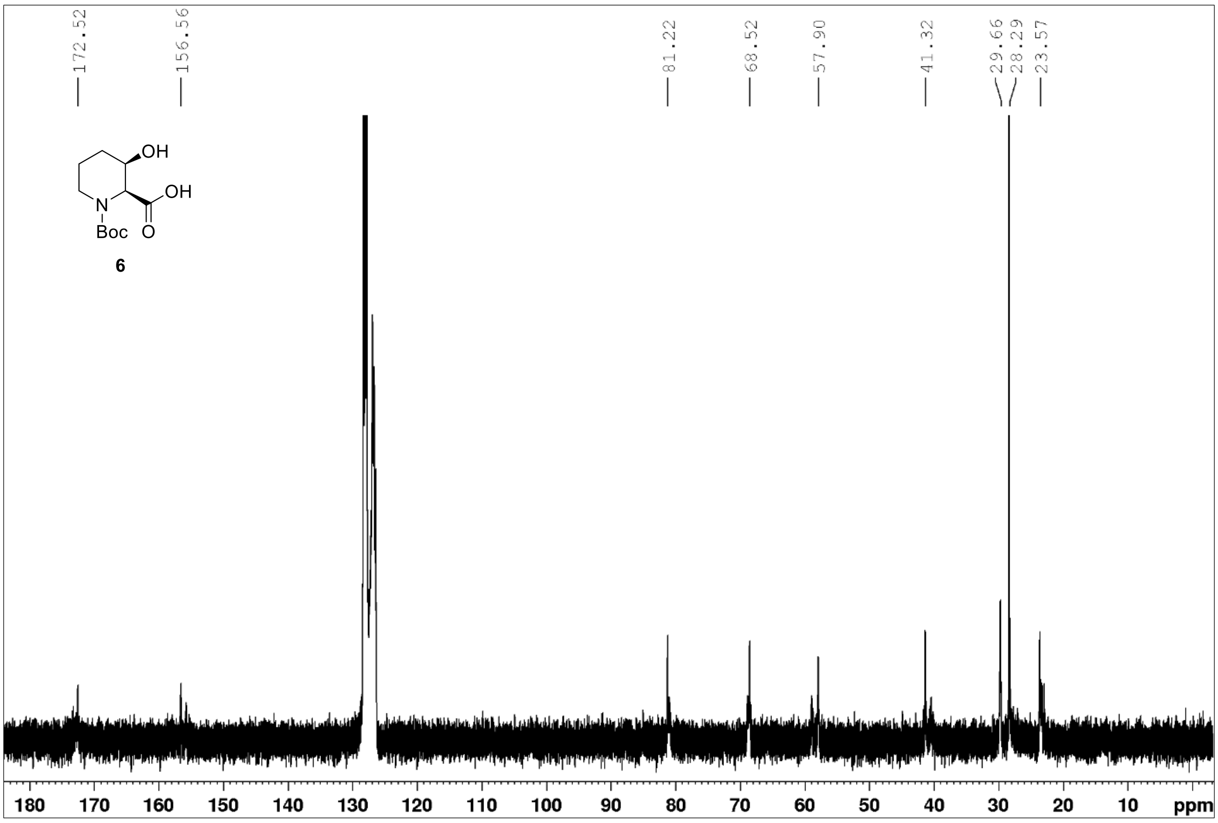

^{1}H-NMR, D_2O, 400 MHz

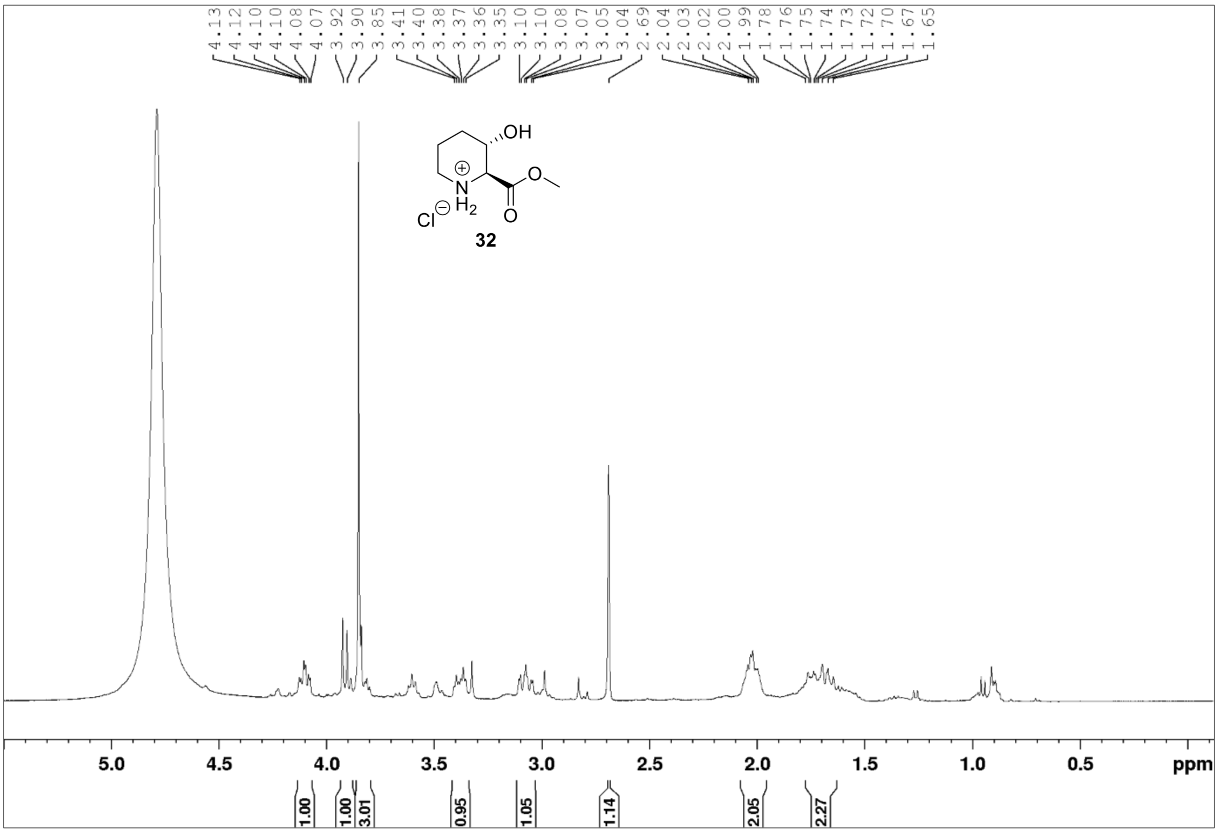

^{13}C{1H}-D_2O, 100 MHz

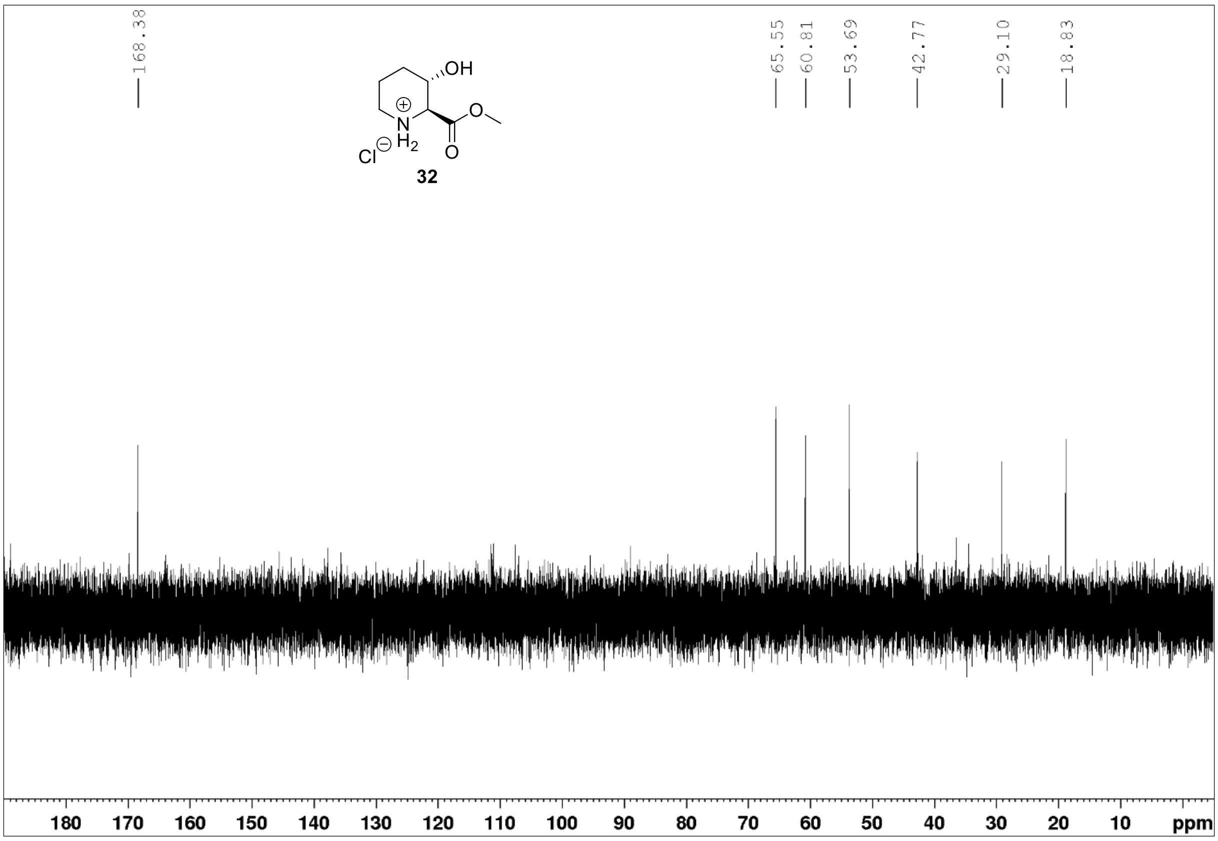

^{1}H-NMR, $CDCl_3$, 400 MHz

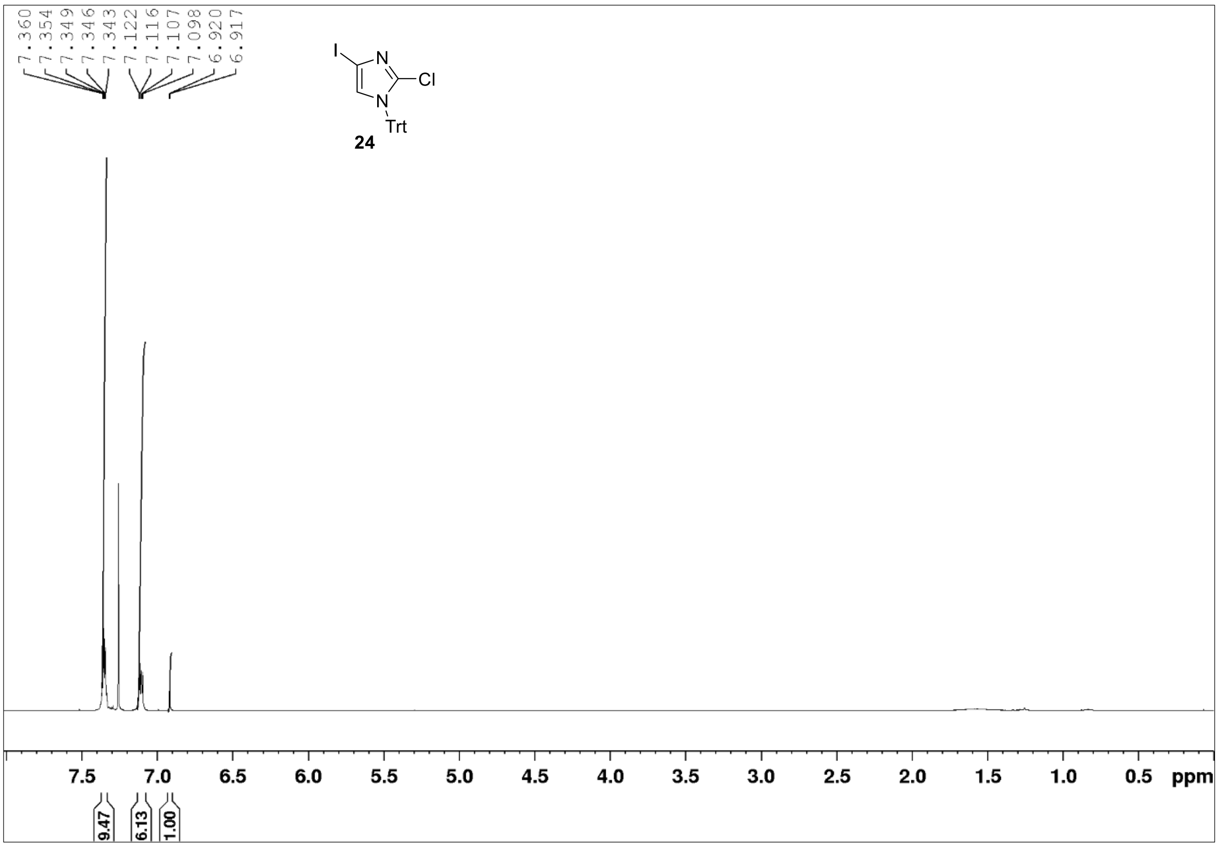

^{13}C{1H}-$CDCl_3$, 100 MHz

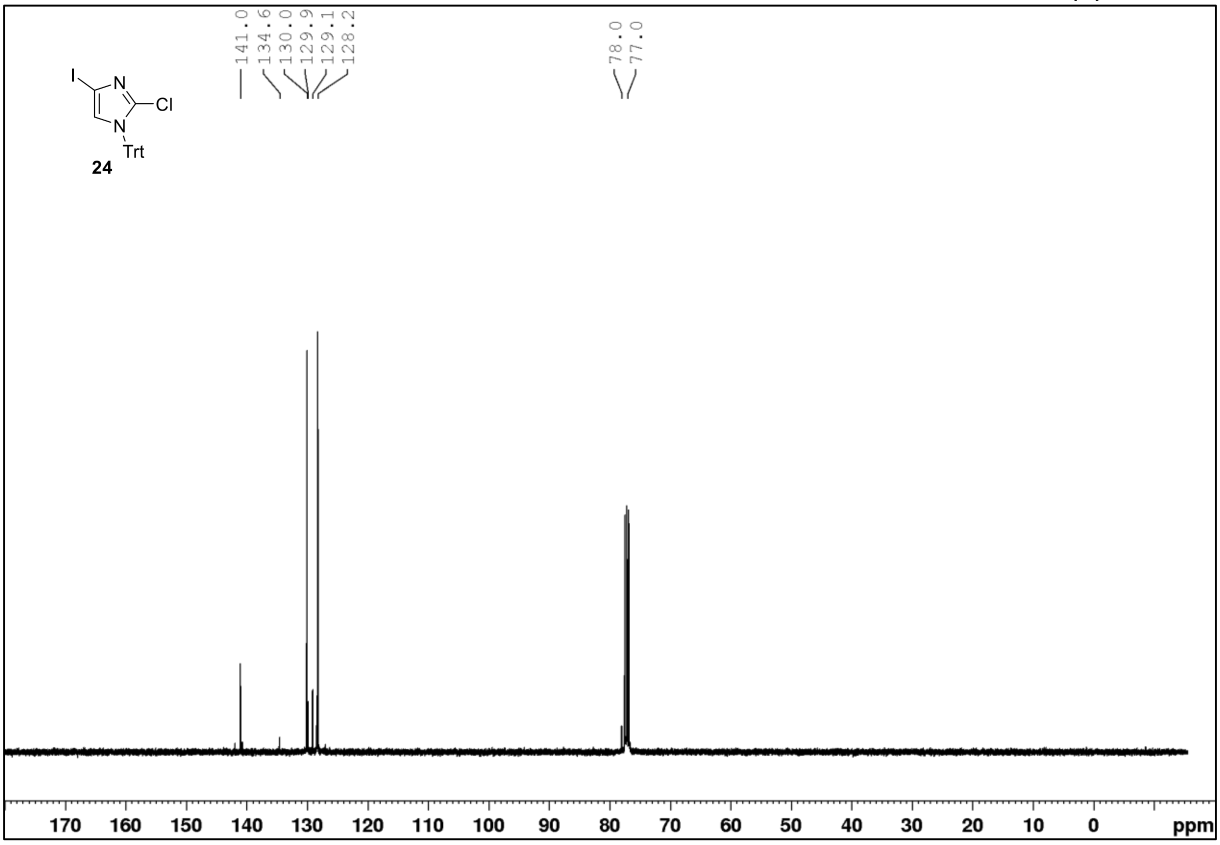

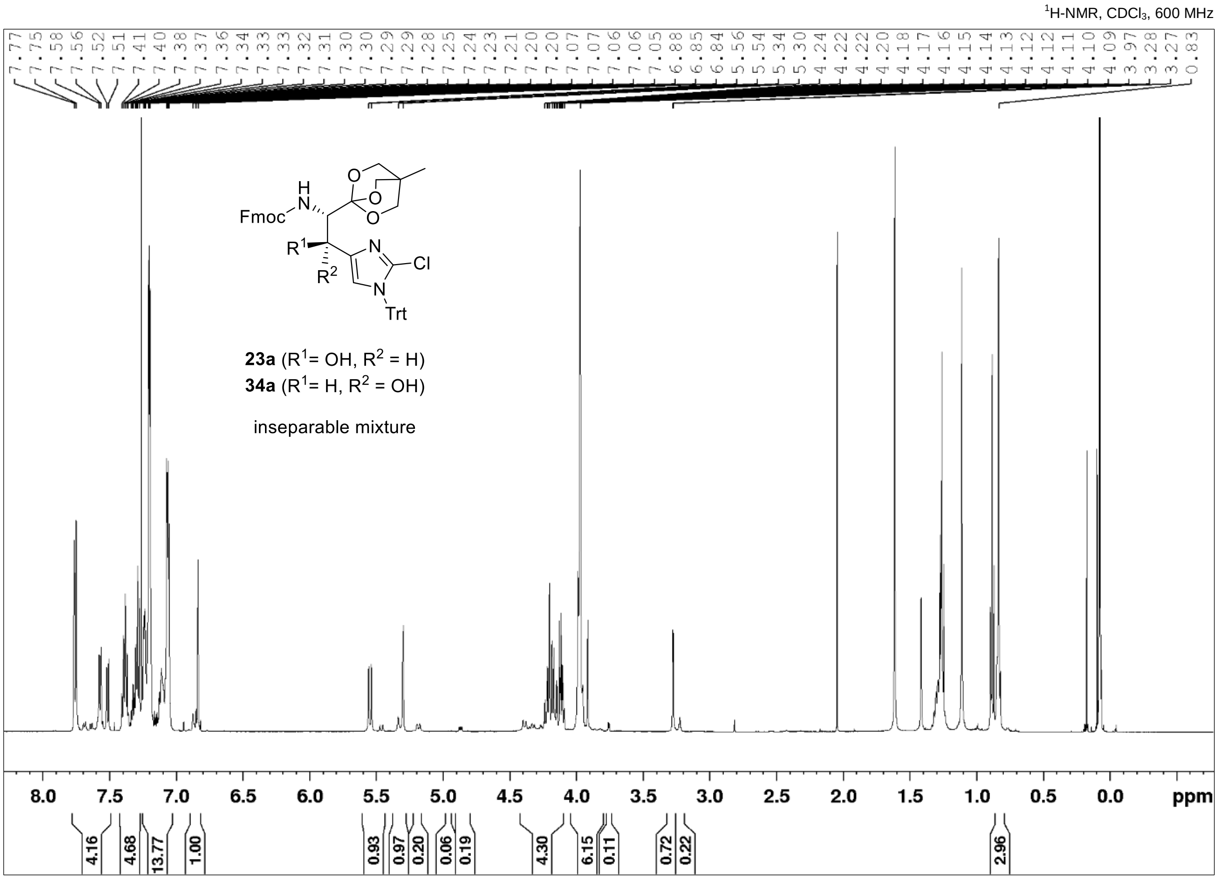

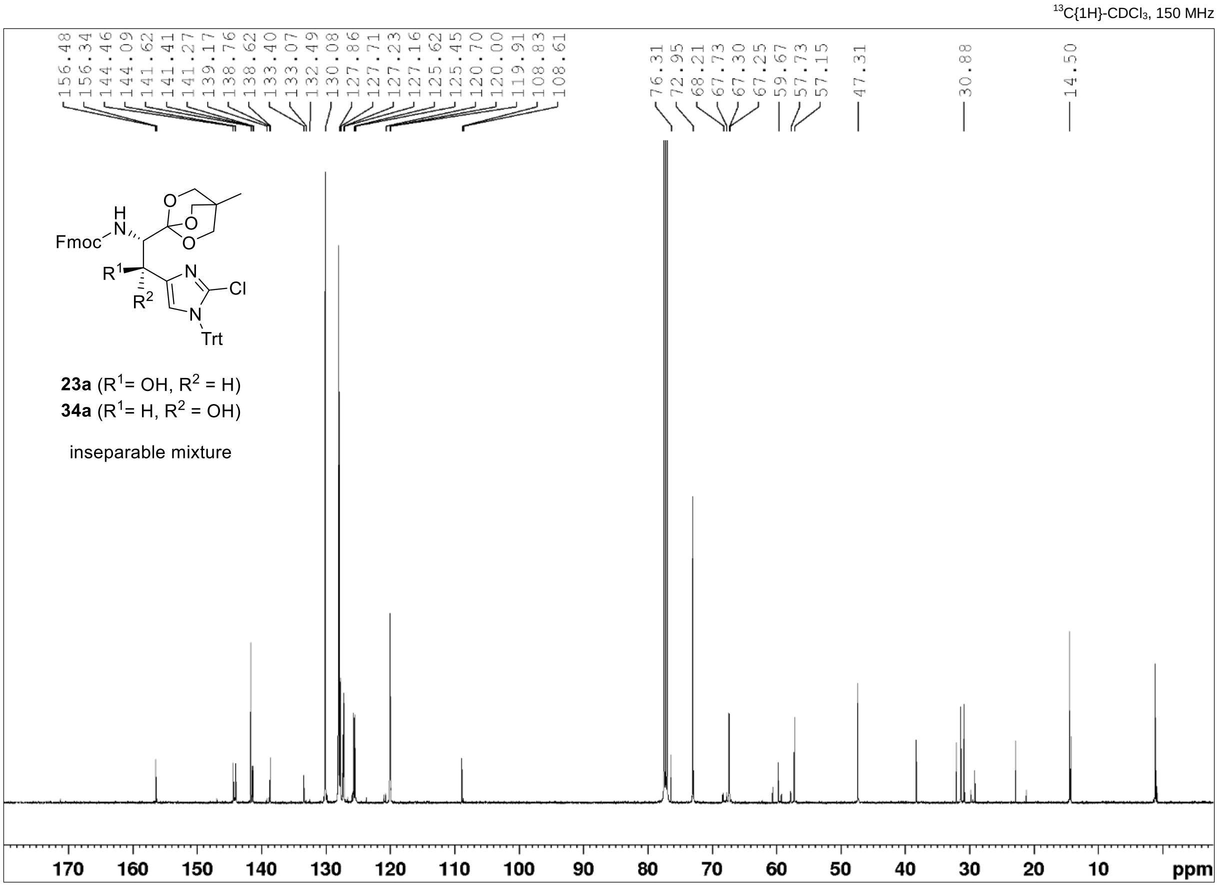

(NMR spectra at 600/150 MHz of the mixture of **23a** and **34a** for *dr* determination and acurate determination of the coupling constants – mixture contained traces of solvent and grease. Pure spectra of the mixture of **23a** and **34a** at 400/100 MHz see below)

^{1}H-NMR, $CDCl_3$, 400 MHz

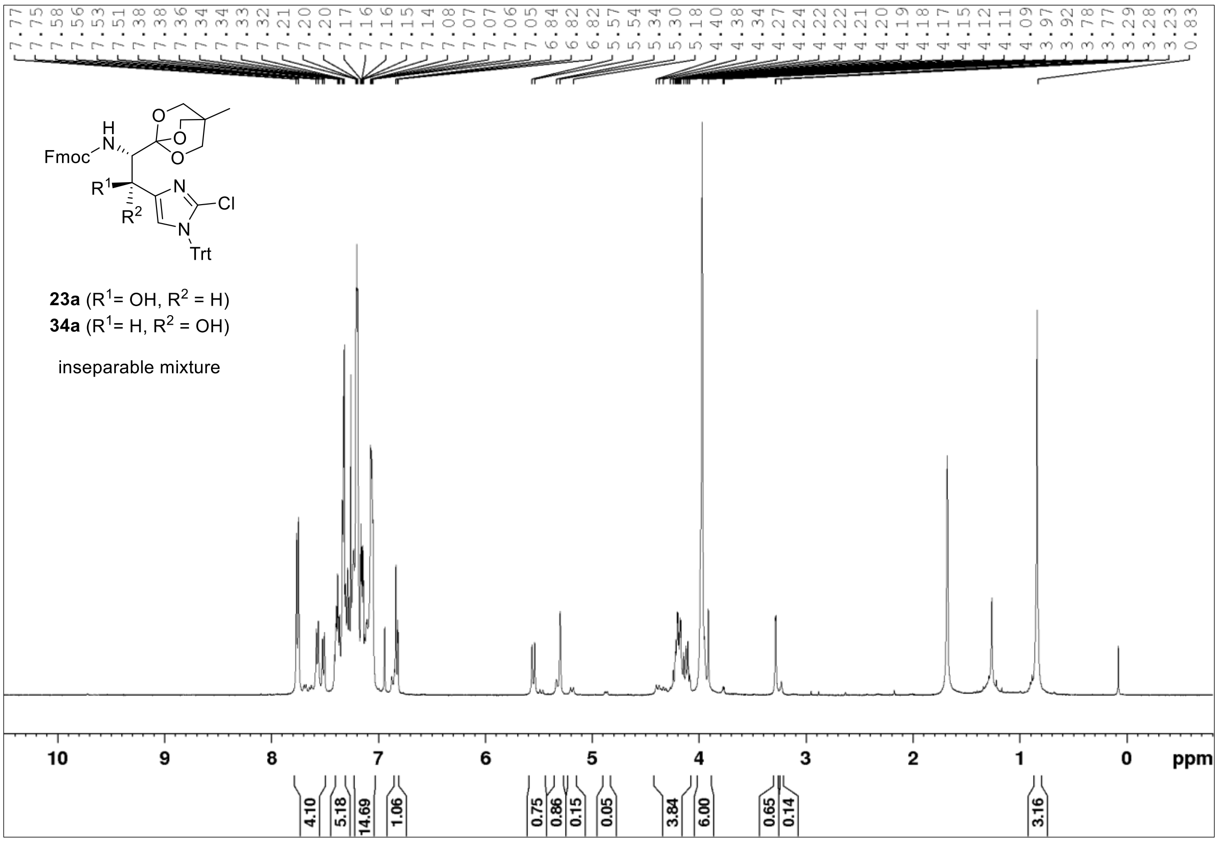

^{13}C{1H}-$CDCl_3$, 100 MHz

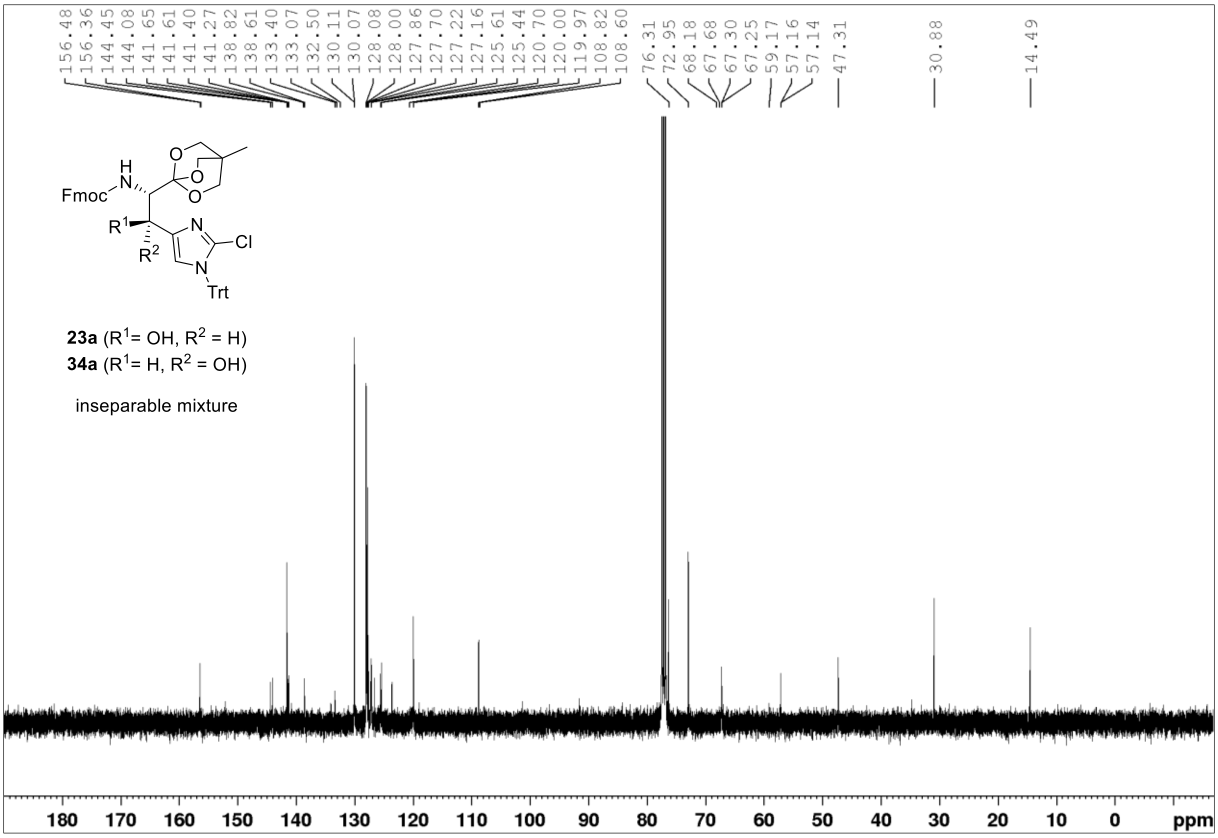

^{1}H-NMR, CDCl$_3$, 400 MHz

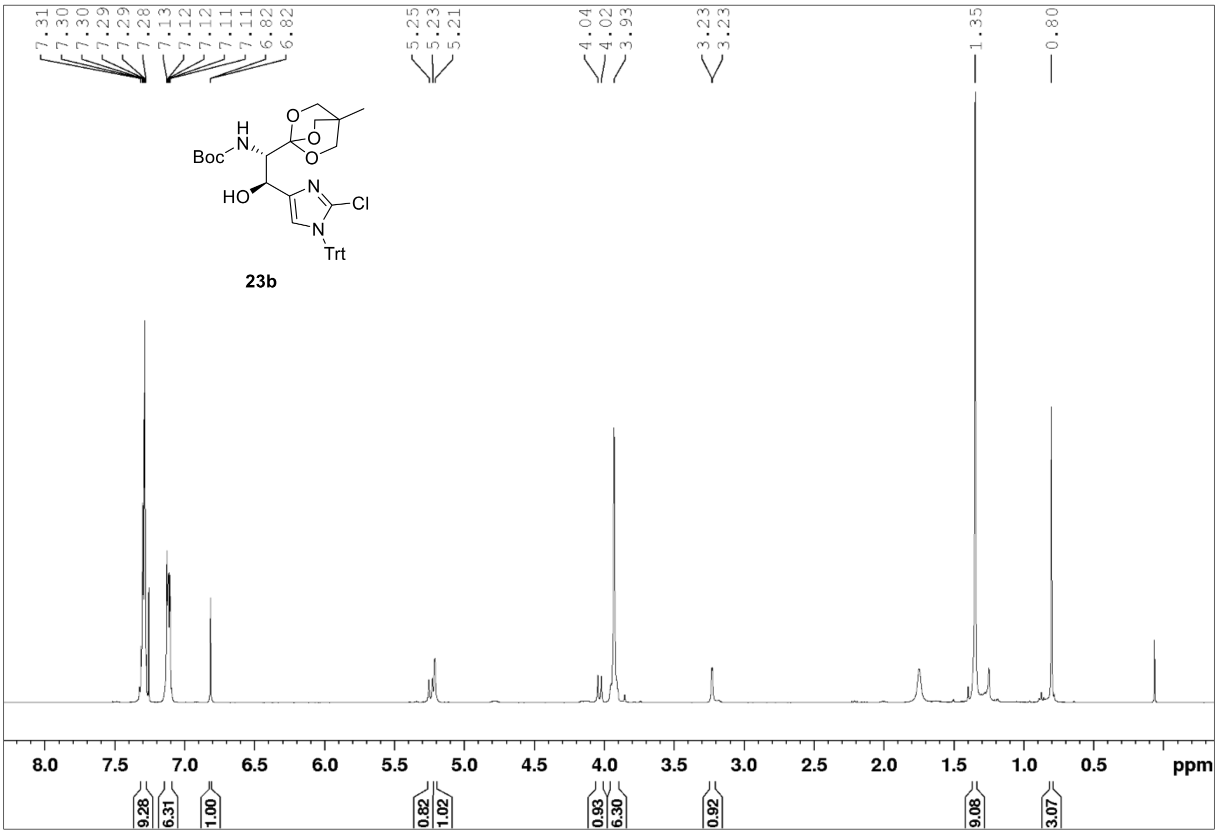

^{13}C{1H}-CDCl$_3$, 100 MHz

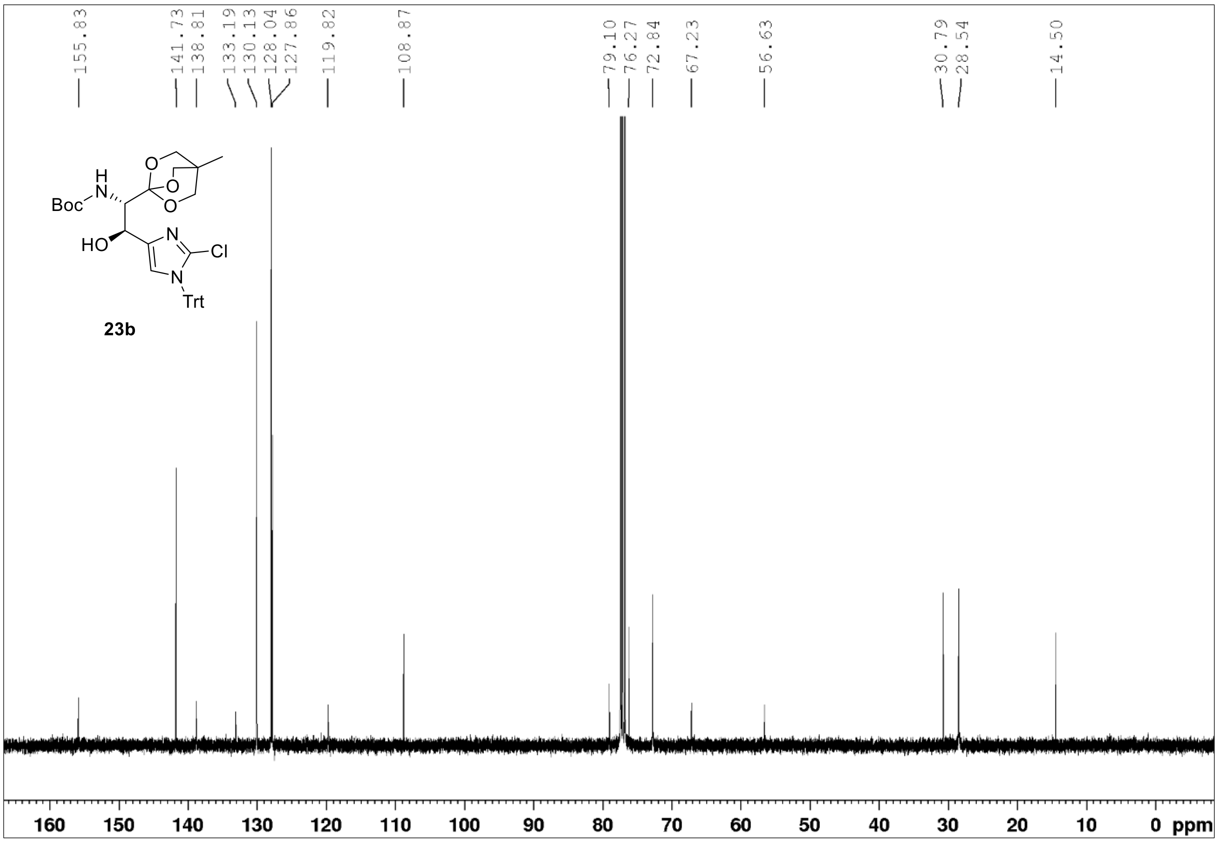

^{1}H-NMR, $CDCl_3$, 400 MHz

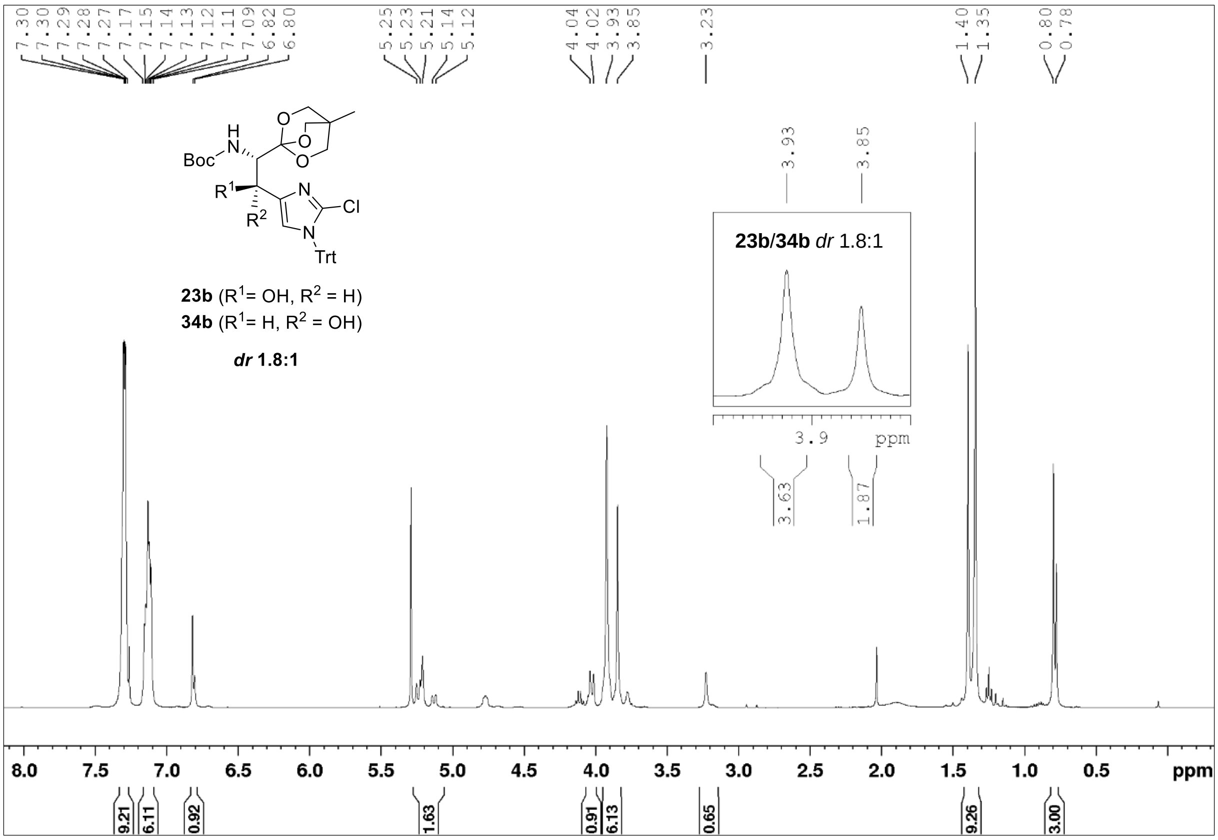

^{1}H-NMR, $CDCl_3$, 400 MHz

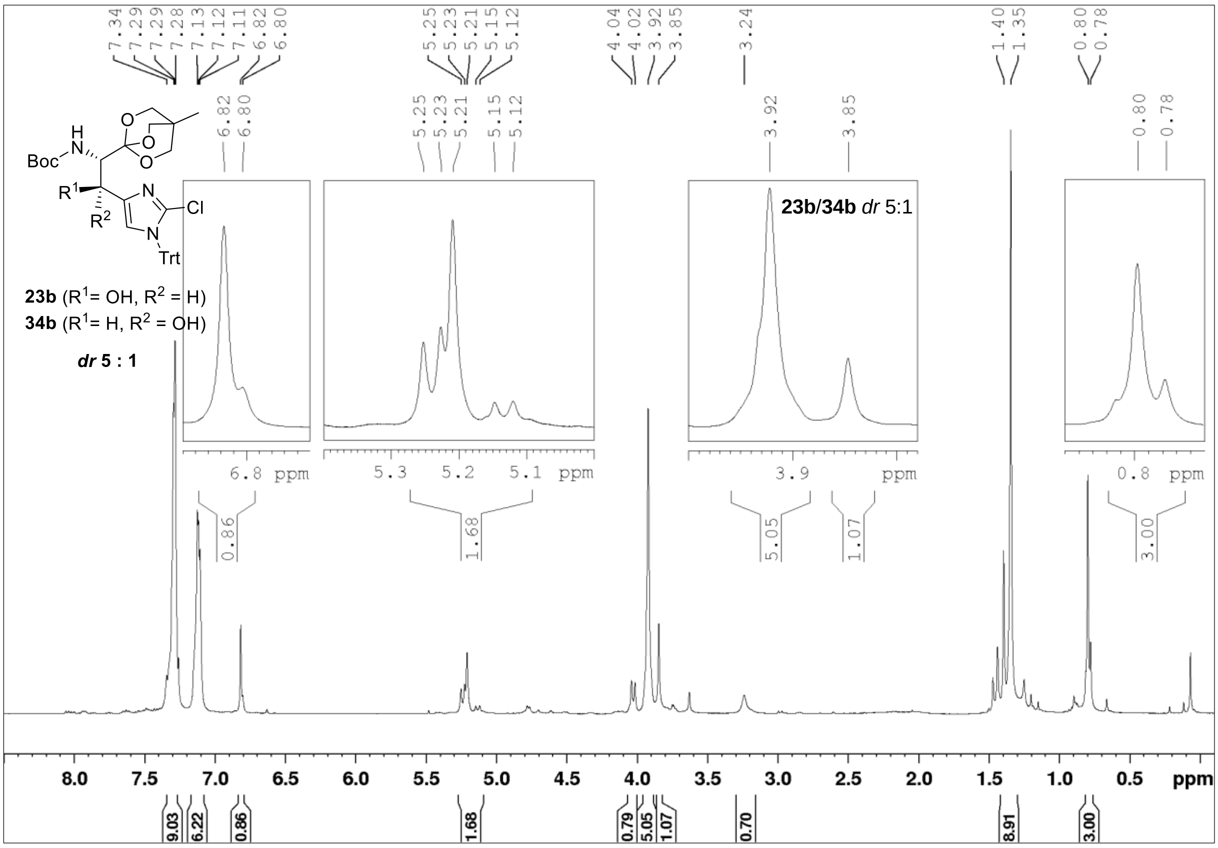

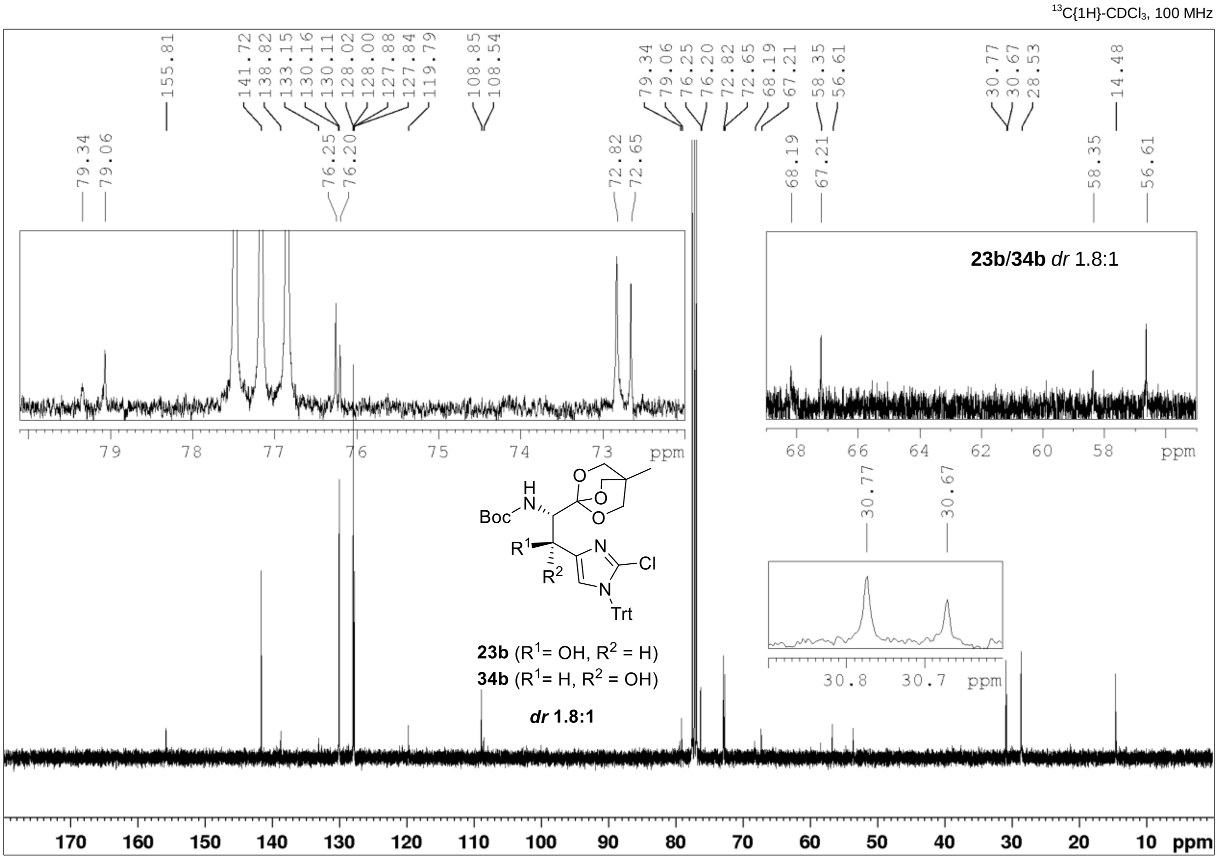

13C{1H}-CDCl3, 100 MHz
155.81
141.72
138.82
133.15
130.16
130.11
128.02
128.00
127.88
127.84
119.79
108.85
108.54
79.34
79.06
76.25
76.20
72.82
72.65
68.19
67.21
58.35
56.61
30.77
30.67
28.53
14.48
23b/34b dr 1.8:1
Boc
Cl
Trt
23b (R1= OH, R2 = H)
34b (R1= H, R2 = OH)
dr 1.8:1
ppm

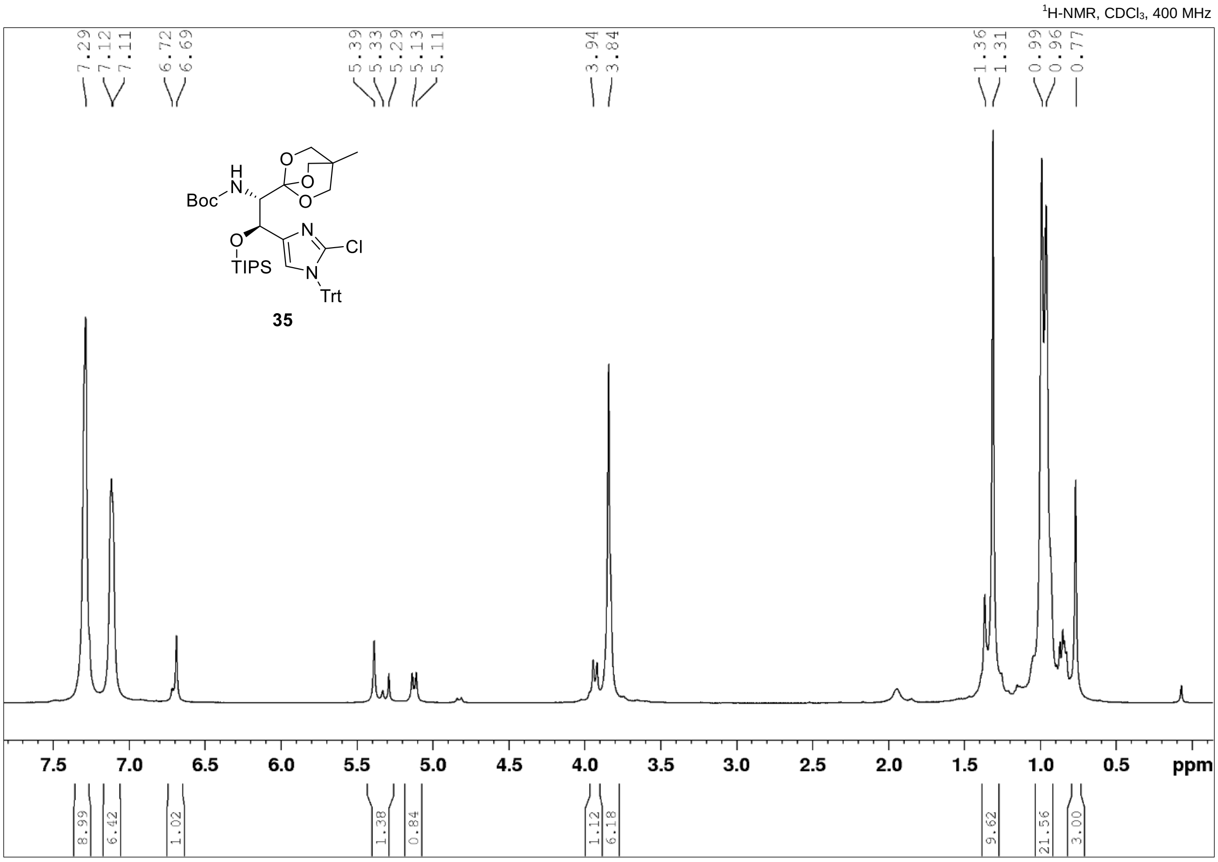
1H-NMR, CDCl3, 400 MHz
7.29
7.12
7.11
6.72
6.69
5.39
5.33
5.29
5.13
5.11
3.94
3.84
1.36
1.31
0.99
0.96
0.77
Boc
N
H
O
O
O
N
Cl
N
Trt
O
TIPS
35
7.5
7.0
6.5
6.0
5.5
5.0
4.5
4.0
3.5
3.0
2.5
2.0
1.5
1.0
0.5
ppm
8.99
6.42
1.02
1.38
0.84
1.12
6.18
9.62
21.56
3.00

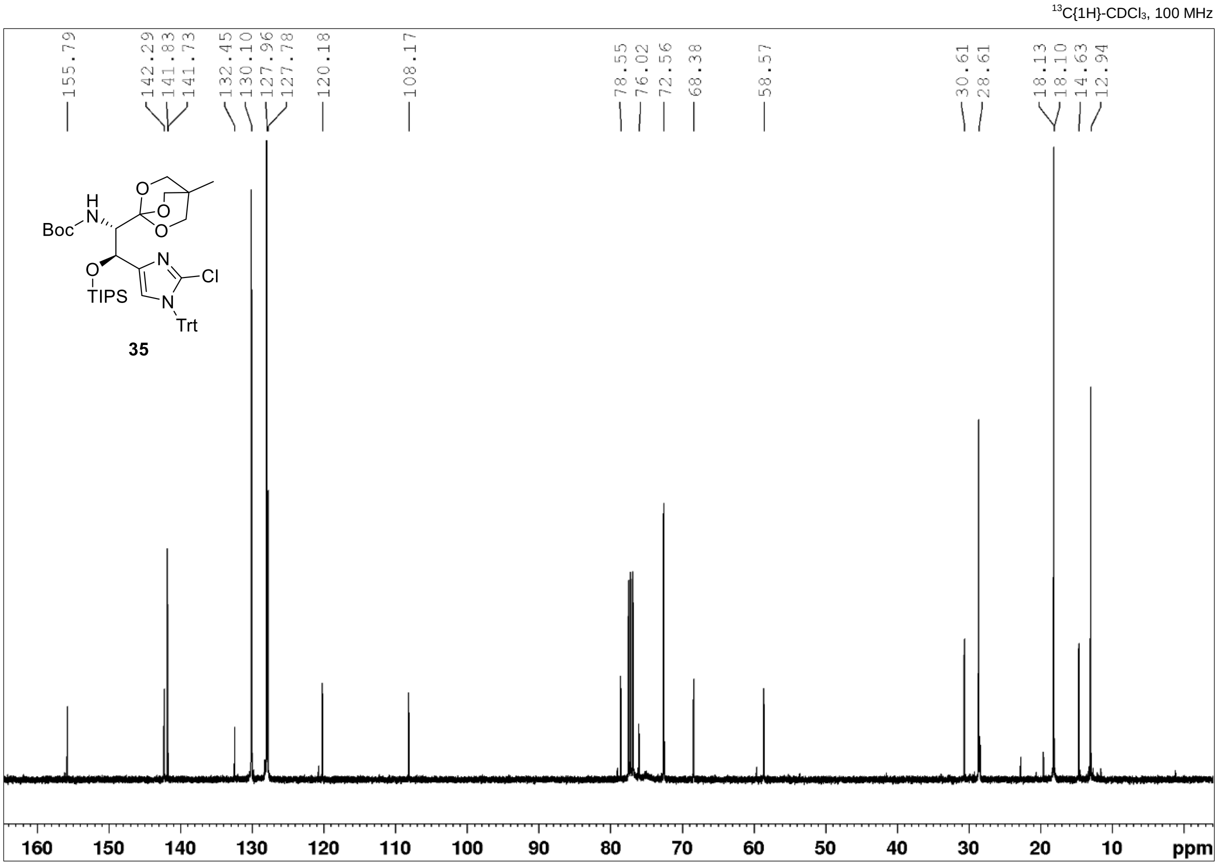
13C{1H}-CDCl3, 100 MHz
155.79
142.29
141.83
141.73
132.45
130.10
127.96
127.78
120.18
108.17
78.55
76.02
72.56
68.38
58.57
30.61
28.61
18.13
18.10
14.63
12.94
Boc
N
H
O
O
O
N
Cl
N
Trt
O
TIPS
35
160
150
140
130
120
110
100
90
80
70
60
50
40
30
20
10
ppm

^{1}H-NMR, CDCl$_3$, 600 MHz

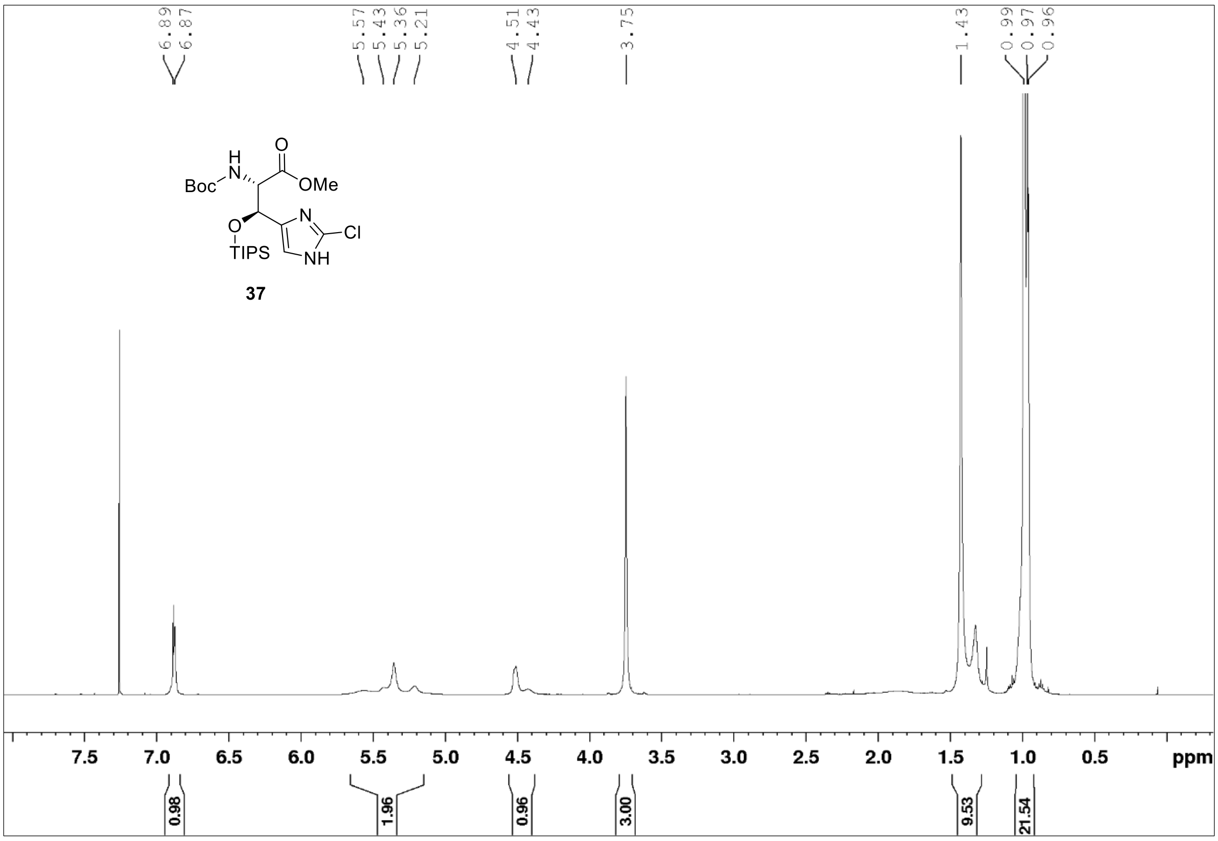

^{13}C{1H}-CDCl$_3$, 150 MHz

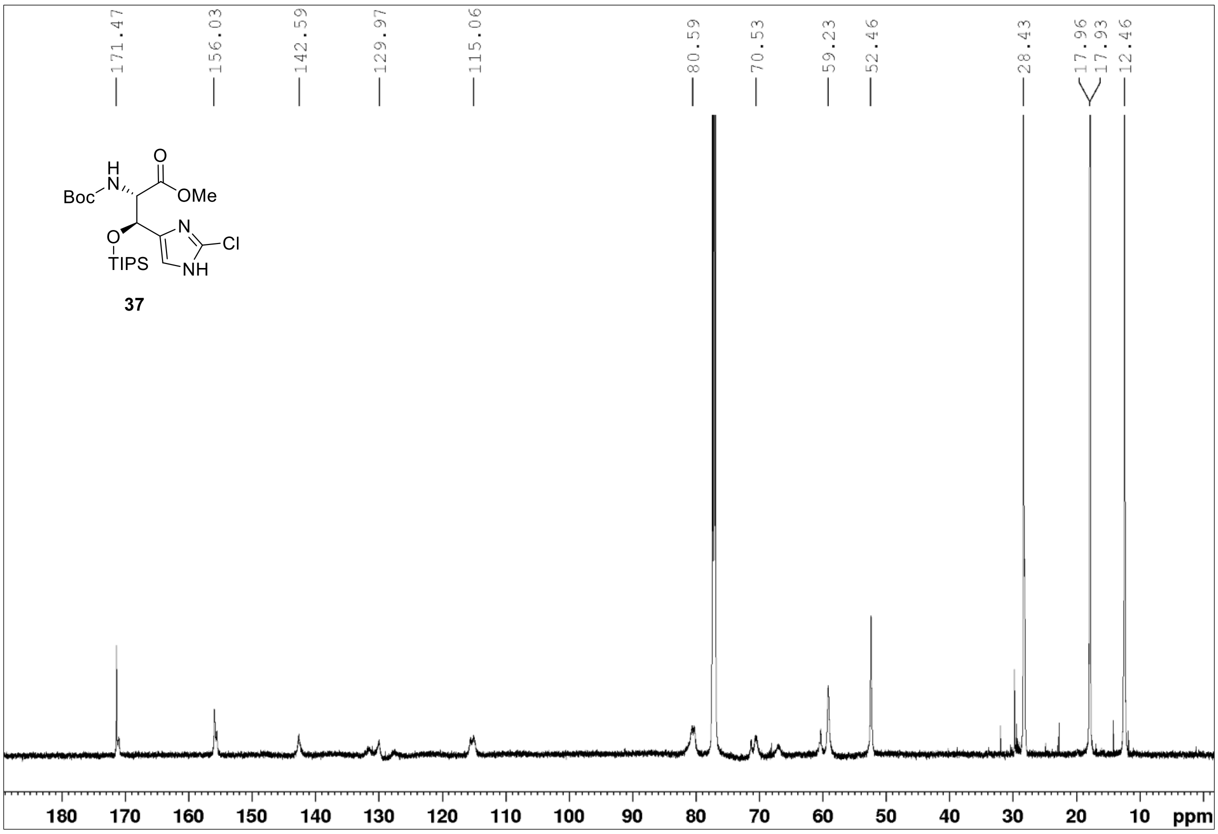

^{1}H-NMR, $CDCl_3$, 400 MHz

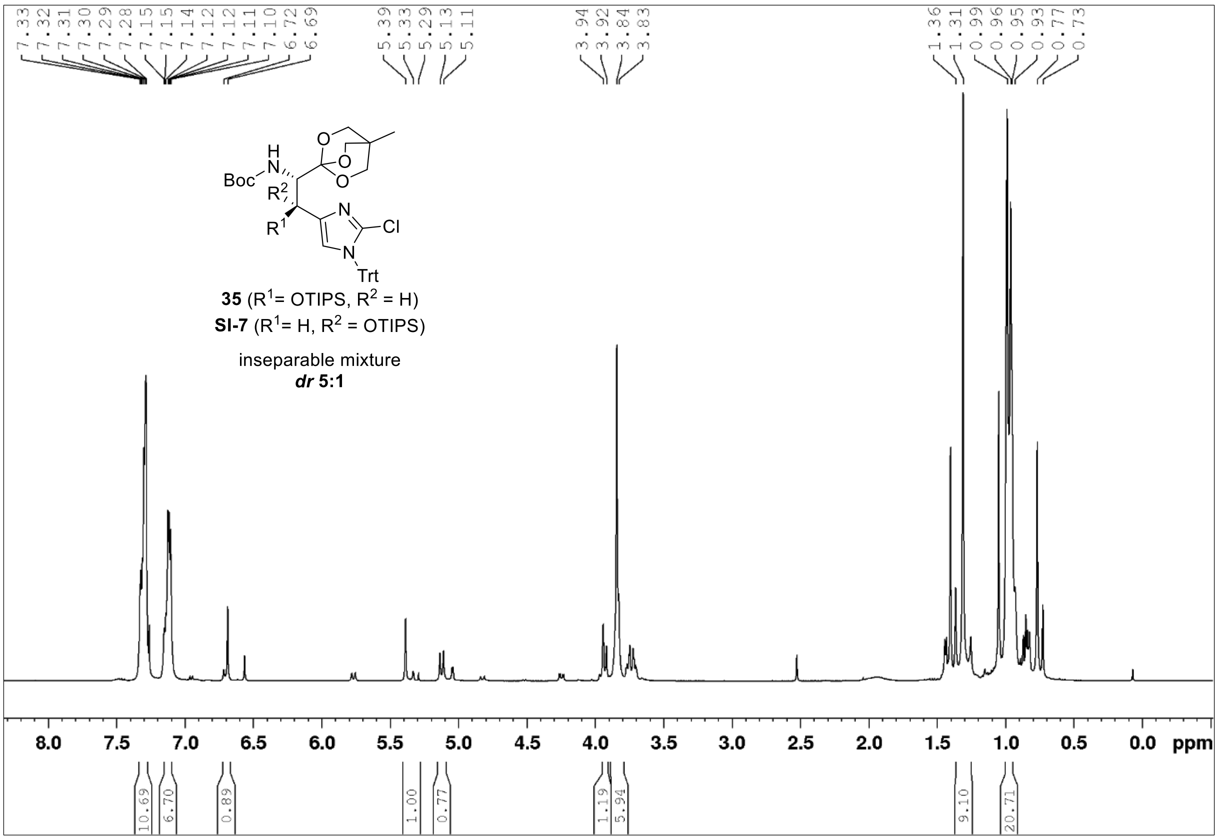

^{13}C{1H}-$CDCl_3$, 100 MHz

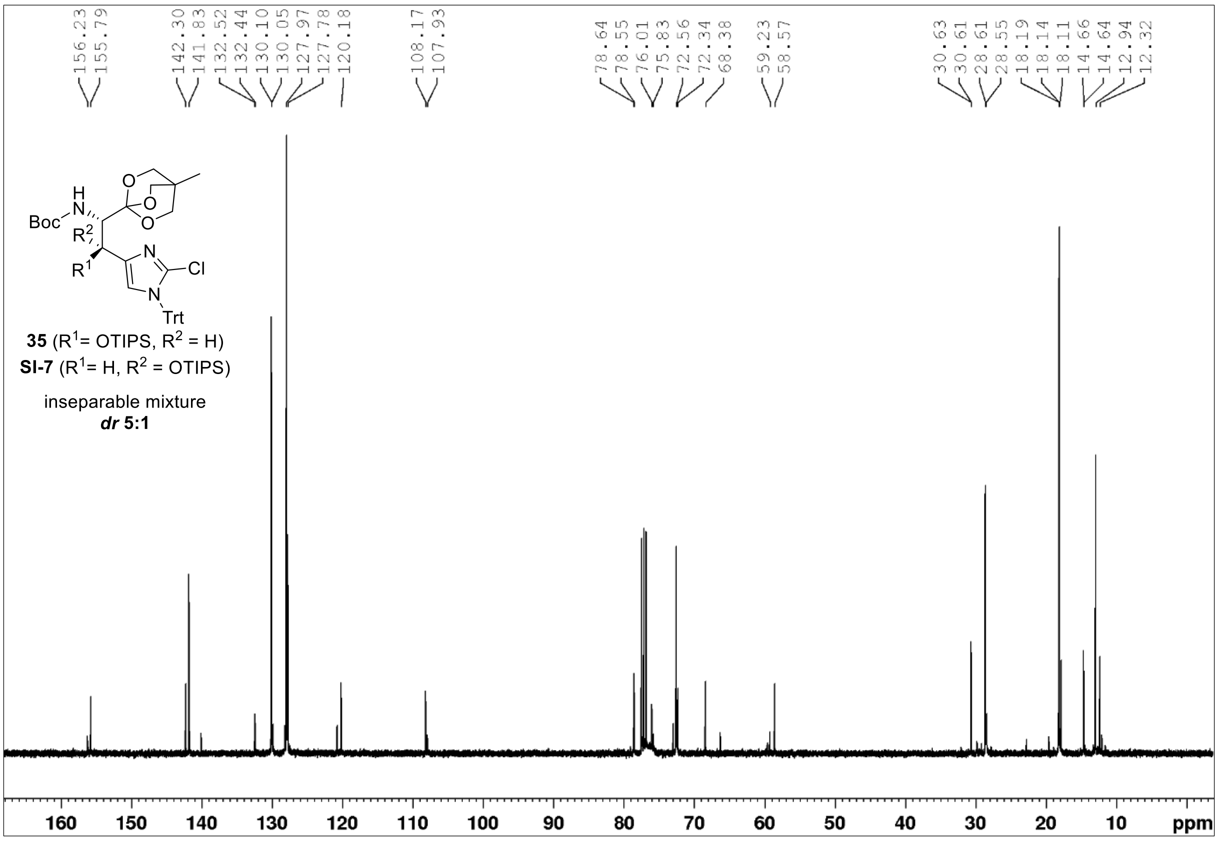

^{1}H-NMR, $CDCl_3$, 600 MHz

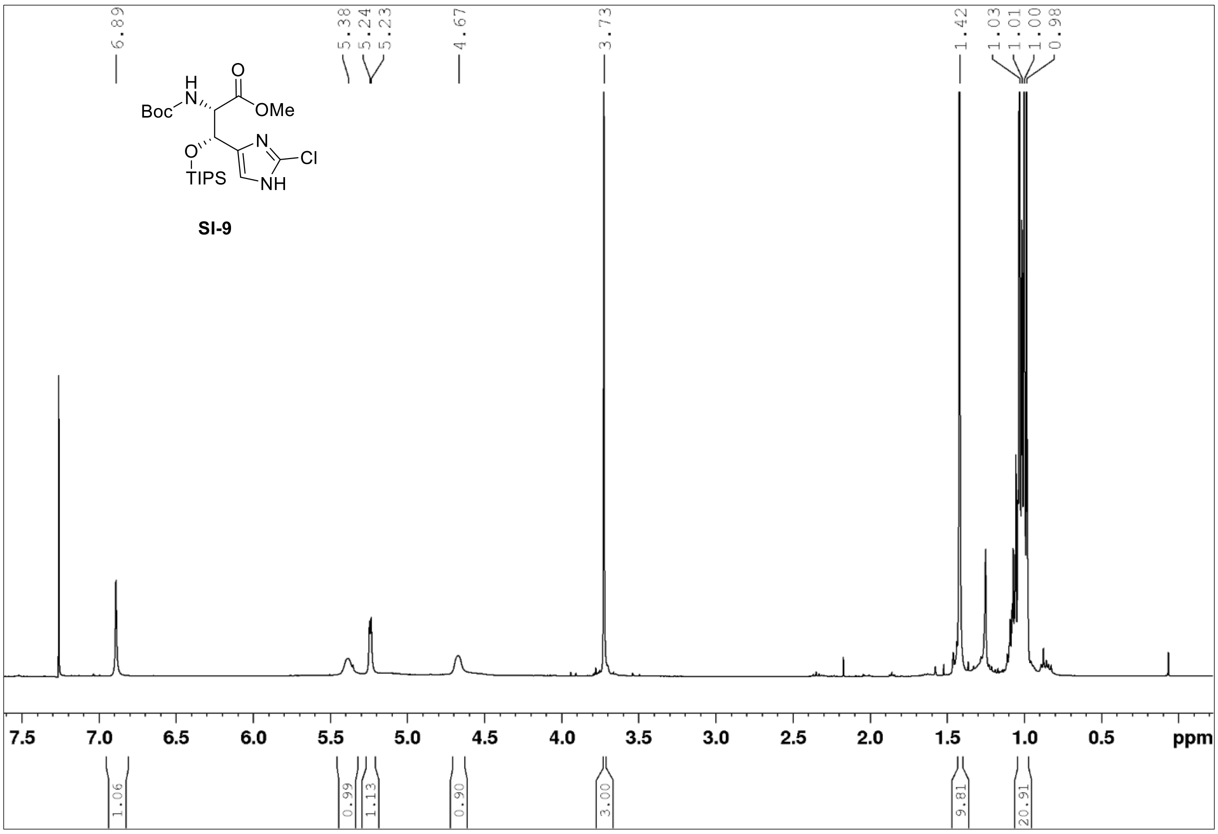

^{13}C{1H}-$CDCl_3$, 150 MHz

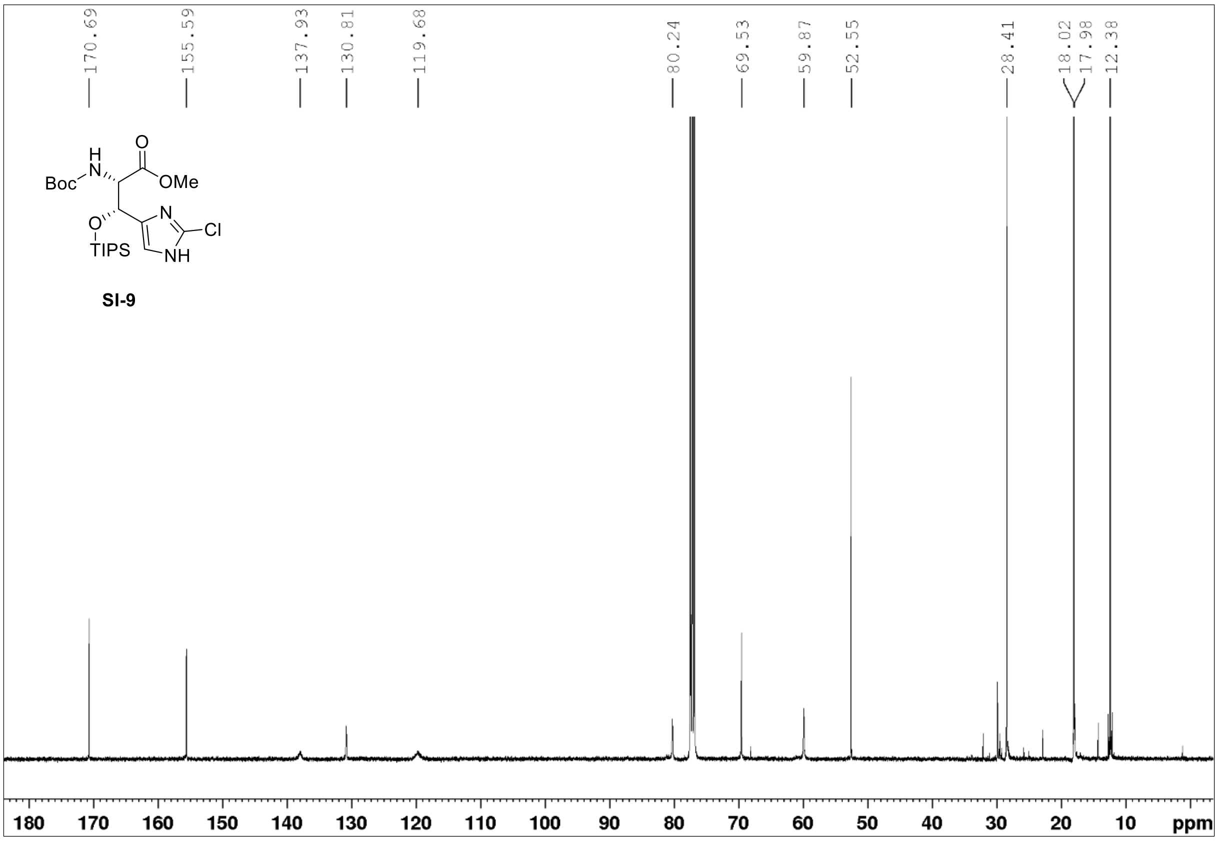

^{1}H-NMR, CDCl$_3$, 400 MHz

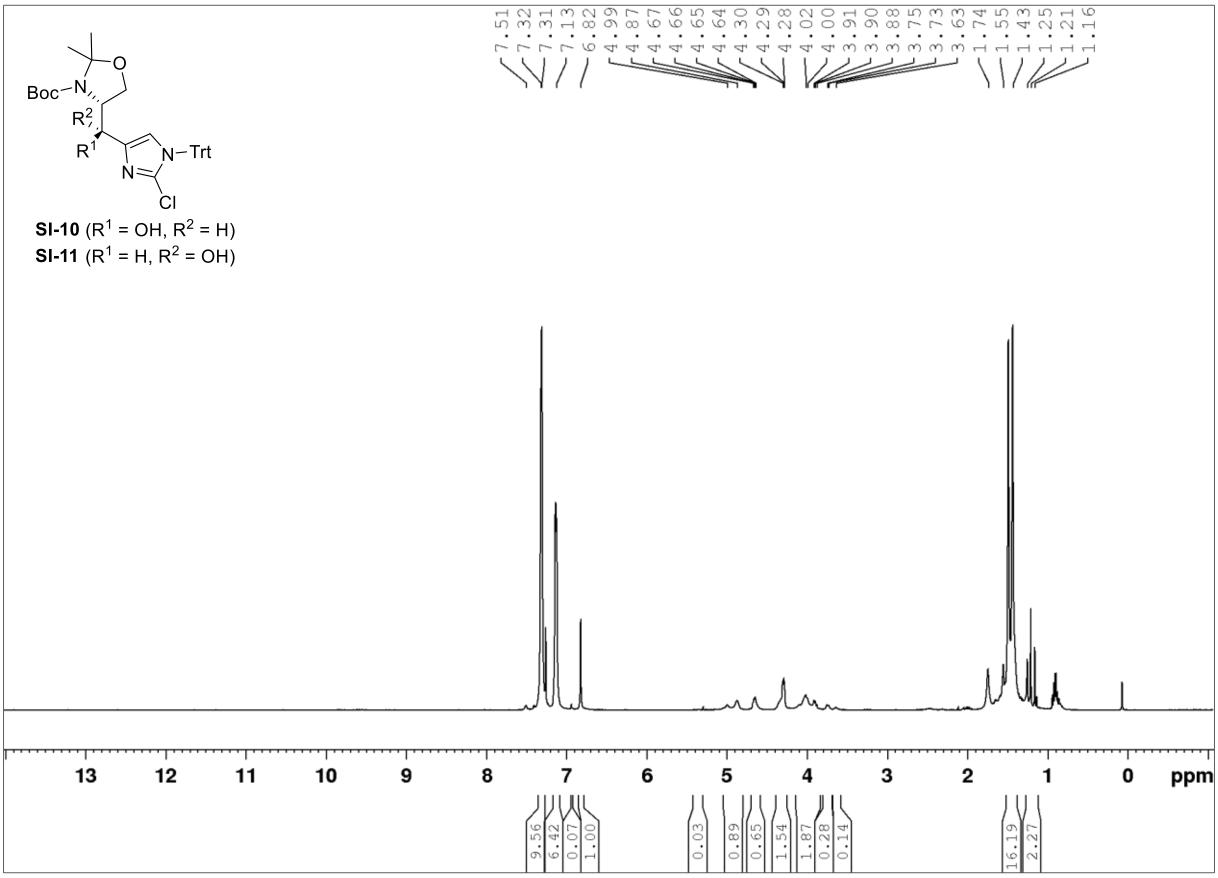

^{13}C{1H}-CDCl$_3$, 100 MHz

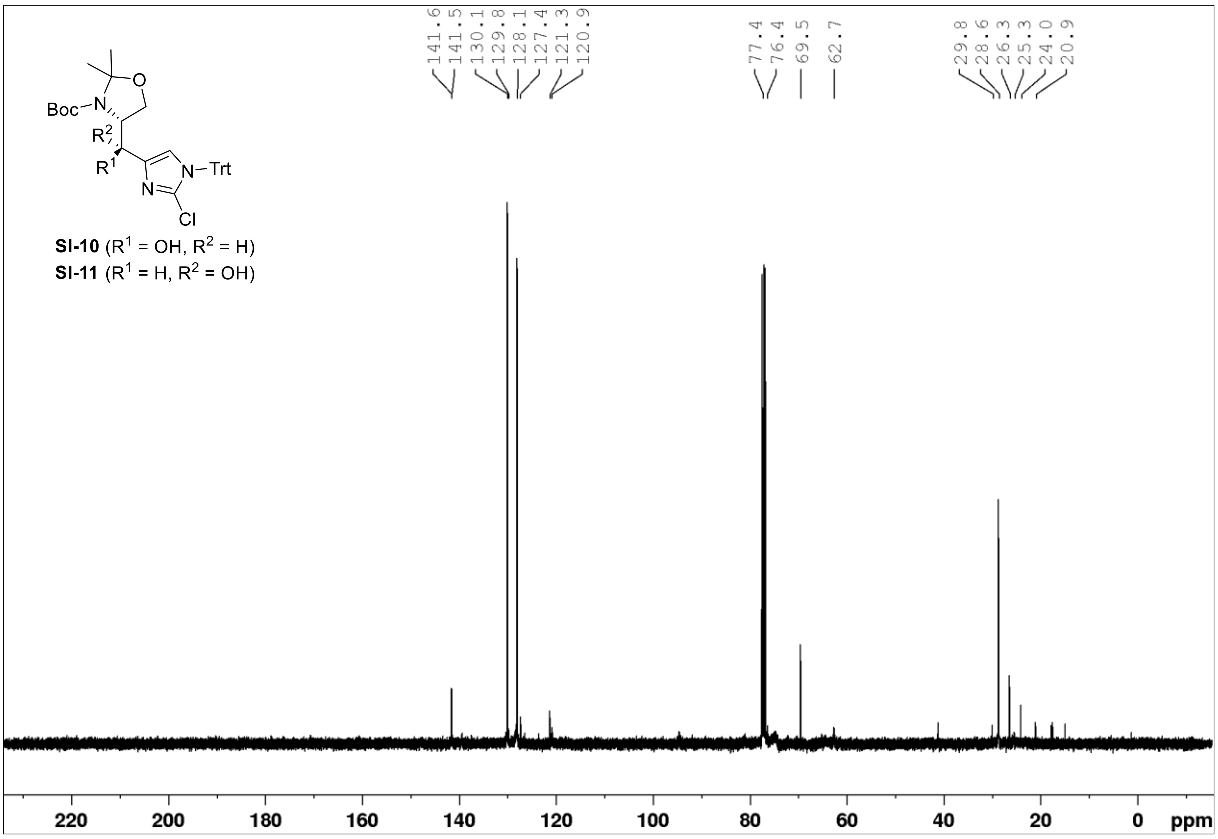

^{1}H-NMR, $CDCl_3$, 400 MHz

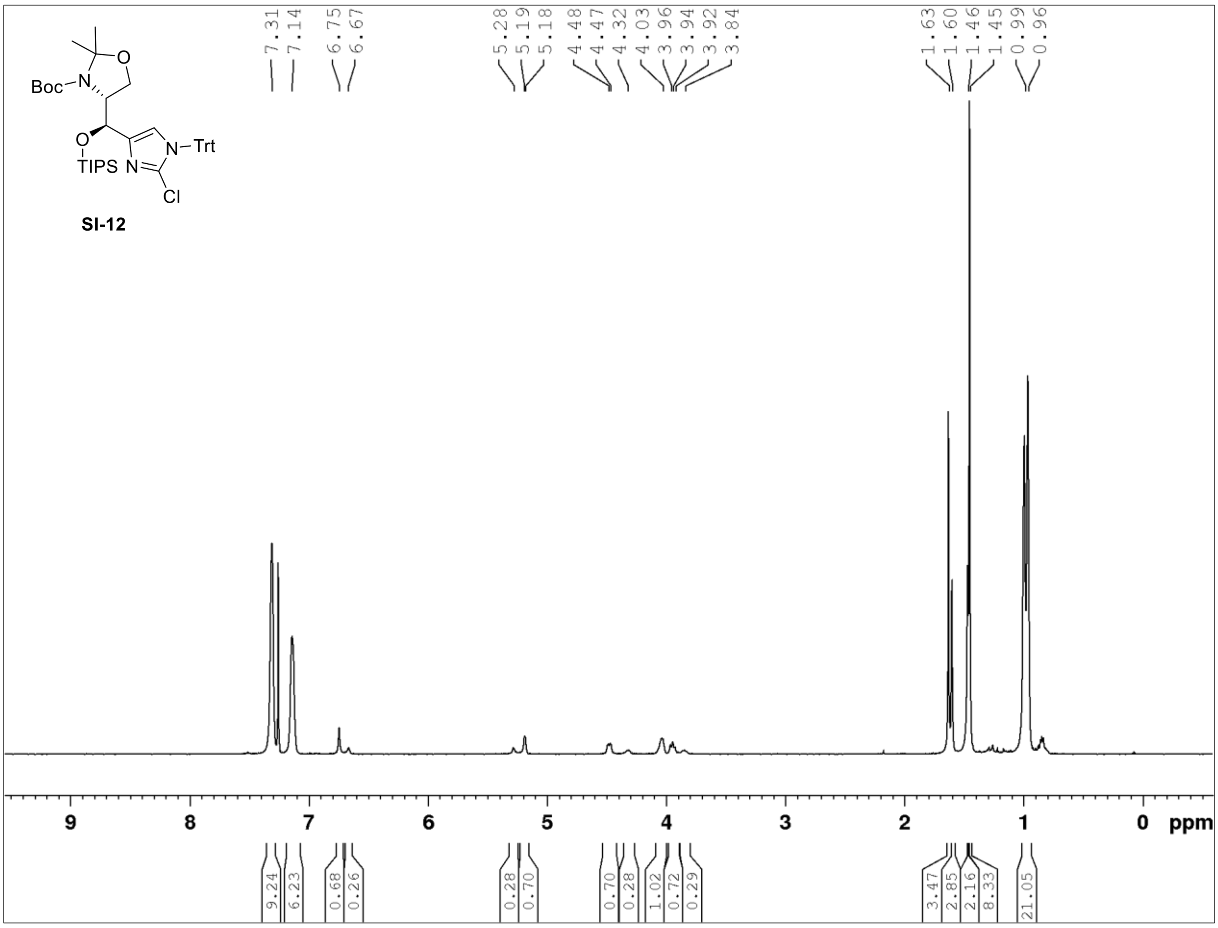

^{13}C{1H}-$CDCl_3$, 100 MHz

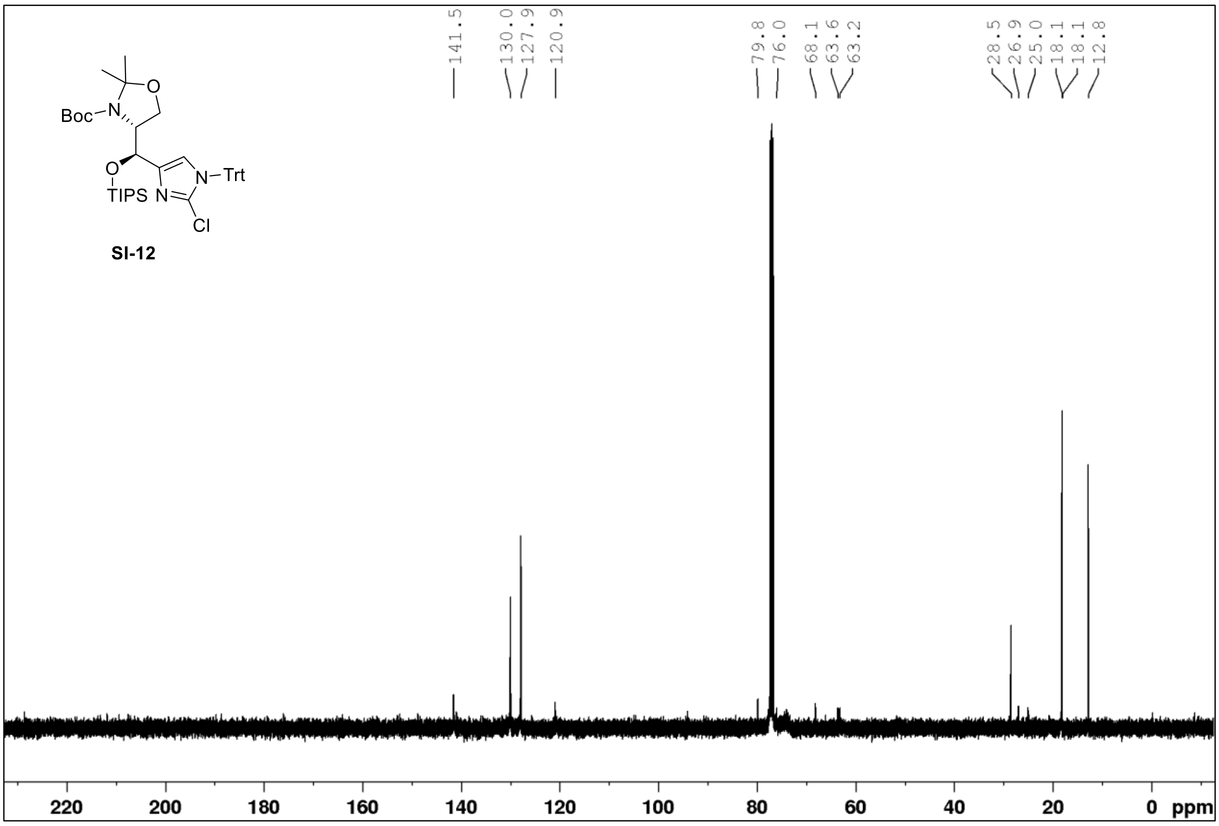

^{1}H-NMR, $CDCl_3$, 400 MHz

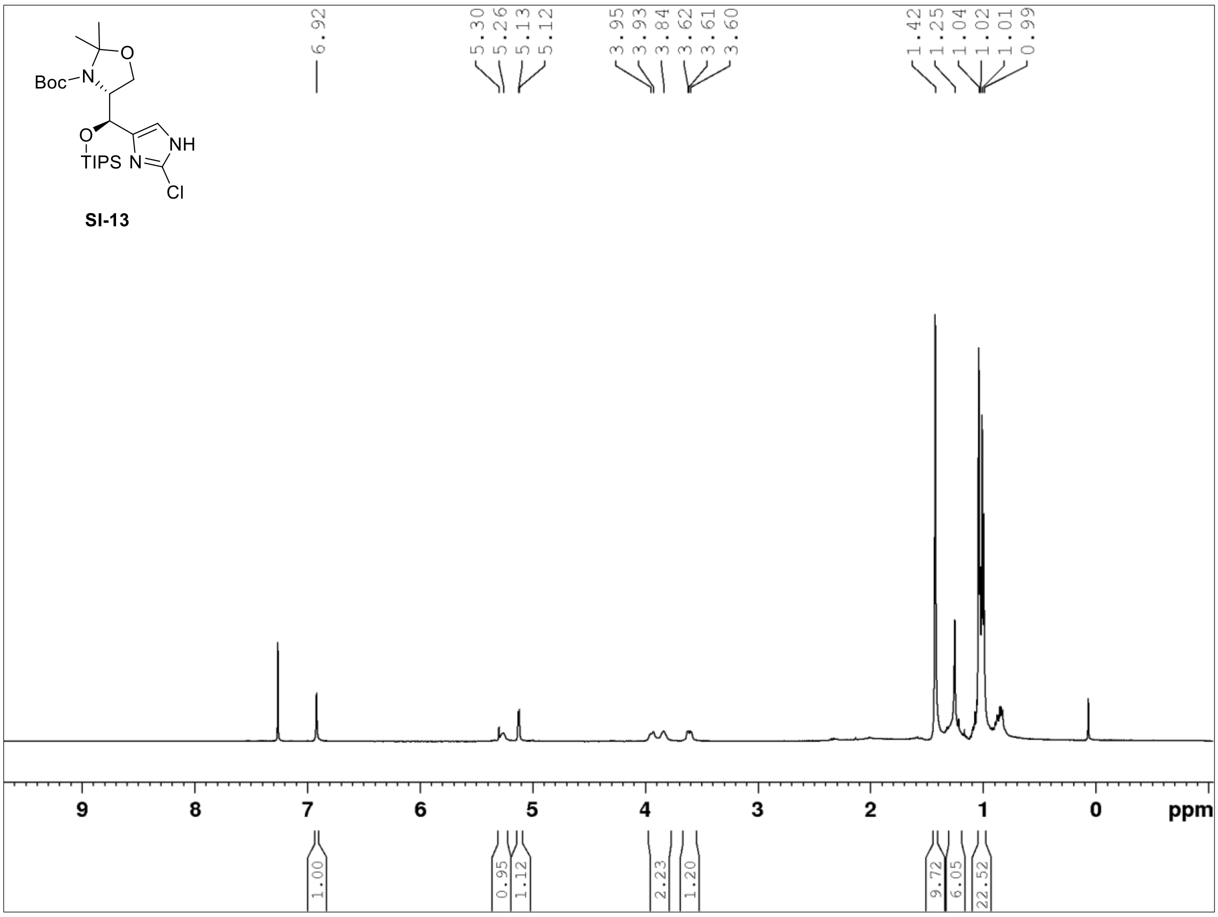

^{13}C{1H}-$CDCl_3$, 100 MHz

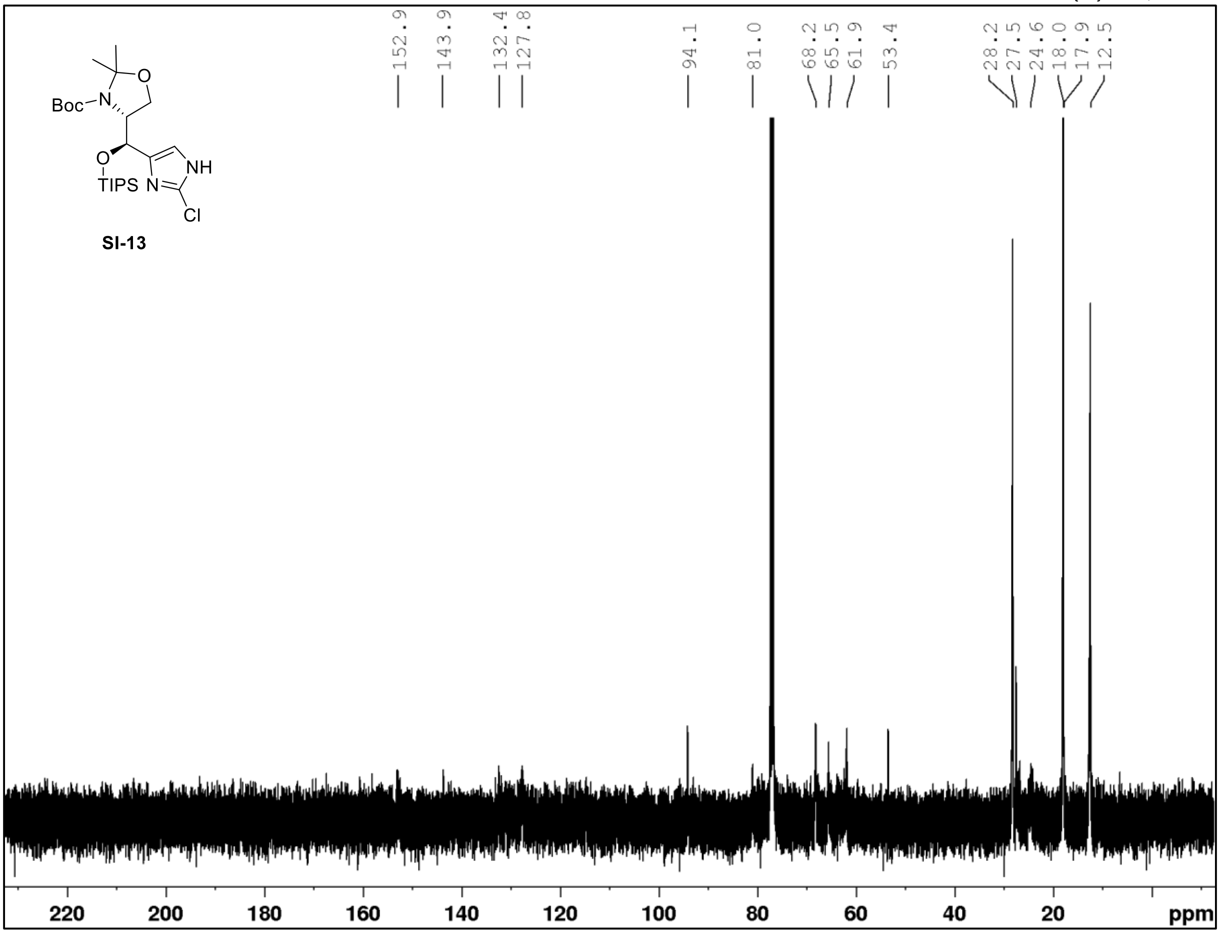

^{1}H-NMR, $CDCl_3$, 400 MHz

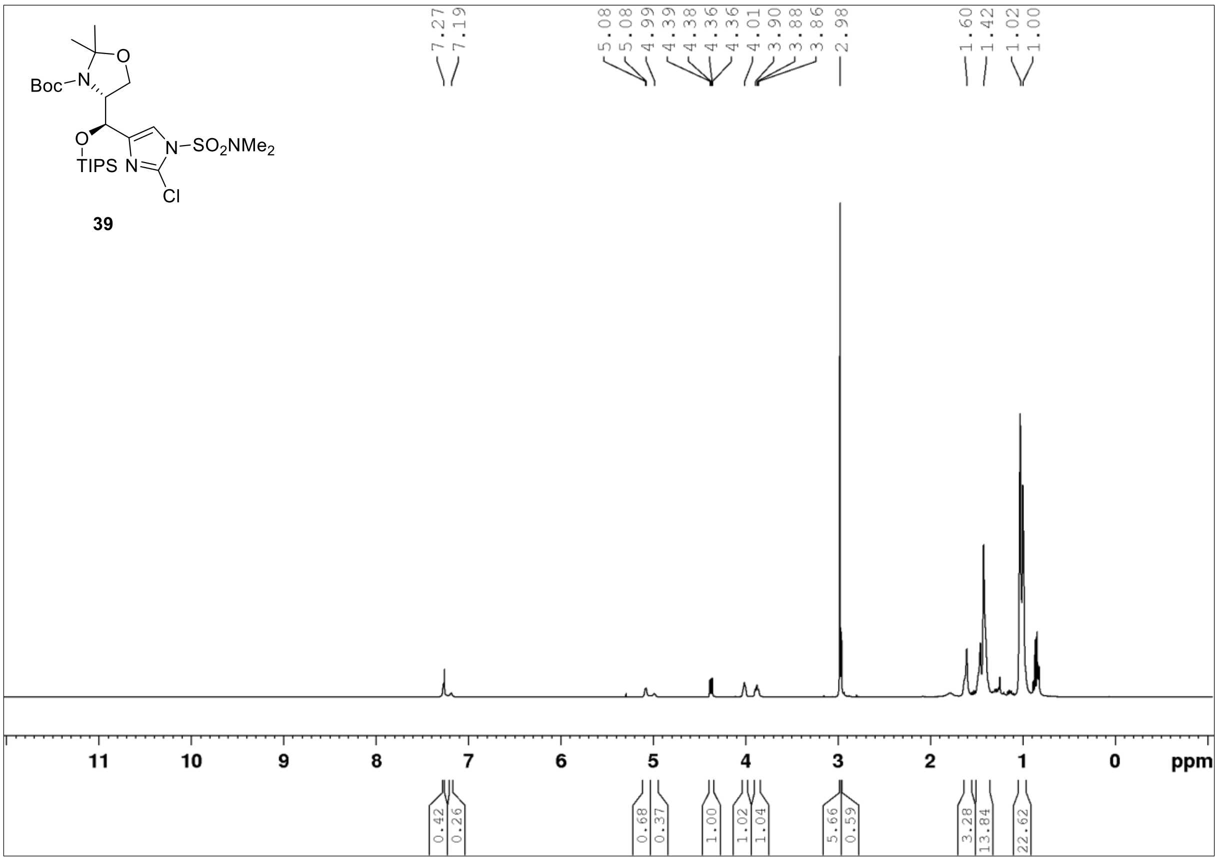

^{13}C{1H}-$CDCl_3$, 100 MHz

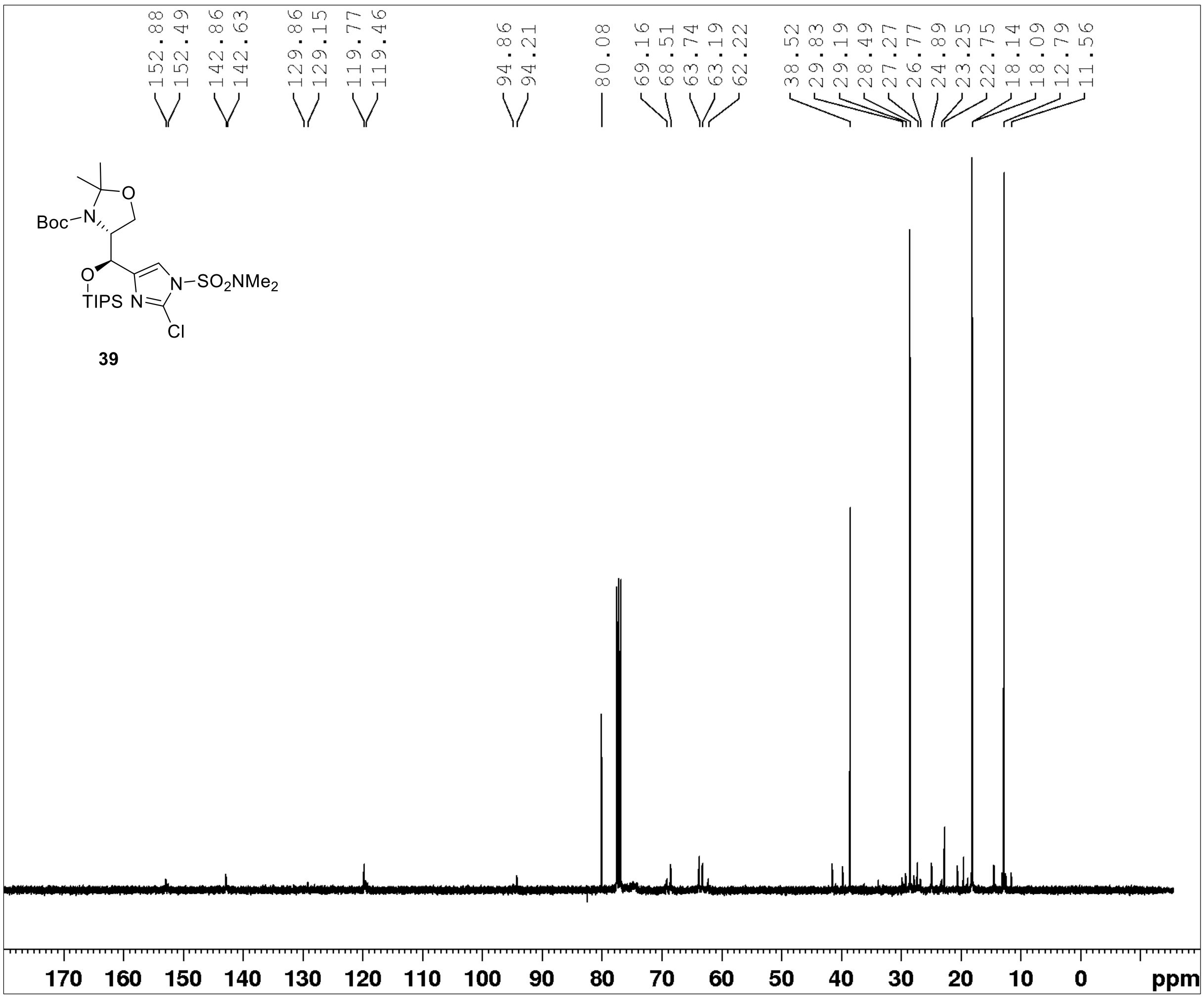

^{1}H-NMR, $CDCl_3$, 400 MHz

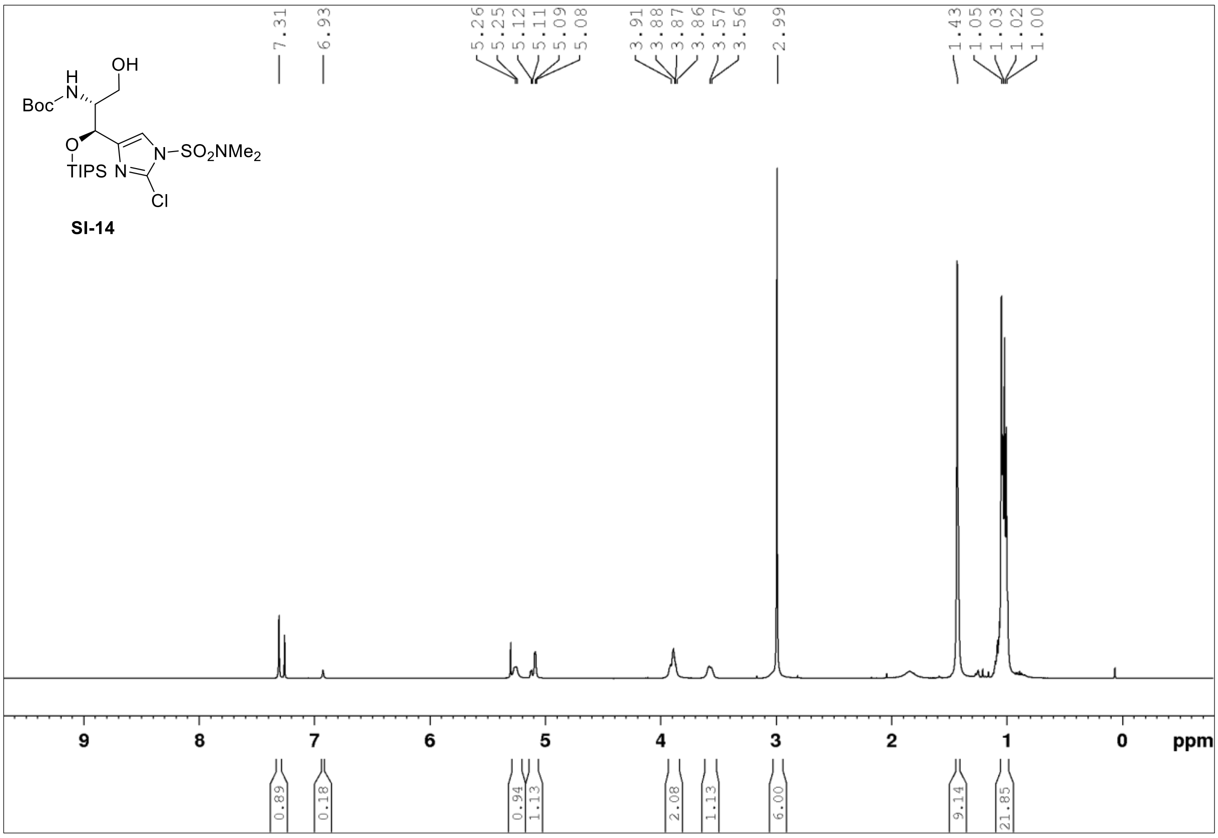

^{13}C{1H}-$CDCl_3$, 100 MHz

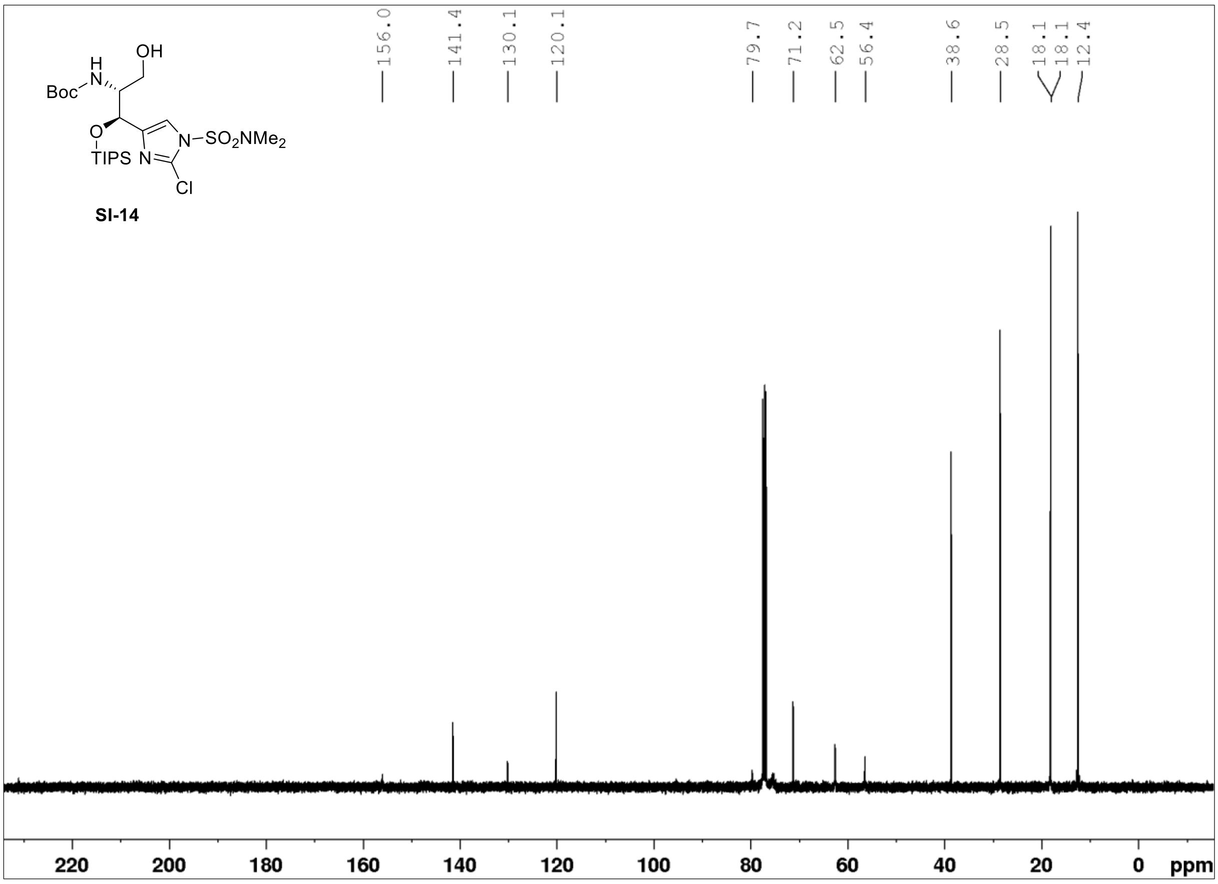

^{1}H-NMR, $CDCl_3$, 400 MHz

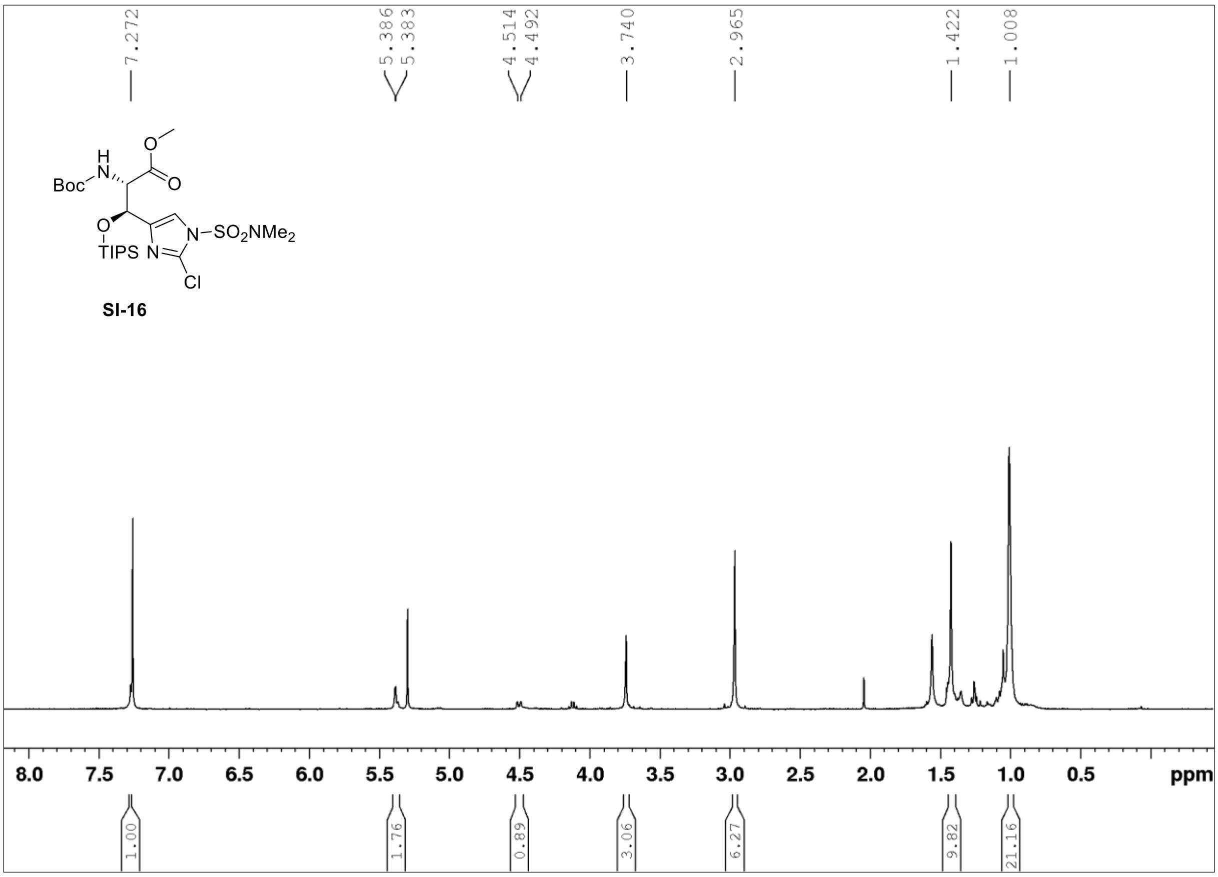

^{13}C{1H}-$CDCl_3$, 100 MHz

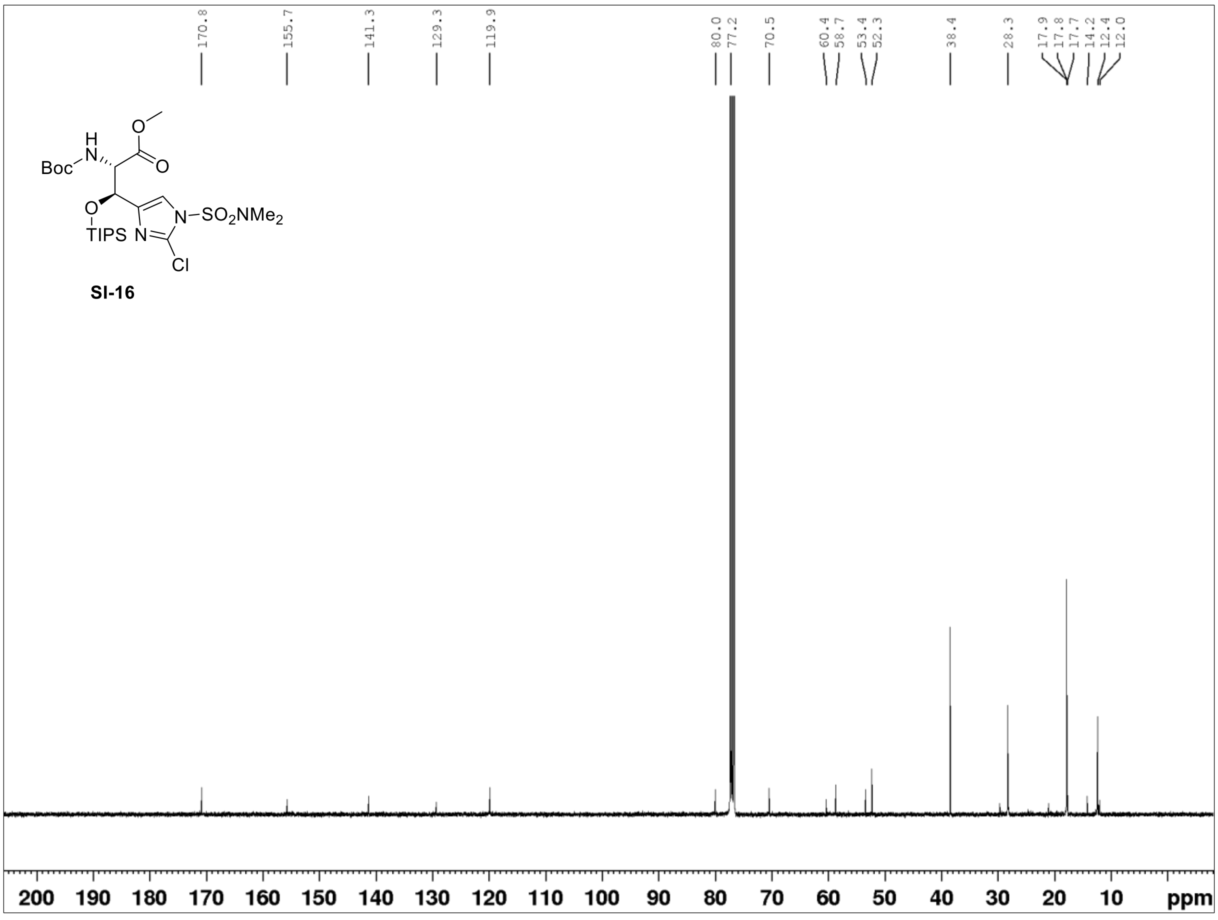

^{1}H-NMR, $CDCl_3$, 400 MHz

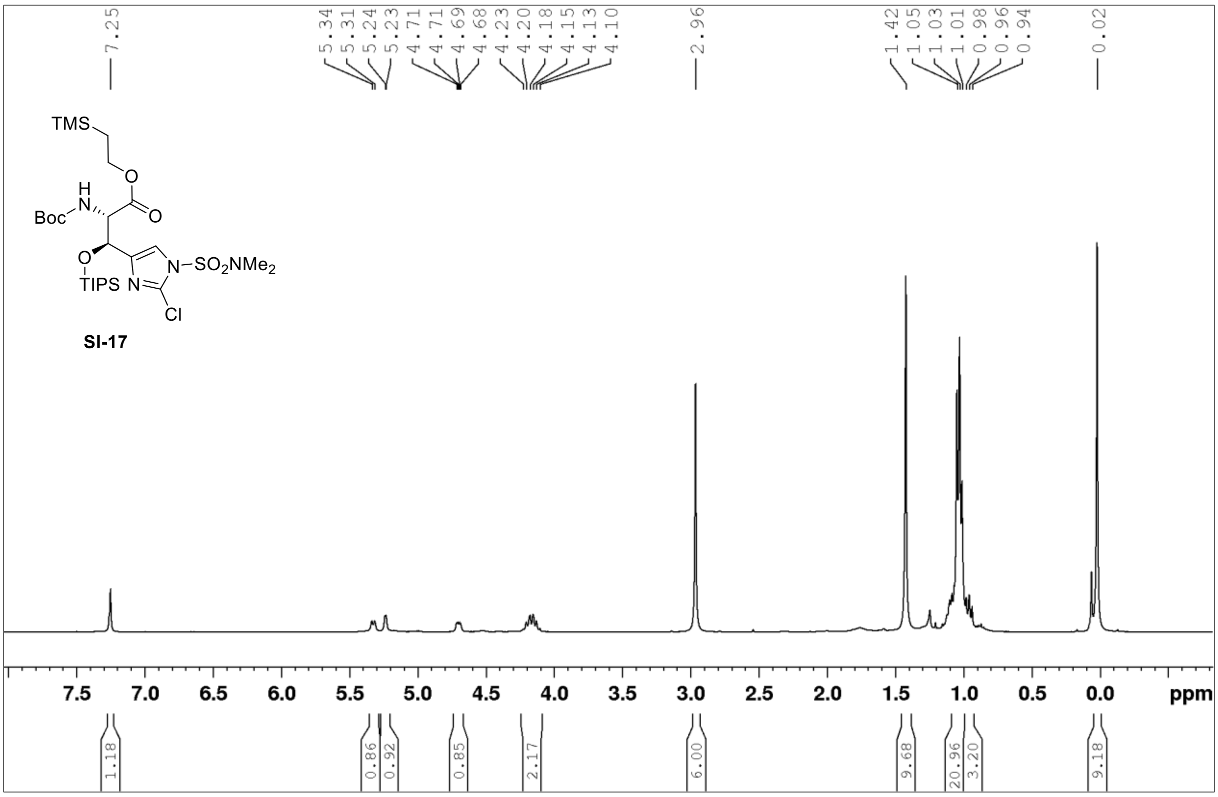

^{13}C{1H}-$CDCl_3$, 100 MHz

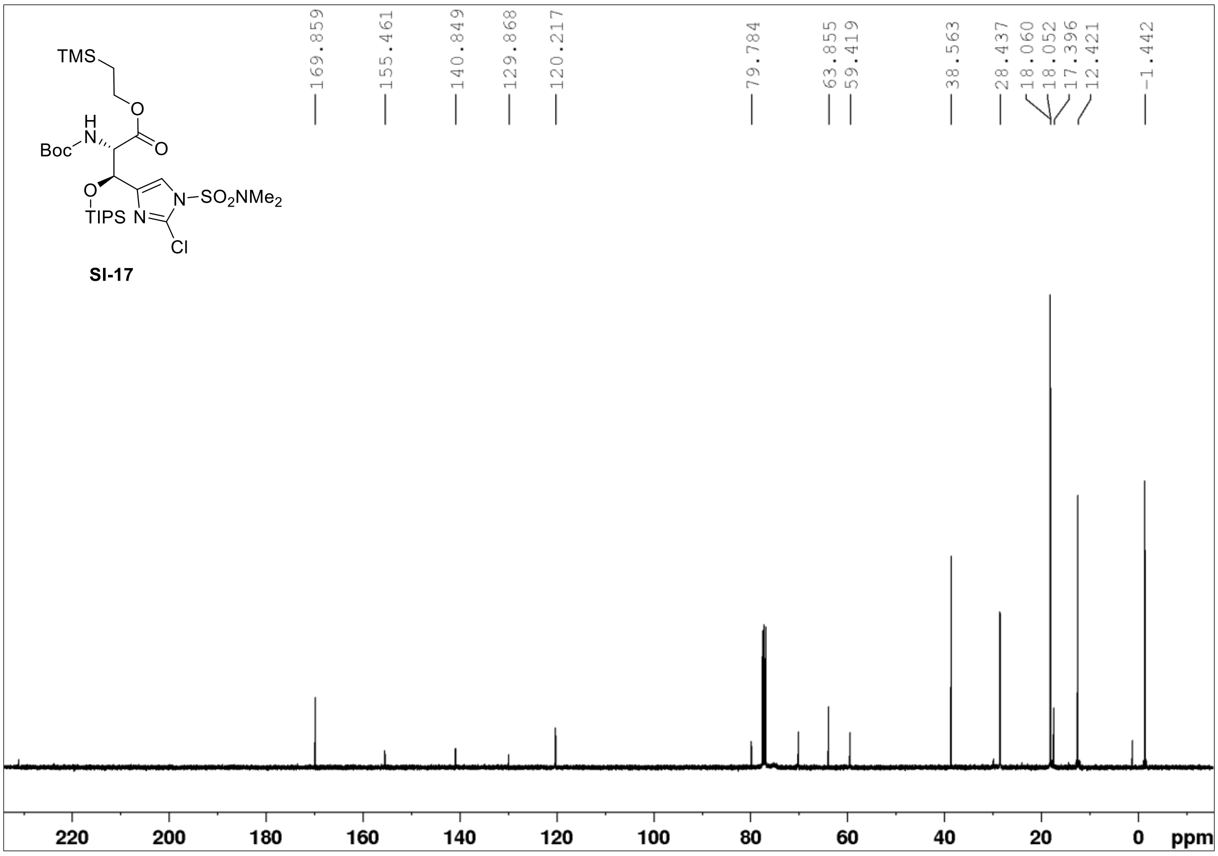

^{1}H-NMR, $CDCl_3$, 400 MHz

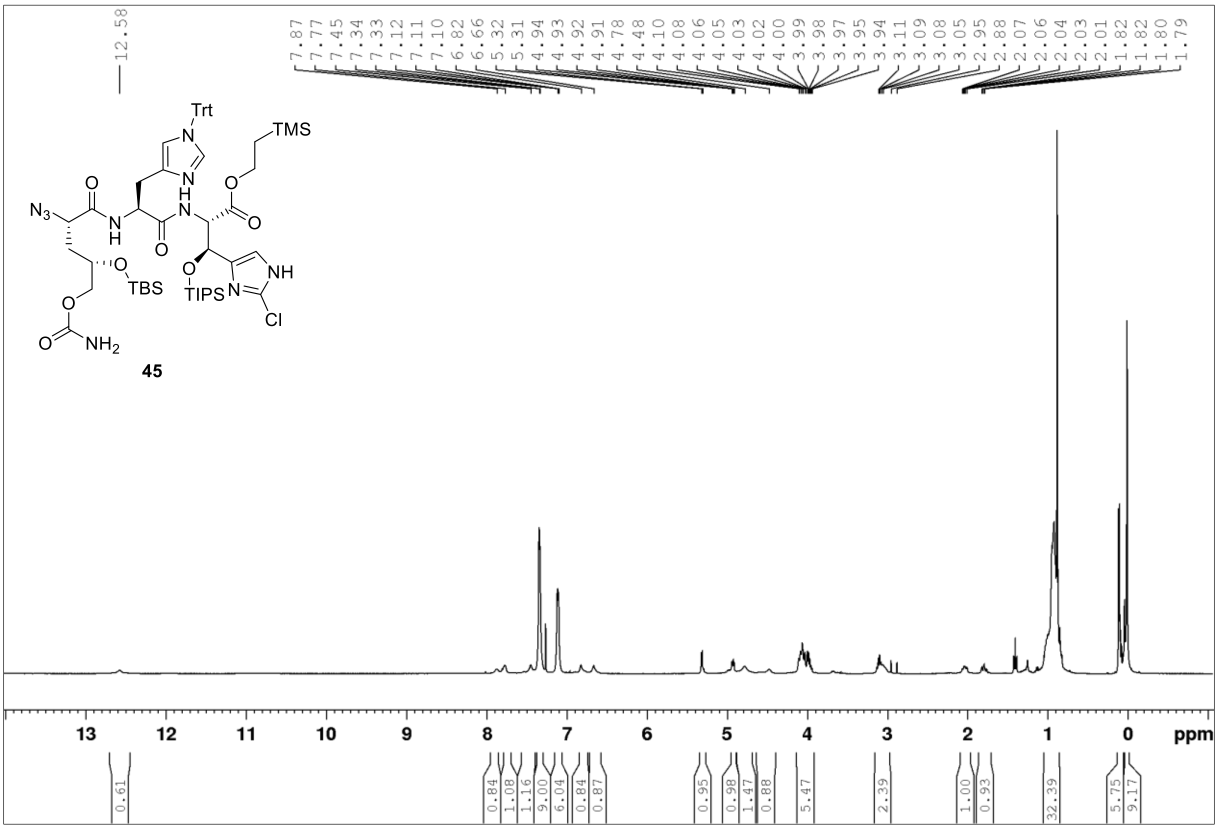

^{13}C{1H}-$CDCl_3$, 100 MHz

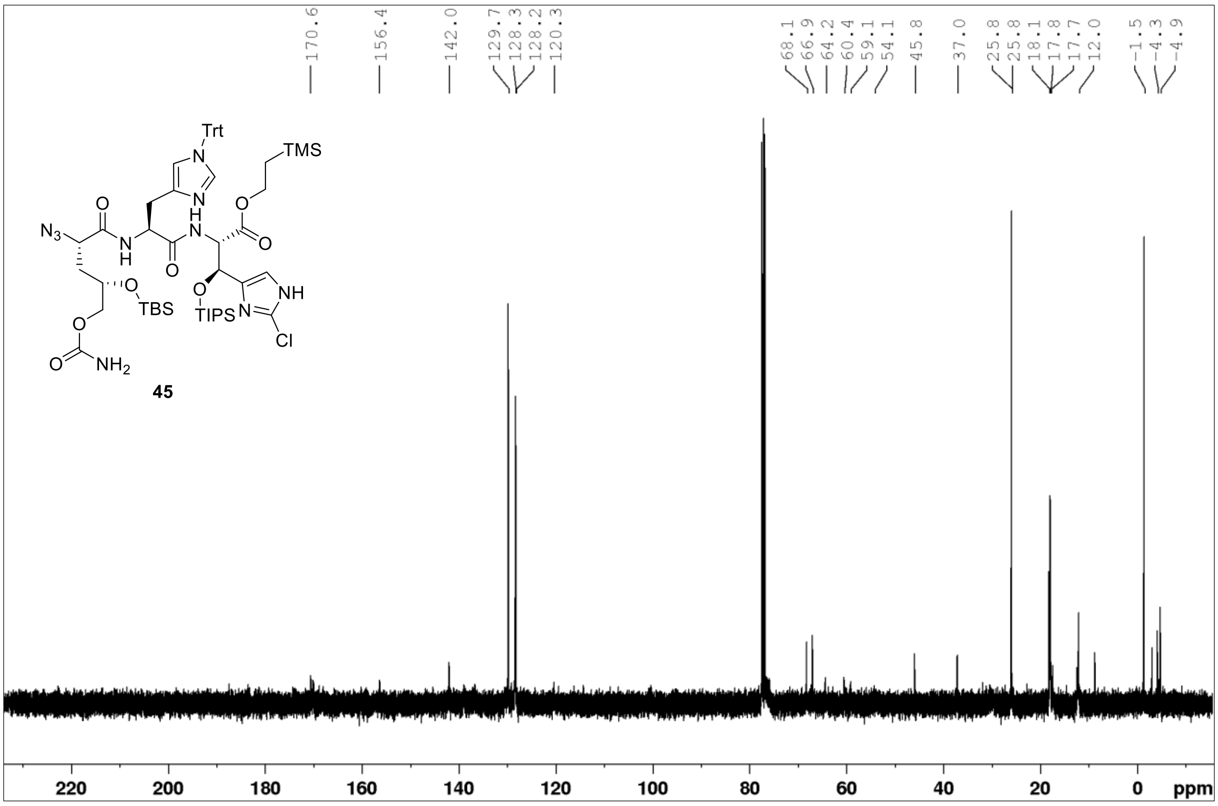

^{1}H-NMR, DMSO-d$_6$, 500 MHz

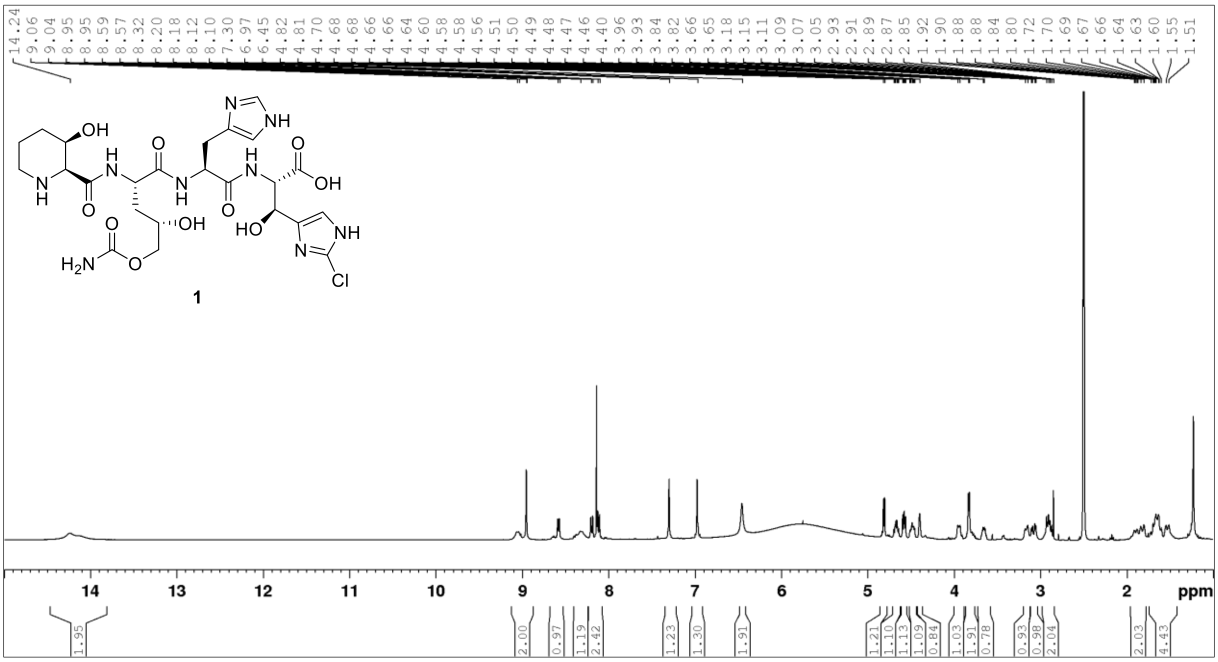

^{13}C{1H}-DMSO-d$_6$, 100 MHz

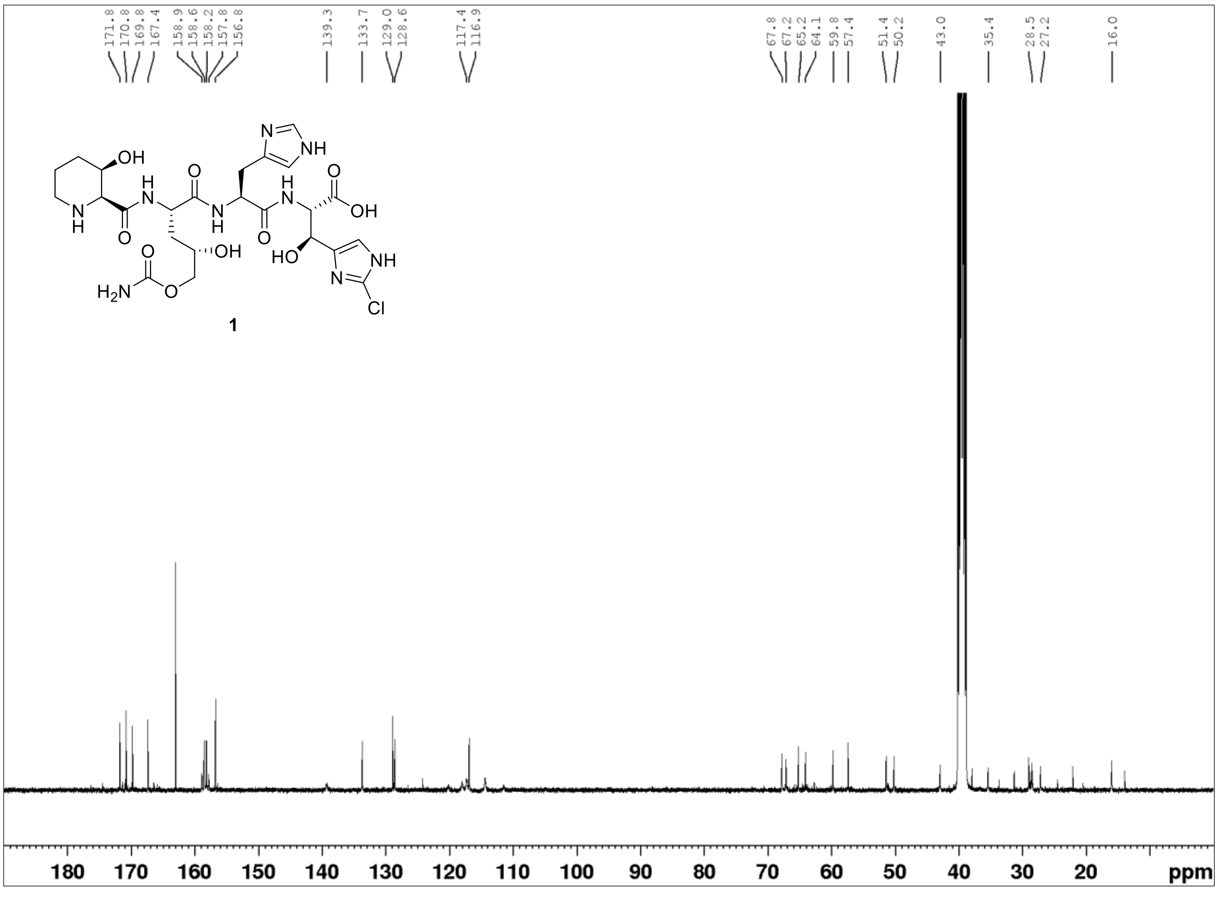

3. Determination of the enantiomeric excesses (*ee*)

SI-3c prepared from L-*N*-Cbz-serine: *ee* = 97%

Chiral Separation

Library, SM, Chiral & Peptide Purification
R&D / IDD In vitro Biology & HT Chemistry
Industriepark Höchst, G 838, Room 007
D-65926 Frankfurt
Dr. Schaffrath Tel.: +49-(0)69-305-30782

SANOFI

Sample Information:

Customer	Dr. Pöverlein
Analyst	K. Rahn-Hotze
Batch Ref No	FFT.OBO1.029.1
Sample Name	FFT.OBO1.029.1
Racemat	
Lab Journal	
Comment	

Separation Information:

APC\Poeverlein\Poeverlein_2018

HPLC-System	LC_11
Flow rate	1,0 ml/min
Temperature	30° C
HPLC Column	Chiralpak AD-H/148, 250x4,6 mm
Eluent	EtOH:MeOH 1:1

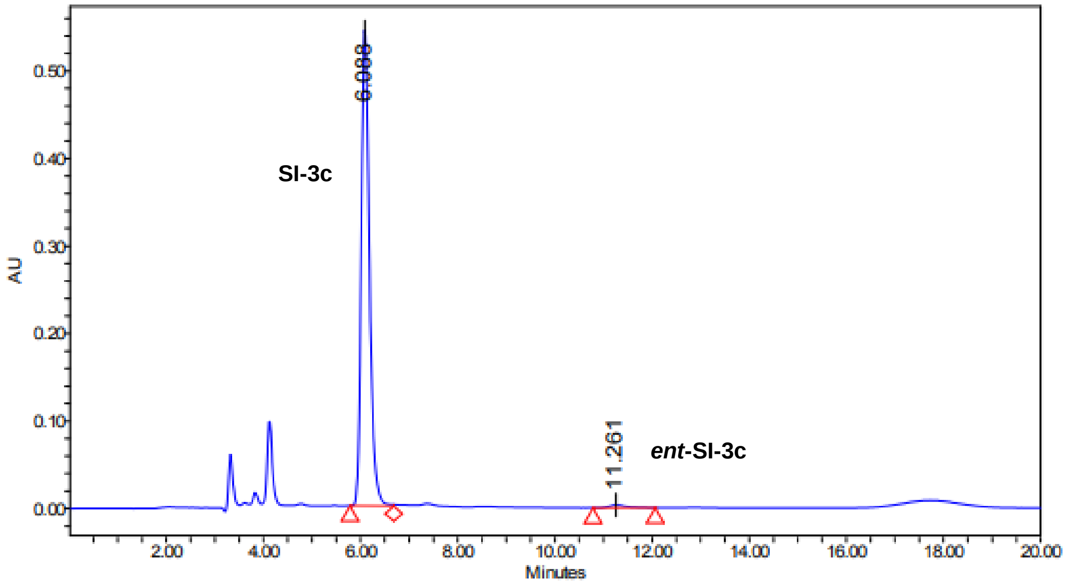

208.0nm; Inj ID 1141; Res ID 1182

Batch_Ref_No FFT.OBO1.029.1 Empower Project: APC\Poeverlein\Poeverlein_2018

Results

	RT	Area	% Area	Height	Resolution
1	6.09	6353638	98.81	543996	
2	11.26	76784	1.19	3077	1.14e+001

***ent*-SI-3c** prepared from D-*N*-Cbz-serine: *ee* = 98%

Chiral Separation

Library, SM, Chiral & Peptide Purification
R&D / IDD In vitro Biology & HT Chemistry
Industriepark Höchst, G 838, Room 007
D-65926 Frankfurt
Dr. Schaffrath Tel.: +49-(0)69-305-30782

SANOFI

Sample Information:

Customer	Dr. Pöverlein
Analyst	K. Rahn-Hotze
Batch Ref No	FFT.OBO1.030.1
Sample Name	FFT.OBO1.030.1
Racemat	
Lab Journal	
Comment	

Separation Information:

APC\Poeverlein\Poeverlein_2018

HPLC-System	LC_11
Flow rate	1,0 ml/min
Temperature	30° C
HPLC Column	Chiralpak AD-H/148, 250x4,6 mm
Eluent	EtOH:MeOH 1:1

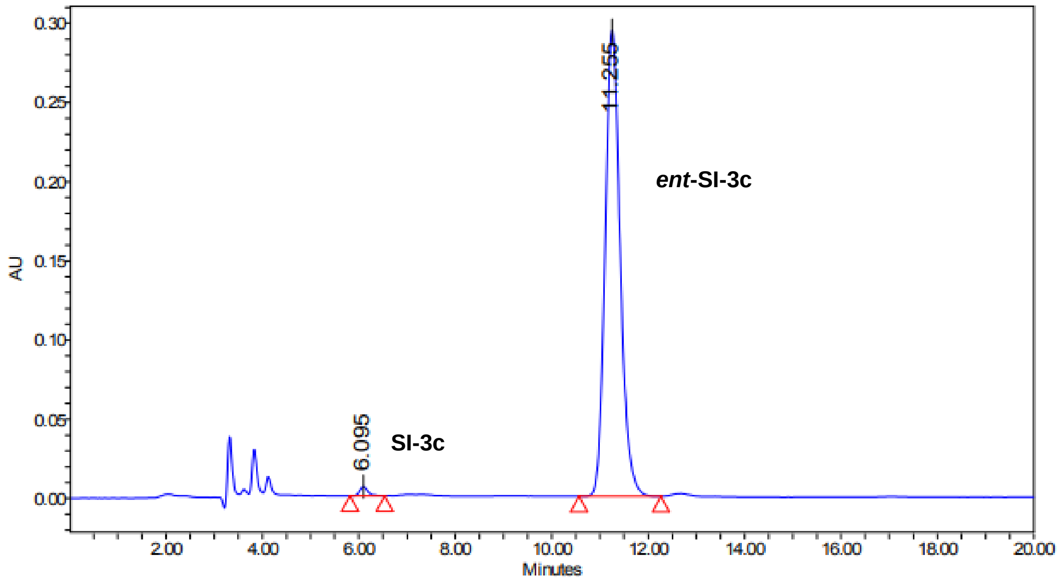

Batch_Ref_No FFT.OBO1.030.1 Empower Project: APC\Poeverlein\Poeverlein_2018

Results

	RT	Area	% Area	Height	Resolution
1	6.10	68651	1.06	5915	
2	11.26	6387674	98.94	294423	1.22e+001

SI-3c prepared from aldehyde **21c** (oxidized according to general procedure B) by reduction with $LiBH_4$ (general procedure C): *ee* = 1%

Chiral Separation

Library, SM, Chiral & Peptide Purification
R&D / IDD In vitro Biology & HT Chemistry
Industriepark Höchst, G 838, Room 007
D-65926 Frankfurt
Dr. Schaffrath Tel.: +49-(0)69-305-30782

SANOFI

Sample Information:

Customer	Dr. Pöverlein
Analyst	K. Rahn-Hotze
Batch Ref No	FFT.OBO1.037.2
Sample Name	FFT.OBO1.037.2
Racemat	
Lab Journal	
Comment	

Separation Information:

APC\Poeverlein\Poeverlein_2018

HPLC-System	LC_11
Flow rate	1,0 ml/min
Temperature	30° C
HPLC Column	Chiralpak AD-H/148, 250x4,6 mm
Eluent	EtOH:MeOH 1:1

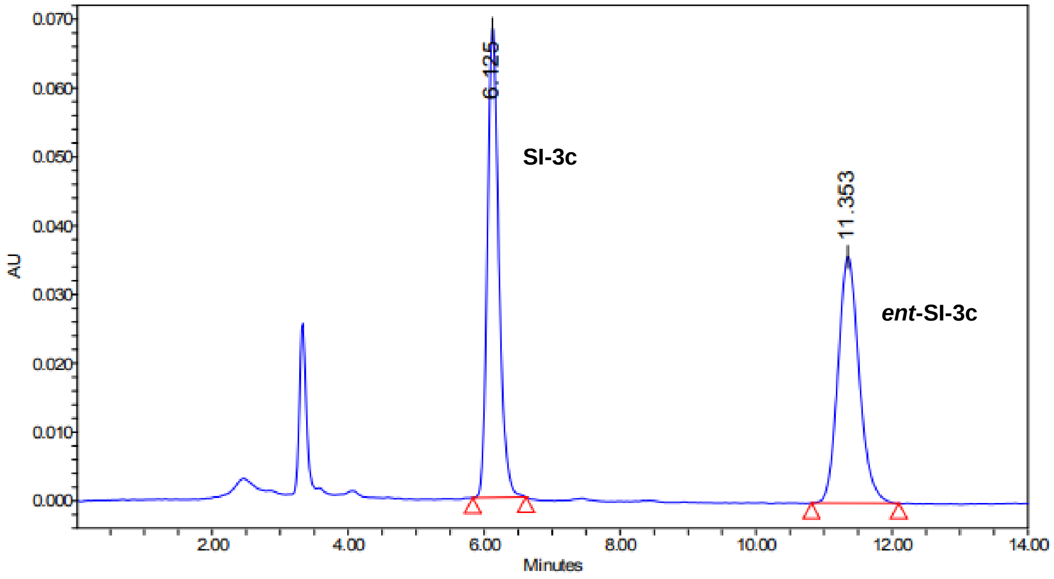

Batch_Ref_No FFT.OBO1.037.2 Empower Project: APC\Poeverlein\Poeverlein_2018

Results

	RT	Area	% Area	Height	Resolution
1	6.13	796323	50.70	68473	
2	11.35	774191	49.30	35845	1.22e+001

SI-3c prepared from aldehyde **21c** (oxidized according to general procedure A) by reduction with $LiBH_4$ (general procedure C): *ee* = 97%

Chiral Separation

Library, SM, Chiral & Peptide Purification
R&D / IDD In vitro Biology & HT Chemistry
Industriepark Höchst, G 838, Room 007
D-65926 Frankfurt
Dr. Schaffrath Tel.: +49-(0)69-305-30782

SANOFI

Sample Information:

Customer	Dr. Pöverlein
Analyst	M.Schnierer
Batch Ref No	FFT.OBO1.085.2
Sample Name	FFT.OBO1.085.2
Racemat	
Lab Journal	
Comment	

Separation Information:

APC\Poeverlein\Poeverlein_2018

HPLC-System	LC_11
Flow rate	1,0 ml/min
Temperature	30° C
HPLC Column	Chiralcel AD-H/148, 250x4,6 mm
Eluent	EtOH:MeOH 1:1

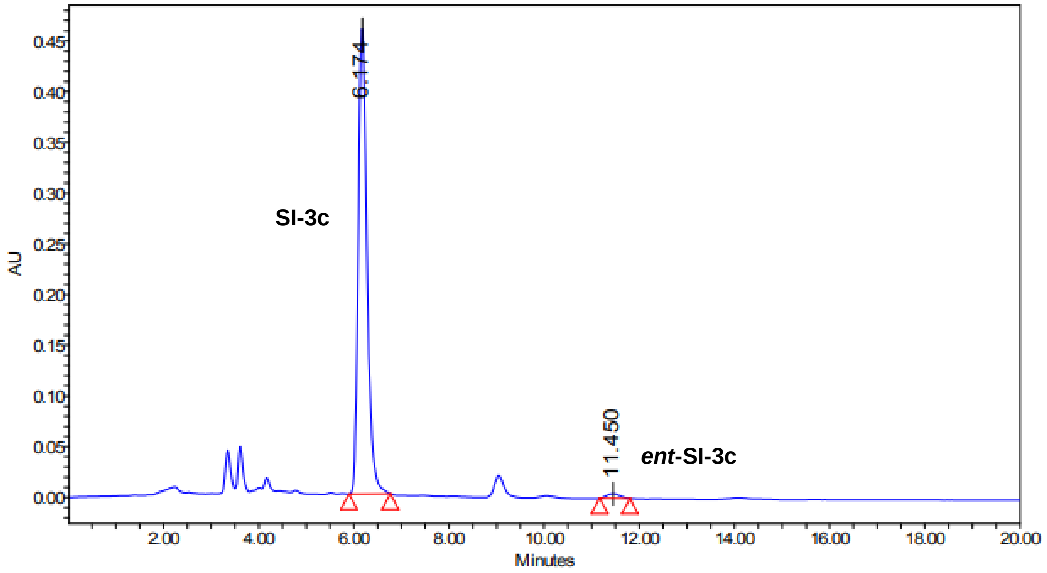

208.0nm; Inj ID 2301; Res ID 2334

Batch_Ref_No FFT.OBO1.085.2 Empower Project: APC\Poeverlein\Poeverlein_2018

Results

	RT	Area	% Area	Height	Resolution
1	6.17	5785055	98.50	460390	
2	11.45	88249	1.50	4659	1.26e+001

Partial racemic reference sample of **19**: *ee* = 15%

Chiral Separation

Library, SM, Chiral & Peptide Purification
R&D / IDD In vitro Biology & HT Chemistry
Industriepark Höchst, G 838, Room 007
D-65926 Frankfurt
Dr. Schaffrath Tel.: +49-(0)69-305-30782

SANOFI

Sample Information:

Customer	Dr. Pöverlein
Analyst	K. Rahn-Hotze
Batch Ref No	FFT.OBO1.041.2
Sample Name	FFT.OBO1.041.2
Racemat	
Lab Journal	
Comment	

Separation Information:

APC\Poeverlein\Poeverlein_2018

HPLC-System	LC_11
Flow rate	1,0 ml/min
Temperature	30° C
HPLC Column	Chiralcel OJ-H/88, 250x4,6 mm
Eluent	EtOH:MeOH 1:1

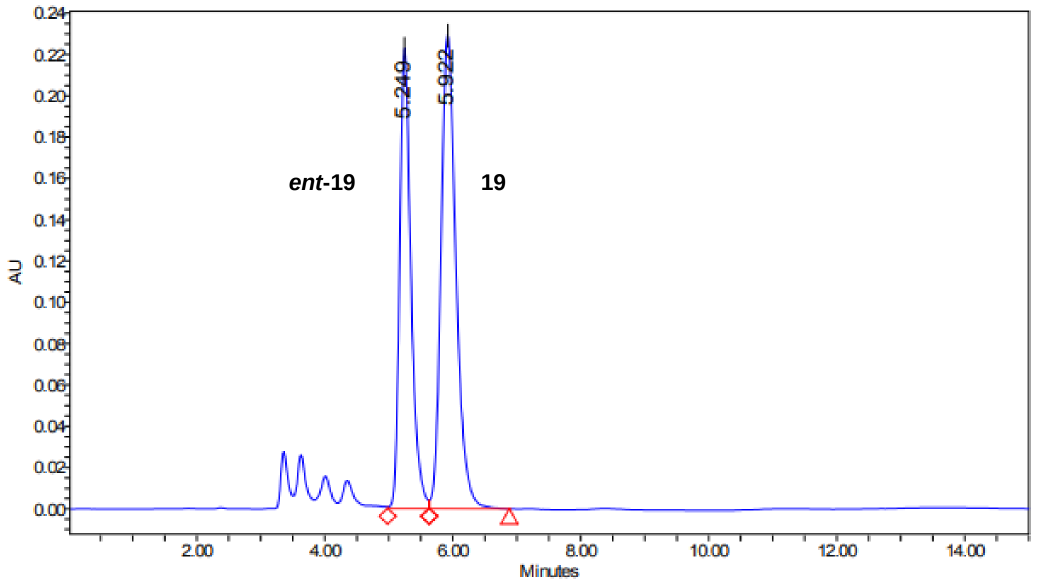

210.0nm; Inj ID 1521; Res ID 1541

Batch_Ref_No FFT.OBO1.041.2 Empower Project: APC\Poeverlein\Poeverlein_2018

Results

	RT	Area	% Area	Height	Resolution
1	5.25	2729768	42.38	222718	
2	5.92	3711668	57.62	229596	1.88e+000

Partial racemic reference sample of **25**: *ee* = 15% (traces of **19** and ***ent*-19** visible)

Chiral Separation

Library, SM, Chiral & Peptide Purification
R&D / IDD In vitro Biology & HT Chemistry
Industriepark Höchst, G 838, Room 007
D-65926 Frankfurt
Dr. Schaffrath Tel.: +49-(0)69-305-30782

SANOFI

Sample Information:

Customer	Dr. Pöverlein
Analyst	K. Rahn-Hotze
Batch Ref No	FFT.OBO1.041.1
Sample Name	FFT.OBO1.041.1
Racemat	
Lab Journal	
Comment	

Separation Information:

APC\Poeverlein\Poeverlein_2018

HPLC-System	LC_11
Flow rate	1,0 ml/min
Temperature	30° C
HPLC Column	Chiralcel OJ-H/88, 250x4,6 mm
Eluent	EtOH:MeOH 1:1

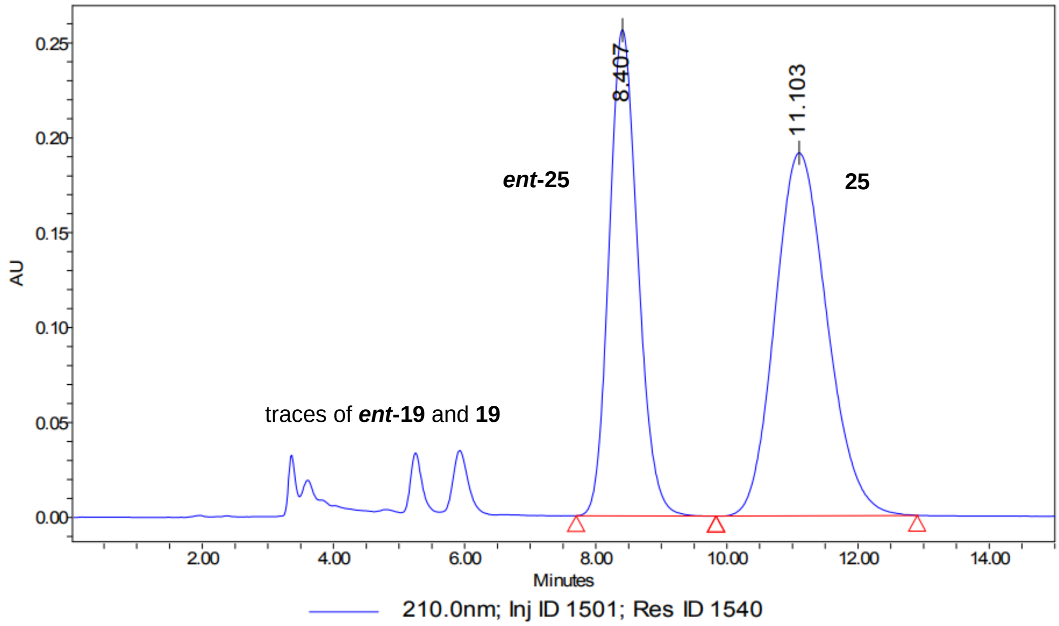

Batch_Ref_No FFT.OBO1.041.1 Empow er Project: APC\Poeverlein\Poeverlein_2018

Results

	RT	Area	% Area	Height	Resolution
1	8.41	7809111	42.45	256085	
2	11.10	10585123	57.55	191256	2.40e+000

Compound **19** prepared by the deprotonation of alkyne **20** with *n*-BuLi and addition to the enantiomeric pure aldehyde **21c** (after recrystallization): *ee* > 99%

Chiral Separation

Library, SM, Chiral & Peptide Purification
R&D / IDD In vitro Biology & HT Chemistry
Industriepark Höchst, G 838, Room 007
D-65926 Frankfurt
Dr. Schaffrath Tel.: +49-(0)69-305-30782

SANOFI

Sample Information:

Customer	Dr. Pöverlein
Analyst	M.Schnierer
Batch Ref No	FFT.OBO1.087.1
Sample Name	FFT.OBO1.087.1
Racemat	
Lab Journal	
Comment	

Separation Information:

APC\Poeverlein\Poeverlein_2018

HPLC-System	LC_11
Flow rate	1,0 ml/min
Temperature	30° C
HPLC Column	Chiralcel OJ-H/88, 250x4,6 mm
Eluent	EtOH:MeOH 1:1

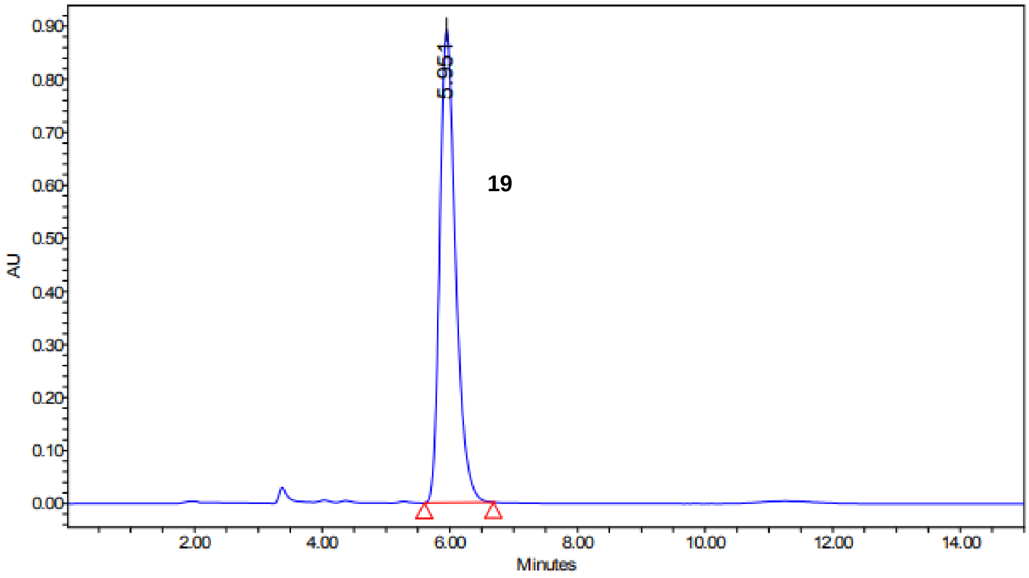

Batch_Ref_No FFT.OBO1.087.1 Empower Project: APC\Poeverlein\Poeverlein_2018

Results

	RT	Area	% Area	Height
1	5.95	15383901	100.00	892960

Mixture of **23a** and **34a** prepared from partial racemic aldehyde **21a**: *ee* = 0%, *dr* > 10:1

Chiral Separation

Library, SM, Chiral & Peptide Purification
R&D / IDD In vitro Biology & HT Chemistry
Industriepark Höchst, G 838, Room 007
D-65926 Frankfurt
Dr. Schaffrath Tel.: +49-(0)69-305-30782

SANOFI

Sample Information:

Customer	Dr. Pöverlein
Analyst	K. Rahn-Hotze
Batch Ref No	FFT.OBO1.108.1
Sample Name	FFT.OBO1.108.1
Racemat	
Lab Journal	
Comment	

Separation Information:

APC\Poeverlein\Poeverlein_2018

HPLC-System	LC_08
Flow rate	1,0 ml/min
Temperature	30° C
HPLC Column	Chiralpak IE/164, 250x4,6 mm
Eluent	Hep:EtOH:MeOH 5:1:1

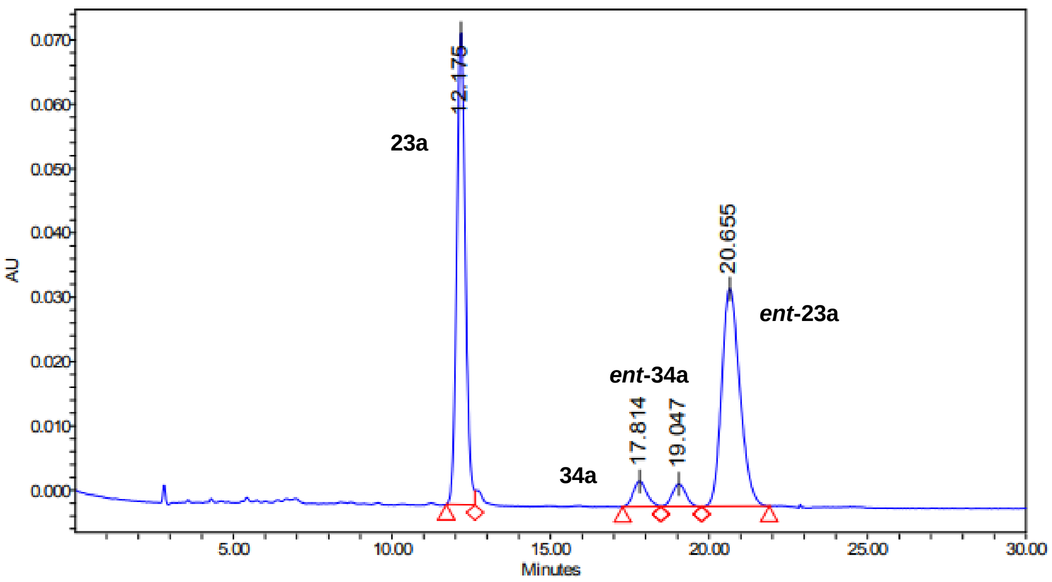

Batch_Ref_No FFT.OBO1.108.1 Empow er Project: APC\Poeverlein\Poeverlein_2018

Results

	RT	Area	% Area	Height	Resolution
1	12.18	1383578	46.86	73358	
2	17.81	116508	3.95	3893	8.85e+000
3	19.05	103598	3.51	3492	1.56e+000
4	20.65	1348765	45.68	33809	1.75e+000

Mixture of **23a** and **34a** prepared from enantiomeric pure aldehyde **21a**: *ee* = 98% (for **23a**), *dr* > 10:1

Chiral Separation

Library, SM, Chiral & Peptide Purification
R&D / IDD In vitro Biology & HT Chemistry
Industriepark Höchst, G 838, Room 007
D-65926 Frankfurt
Dr. Schaffrath Tel.: +49-(0)69-305-30782

SANOFI

Sample Information:

Customer	Dr. Pöverlein
Analyst	K. Rahn-Hotze
Batch Ref No	FFT.OBO1.101.1
Sample Name	FFT.OBO1.101.1
Racemat	
Lab Journal	
Comment	2. Probe

Separation Information:

APC\Poeverlein\Poeverlein_2018

HPLC-System	LC_08
Flow rate	1,0 ml/min
Temperature	30° C
HPLC Column	Chiralpak IE/164, 250x4,6 mm
Eluent	Hep:EtOH:MeOH 5:1:1

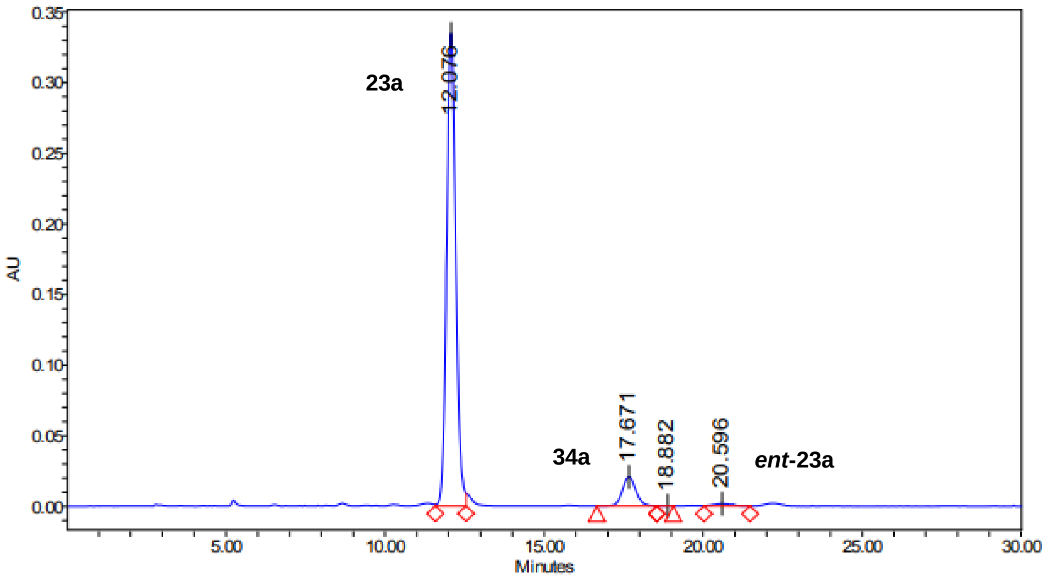

Batch_Ref_No FFT.OBO1.101.1 Empower Project: APC\Poeverlein\Poeverlein_2018

Results

	RT	Area	% Area	Height	Resolution
1	12.08	6520663	90.54	334950	
2	17.67	608136	8.44	20703	8.88e+000
3	18.88	3005	0.04	145	1.63e+000
4	20.60	70245	0.98	1756	1.86e+000

Chiral Separation

Library, SM, Chiral & Peptide Purification
R&D / IDD Purification & Analytics
Industriepark Höchst, G 838, Room 007
D-65926 Frankfurt
Dr. Schaffrath Tel.: +49-(0)69-305-30782

SANOFI

Sample Information:

Customer	Dr. Poeverlein
Analyst	B.Winterhalter
Batch Ref No	FF.ASMJ00210.1
Sample Name	FF.ASMJ00210.1
Racemat	
Lab Journal	
Comment	

Separation Information:

APC\Poeverlein\Poeverlein_2022

HPLC-System	LC_03
Flow rate	1,0 ml/min
Temperature	30° C
HPLC Column	Chiralpak IE/164, 250x4,6 mm
Eluent	Hep:EtOH:MeOH 10:1:1

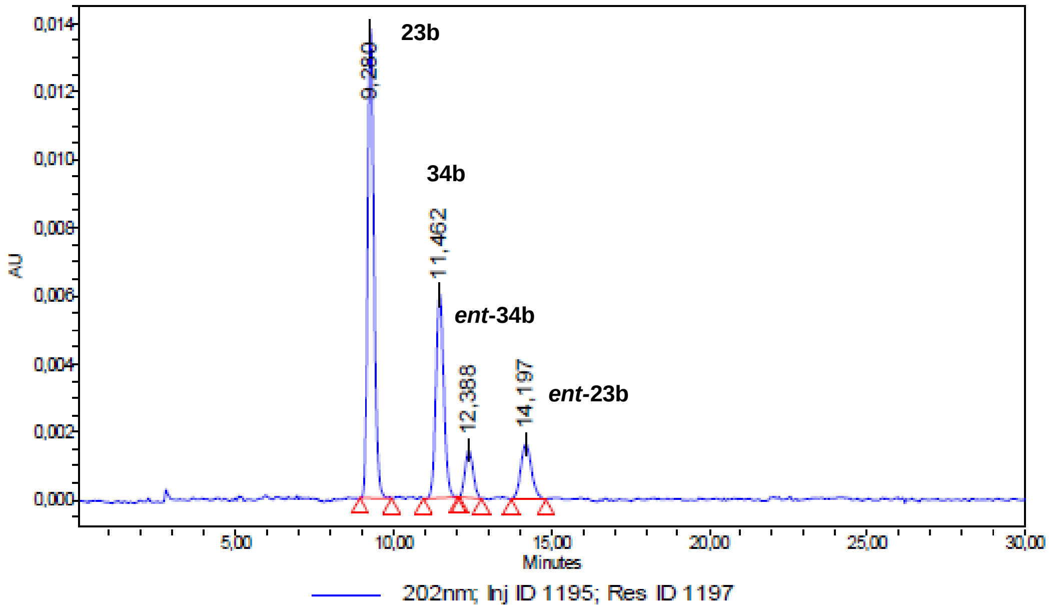

Batch_Ref_No FF.ASMJ00210.1 Empower Project: APC\Poeverlein\Poeverlein_2022

Results

	RT	Area	% Area	Height
1	9,28	192248	53,63	13767
2	11,46	103175	28,78	5975
3	12,39	24709	6,89	1395
4	14,20	38371	10,70	1625

*the depicted chromatogram was the cleanest which could be produced but is not based on a batch with high *ee* of **23b** - these runs always resulted in heavy OBO opening during HPLC; *ee* determination was therefore performed on compound **37**.

Compound **37** prepared from partial racemic aldehyde **21b**: *ee* = 58%

Chiral Separation

Library, SM, Chiral & Peptide Purification
R&D / IDD Analytics & Purification
Industriepark Höchst, G 838, Room 007
D-65926 Frankfurt
Dr. Schaffrath Tel.: +49-(0)69-305-30782

Sample Information:

Customer	Dr. Pöverlein
Analyst	M.Schnierer
Batch Ref No	FF.ASMJ00251.1
Sample Name	FF.ASMJ00251.1
Racemat	
Lab Journal	
Comment	

Separation Information:

APC\Poeverlein\Poeverlein_2022

HPLC-System	LC_26
Flow rate	1ml/min
Temperature	30°C
HPLC Column	Chiralpak IC/172, 250x4,6 mm
Eluent	Hep:EtOH:MeOH 30:1:1

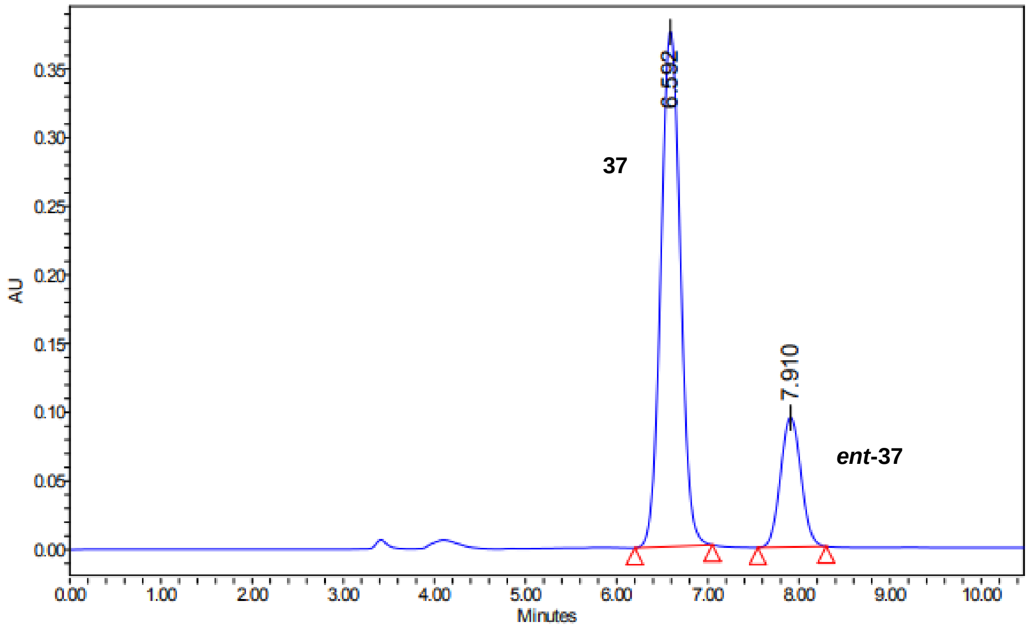

212.0nm; Inj ID 1323; Res ID 1329

Batch_Ref_No FF.ASMJ00251.1 Empower Project: APC\Poeverlein\Poeverlein_2022

Results

	RT	Area	% Area	Height
1	6.59	5587729	79.10	375827
2	7.91	1476362	20.90	94352

Compound **37** prepared from enantiomeric pure aldehyde **21b**: *ee* > 99%

Chiral Separation

Library, SM, Chiral & Peptide Purification
R&D / IDD Analytics & Purification
Industriepark Höchst, G 838, Room 007
D-65926 Frankfurt
Dr. Schaffrath Tel.: +49-(0)69-305-30782

sanofi

Sample Information:

Customer	Dr. Poeverlein
Analyst	M.Schnierer
Batch Ref No	FF.ASMJ00304.2
Sample Name	FF.ASMJ00304.2
Racemat	
Lab Journal	
Comment	analog FF.ASMJ00251.1

Separation Information:

APC\Poeverlein\Poeverlein_2022

HPLC-System	LC_30
Flow rate	1,0 ml/min
Temperature	30° C
HPLC Column	Chiralpak IC/172, 250x4,6 mm
Eluent	Hep:EtOH:MeOH 30:1:1

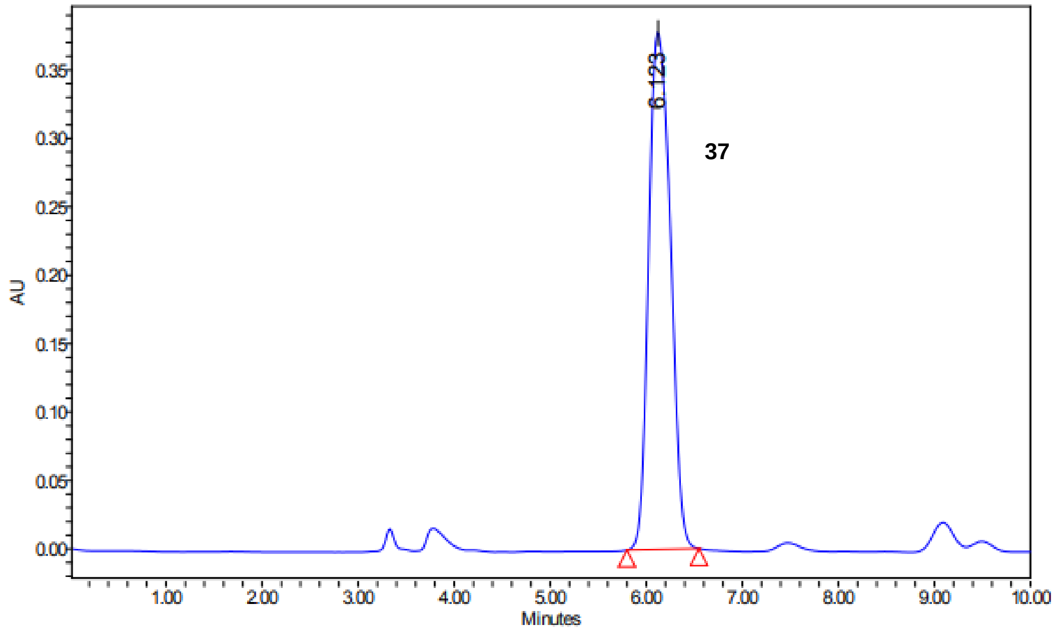

Batch_Ref_No FF.ASMJ00304.2 Empower Project: APC\Poeverlein\Poeverlein_2022

Results

	RT	Area	% Area	Height
1	6.12	5937974	100.00	378702
